# Supplementary material for: Status and future prospect of deregistered woodland key habitats in Northwestern Sweden
Source: PLoS One. 2025 Oct 8;20(10):e0331191. doi: 10.1371/journal.pone.0331191 (PMC12507246; doi:10.1371/journal.pone.0331191)

PLOS ONE

Status and future prospect of deregistered woodland key habitats in northwestern Sweden

--Manuscript Draft--

**Manuscript Number:**	PONE-D-24-09128
**Article Type:**	Research Article
**Full Title:**	Status and future prospect of deregistered woodland key habitats in northwestern Sweden
**Short Title:**	Status and future prospect of deregistered woodland key habitats in northwestern Sweden
**Corresponding Author:**	ulrika Ervander<br>University of Gothenburg: Goteborgs Universitet<br>gothenburg, SWEDEN
**Keywords:**	woodland key habitat; boreal forest; deregistered woodland key habitats; biodiversity; inventories; old growth forests
**Abstract:**	Extensive primary boreal forests within the European Union are primarily located in northwestern Sweden and Finland. These forests host numerous endemic and red-listed species but are exploited rapidly. Over the past thirty years, certain highly valuable areas within Swedish forests have been designated as "woodland key habitats" (WKH) and safeguarded from clear-cutting within certified forests. Despite their high value, WKHs lack proper legislative protection and recently many WKHs were "declassified". In this study we conducted a field inventory of 9 registered and 9 recently de-registered WKHs to asses their conservation values. Additionally, we tested a novel inventory method proposed by the Swedish forest Agency, featuring more stringent criteria. Our inventory results revealed that all 18 areas were classified as WKH using the new method, despite its higher criteria. Analysis of recent harvest actions within deregistered key habitats in Sweden indicated than over 800 hectares of forest cutting occurred within these areas post-deregistration. The study also highlights conflicts between the new inventory method and the national goals for species protection. Since WKH harbor red-listed species in the forest landscape it is crucial to exclude these areas from forestry practices to preserve biodiversity in boreal forests.
**Order of Authors:**	ulrika Ervander
	Willian Arestides Ferreira Da Silva
	Karin Hårding
**Additional Information:**	
**Question**	**Response**
**Financial Disclosure**<br><br>Enter a financial disclosure statement that describes the sources of funding for the work included in this submission. Review the submission guidelines for detailed requirements. View published research articles from *PLOS ONE* for specific examples.<br><br>This statement is required for submission and **will appear in the published article** if the submission is accepted. Please make sure it is accurate.	The author(s) received no specific funding for this work.

**Funded studies**<br>Enter a statement with the following details:<br>• Initials of the authors who received each award<br>• Grant numbers awarded to each author<br>• The full name of each funder<br>• URL of each funder website<br>• Did the sponsors or funders play any role in the study design, data collection and analysis, decision to publish, or preparation of the manuscript?<br><br>Did you receive funding for this work?	
**Competing Interests**<br><br>Use the instructions below to enter a competing interest statement for this submission. On behalf of all authors, disclose any competing interests that could be perceived to bias this work—acknowledging all financial support and any other relevant financial or non-financial competing interests.<br><br>This statement is required for submission and **will appear in the published article** if the submission is accepted. Please make sure it is accurate and that any funding sources listed in your Funding Information later in the submission form are also declared in your Financial Disclosure statement.<br><br>View published research articles from *PLOS ONE* for specific examples.	the authors have declared that no competing interests exist

Powered by Editorial Manager® and ProduXion Manager® from Aries Systems Corporation

**NO authors have competing interests**<br><br>Enter: *The authors have declared that no competing interests exist.*<br><br>**Authors with competing interests**<br><br>Enter competing interest details beginning with this statement:<br><br>*I have read the journal's policy and the authors of this manuscript have the following competing interests: [insert competing interests here]*<br><br>* typeset	
**Ethics Statement**<br><br>Enter an ethics statement for this submission. This statement is required if the study involved:<br><br>• Human participants<br>• Human specimens or tissue<br>• Vertebrate animals or cephalopods<br>• Vertebrate embryos or tissues<br>• Field research<br><br>Write "N/A" if the submission does not require an ethics statement.<br><br>General guidance is provided below. Consult the submission guidelines for detailed instructions. **Make sure that all information entered here is included in the Methods section of the manuscript.**	N/A

**Format for specific study types**<br><br>**Human Subject Research (involving human participants and/or tissue)**<br>• Give the name of the institutional review board or ethics committee that approved the study<br>• Include the approval number and/or a statement indicating approval of this research<br>• Indicate the form of consent obtained (written/oral) or the reason that consent was not obtained (e.g. the data were analyzed anonymously)<br><br>**Animal Research (involving vertebrate animals, embryos or tissues)**<br>• Provide the name of the Institutional Animal Care and Use Committee (IACUC) or other relevant ethics board that reviewed the study protocol, and indicate whether they approved this research or granted a formal waiver of ethical approval<br>• Include an approval number if one was obtained<br>• If the study involved *non-human primates*, add *additional details* about animal welfare and steps taken to ameliorate suffering<br>• If anesthesia, euthanasia, or any kind of animal sacrifice is part of the study, include briefly which substances and/or methods were applied<br><br>**Field Research**<br><br>Include the following details if this study involves the collection of plant, animal, or other materials from a natural setting:<br>• Field permit number<br>• Name of the institution or relevant body that granted permission	
**Data Availability**<br><br>Authors are required to make all data underlying the findings described fully available, without restriction, and from the time of publication. PLOS allows rare exceptions to address legal and ethical concerns. See the PLOS Data Policy and FAQ for detailed information.	Yes - all data are fully available without restriction

A Data Availability Statement describing where the data can be found is required at submission. Your answers to this question constitute the Data Availability Statement and **will be published in the article**, if accepted.<br><br>**Important:** Stating 'data available on request from the author' is not sufficient. If your data are only available upon request, select 'No' for the first question and explain your exceptional situation in the text box.<br><br>Do the authors confirm that all data underlying the findings described in their manuscript are fully available without restriction?	
**Describe where the data may be found in full sentences. If you are copying our sample text, replace any instances of XXX with the appropriate details.**<br><br>• If the data are **held or will be held in a public repository**, include URLs, accession numbers or DOIs. If this information will only be available after acceptance, indicate this by ticking the box below. For example: *All XXX files are available from the XXX database (accession number(s) XXX, XXX.)*.<br>• If the data are all contained **within the manuscript and/or Supporting Information files**, enter the following: *All relevant data are within the manuscript and its Supporting Information files.*<br>• If neither of these applies but you are able to provide **details of access elsewhere**, with or without limitations, please do so. For example:<br><br>*Data cannot be shared publicly because of [XXX]. Data are available from the XXX Institutional Data Access / Ethics Committee (contact via XXX) for researchers who meet the criteria for access to confidential data.*<br><br>*The data underlying the results presented in the study are available from (include the name of the third party*	data are available from following sites or included in the manuscript<br><br>https://skogsgrupp.se/bolagens-borttagna-nb/karta/<br><br>https://www.skogsstyrelsen.se/sjalvservice/karttjanster/geodatatjanster/

and contact information or URL).<br>• This text is appropriate if the data are owned by a third party and authors do not have permission to share the data.<br><br>* typeset	
Additional data availability information:	

Manuscript

Click here to access/download;Manuscript;Manuscript_Ervander Silva Harding_PlosONE_240307.docx

Status and future prospect of deregistered woodland key habitats in northwestern Sweden

Ulrika Ervander[1] , Willian T.A.F. Silva[1,2] and Karin C. Harding[1].

[1]Department of Biology and Environmental Sciences, University of Gothenburg, Gothenburg, Sweden

[2]*Department of Aquatic Resources, Swedish University of Agricultural Sciences, Turistgatan 5, 453 30 Lysekil, Sweden. ORCID: 0000-0002-9517-485X.*

ABSTRACT

Extensive primary boreal forests within the European Union are primarily located in northwestern Sweden and Finland. These forests host numerous endemic and red-listed species but are exploited rapidly. Over the past thirty years, certain highly valuable areas within Swedish forests have been designated as "woodland key habitats" (WKH) and safeguarded from clear-cutting within certified forests. Despite their high value, WKHs lack proper legislative protection and recently many WKHs were "declassified". In this study we conducted a field inventory of 9 registered and 9 recently de-registered WKHs to asses their conservation values. Additionally, we tested a novel inventory method proposed by the Swedish forest Agency, featuring more stringent criteria. Our inventory results revealed that all 18 areas were classified as WKH using the new method, despite its higher criteria. Analysis of recent harvest actions within deregistered key habitats in Sweden indicated than over 800 hectares of forest cutting occurred within these areas post-deregistration. The study also highlights conflicts between the new inventory method and the national goals for species protection. Since WKH harbor red-listed species in the forest landscape it is crucial to exclude these areas from forestry practices to preserve biodiversity in boreal forests.

Keywords: woodland key habitat, boreal forest, deregistered woodland key habitats, biodiversity, inventories.

1.INTRODUCTION

Primary forests and expansive intact forest landscapes worldwide play a crucial role in maintaining global ecological diversity and mitigating the impacts of global warming. Despite growing awareness of their great importance, primary forests continue to decline and become increasingly fragmented at an alarming rate (Potapov et al. 2008, Heino et al. 2015). Approximately 44% of the intact forest landscapes globally consist of boreal forests (Potapov et al. 2008). In Europe, large intact boreal forests are now restricted to northwestern Russia and Fennoscandia (Jonsson 2019, Sabatini 2019, Hansen 2013). In northwestern Sweden there are still old-growth forests that have never been clear-cut, which gives these forests a unique status in the European Union (Jonsson et al. 2019). This green belt of old-growth forest sustains rich biodiversity and holds significant conservation value. Despite their unique ecological importance, the natural forests in northwestern Sweden are constantly diminishing due to high demands for wood and land for establishing new production forests (Svensson et al. 2018, Ahlström et al 2022). The rapidly changing and fragmented forest landscape in boreal Sweden has resulted in a substantial loss of both habitat area and quality of habitat for threatened and 'signal species', i.e., species that are indicators for forests of old age and high biodiversity values. Currently there are 4127 species listed on the Swedish red list, with 2131 of these associated with the forest biotope (Artdatabanken 2015). Notably, 75% of these forest species are red-listed because of the diminishing habitat due to the deforestation of old-growth forests (Artdatabanken 2015). The conversion of continuous primary forests and old-growth forests into clear-cuts and plantations is recognized as having the most pronounced negative impact on biodiversity globally (Larsson et al. 2011, Artdatabanken 2015).

In 1990, the Swedish Forest Agency (Skogsstyrelsen) initiated the identification of Woodland Key Habitats (WKHs) to pinpoint the most valuable areas within the Swedish forests (Nitare 2019, Skogsstyrelsen 2019,a).

Woodland Key Habitats are defined as habitats capable of supporting red-listed species (Skogsstyrelsen 2019,a). On average, more than 20 red-listed and signal species can be found in a WKH (Nitare 2019). Currently, many WKHs are relatively small areas, with an average area of 4,6 hectares (Gustafsson et al. 2018), surrounded by production forests and clear-cut areas. WKHs serve as refuges for threatened species and can host source populations for future recolonization efforts (Djupström 2008). It has been proposed that special attention should be given to forests adjacent to WKHs to enhance the long-term spread and survival of red-listed species (Nitare 2019). An assessment of a recently developed method for classifying WKHs by the SFA, revealed that over 55,000 hectares of WKHs were subjected to clear-cutting between 2000 and 2018 (Skogsstyrelsen 2019,d). The logging of old-growth forests occurs despite the fact that all main forest companies in Sweden are certified according to the standards of the Forest Stewardship Council (FSC) or the Program for the Endorsement of Forest Certification (PEFC). These certifications do not allow harvesting of WKHs (Skogsstyrelsen 2019,d), however, the classification of a forest as a WKH does not confer formal legal protection. Instead, it represents a classification of the forest's conservation values based on inventory results (Skogsstyrelsen 2019,b).

The WKH inventory method relies on an ocular inventory, wherein the surveyor visits a forest identified as a likely WKH on a map and documents the presence and frequency of structures and species associated with old growth forests. The forest history, age, tree layers and elements of high conservation value are documented and these data serve as the basis for the classification of the area (Skogsstyrelsen 2019a). The SFA has developed checklists for northern Sweden in the old inventory method, but these have generally not been consistently and systematically applied. These checklists were primarily developed for surveyors lacking biological training or expertise in classifying a WKH. The aim of the inventory was to promptly identify areas with high nature conservation value using a straightforward and practical method. The application of

these checklists was not based on systematic surveys or test areas (Skogsstyrelsen 2016). Due to substantial regional variations in Swedish forest landscapes, spanning 4000 km from north to south, applying the same method for WKH inventories in the whole country is problematic. Forests in northwestern Sweden differ significantly from forests in other regions in terms of climate (lower temperatures), productivity (lower light incidence), accessibility (difficult terrain), and consequently have a history of less extensive exploitation. Because of that, the proportion of natural old-growth forests is significantly greater in those regions than elsewhere in Sweden and the formally protected areas as well as the voluntary set-aside areas are larger. Registered WKHs are also more common in this region, and it is highly probable that there are numerous still unidentified WKHs and other valuable areas for biodiversity in this inadequately documented region (Skogsstyrelsen 2016).

The inventory method for woodland key habitats has remained relatively unchanged since the start in 1990, with the same method applied across the country. However, in 2017 the Swedish Forest Agency decided to halt the WKH inventory work in northwestern Sweden (Skogsstyrelsen 2019,a), an area identifeid for having the highest density of unprotected old forests (SLU & Sveriges officiella statistik 2018). In 2021 SFA announced that they will stop the WKH inventories for the entire country, shifting the responsibility for identifying these areas to the forest owner. The decision to halt WKH inventories in 2017 was motivated by the need to develop a new method capable of addressing challenges in assessing and delimiting WKHs, particularly in regions with extensive areas of old forests (Claesson 2018). The inventory standstill persisted until January 2018, with the new inventory method being evaluated during 2018 and published in 2019. The new method is more systematic with higher thresholds for an area to qualify as a key habitat in northwestern Sweden (Skogsstyrelsen 2019,a). The classification of an area is determined by specific criteria outlined in

a checklist, which is applied within each area. The result obtained from the checklists should be used as a strong guidance in the classification of a forest, however, ~~it's~~ crucial to emphasize that the classification of a key habitat involves a comprehensive assessment of both the forest and the surrounding landscape. (Skogsstyrelsen 2019a). We investigate the performance of the developed survey method for WKHs in northwestern Sweden and compare the conservation value of registered WKHs and recently ‘’deregistered’’ WKHs. The study highlights the potential consequences that a switch in inventory method and the ongoing deregistration of WKH may have on biodiversity.

2.MATERIALS AND METHODS

2.1 Study area

The fieldwork was carried out in the boreal forests in northwestern Sweden close to the town of Sorsele in 2019. Within the study area, covering a 20 km radius from Sorsele (see Figure 1A), all registered and recently deregistered woodland key habitats(WKH) owned by the state-owned forest company Sveaskog AB were identified on maps obtained from the SFA. Out of the 96 identified areas initially classified as WKHs using the old method, 41 of them have been designated as “deregistered”, and thus lost their status as WKHs. Among these 9 registered and 9 deregistered WKHs were randomly selected and included in the study. To minimize variations arising from company-specific inventory methods, only forests managed by the state-owned forest company, Sveaskog AB, were included.

The software QGIS was used to generate a grid of 200 m x 200 m squares on the map of the study area. The number of sample sites per forest was determined according to the SFA’s guidelines for the new developed inventory method (i.e., forest areas between ~~0,5~~-10 hectares require one sample site for every 2-

3 hectares while a smaller forest requires only one sample site). The specified number of sample sites were randomly distributed across the map (Figure 1 and Table S1).

2.2 Field survey

Each forest was surveyed according to the new method for identifying WKHs in northwestern Sweden. The checklist of parameters includes various factors, such as the density of standing and lying dead wood, the density of trees with conservation value, the number of old spruce trees, hanging lichens and about 30 other descriptors (Figure S1). Red-listed species were also recorded, although it's important to note that the method is not designed as a systematic survey of all red-listed species but rather offers a subsample. The term "conservation tree" is defined as a tree with significant importance for biodiversity such as really old trees (>250 years), trees with hanging lichens or those with holes for birds, for example. Trees that are likely to develop properties that have great importance for biodiversity are also included within the term "conservation tree".

A forest is defined as a WKH if a certain number of criteria specific to forests with high conservation values are met. There are two different routes to reach the status of WKH (track 1 and Track 2): in track 1 a few high-value criteria suffice, while in track 2 a longer list of lower-value criteria is required. For example, in track 1, having 30 conservation spruce trees per hectare is adequate to reach one of the six criteria, whereas in track 2, 20 conservation spruce trees per hectare is required to reach one of the 22 criteria. In northwestern Sweden, a surveyed forest is designated as a WKH if it satisfies five out of the six criteria in track 1. The number of criteria in track 2 that must be fulfilled depends on the location as well as size of the forest: forests smaller than 20 hectares need to fulfill at least nine criteria, whereas larger forests need to fulfill at least eight criteria.

2.3 Statistical and geographical analyses

To asses the potential differences in the number of conservation trees between registered and deregistered forests, a generalized linear model, assuming a Poisson distribution, was fitted to the data. The total number of conservation trees served as the response variable, while registration status and habitat were considered as independent variables. The statistical significance of the effect of the independent variables on the response variable was tested using an analysis of deviance with the Chi-square test. Similarly, using the same tests, we examined whether the total number of red-listed and signal species depended on registration status. All statistical analyses were conducted using the software R.

Geographical analysis was performed using the QGIS geoprocessing tool intersection to combine geographical data for various harvest actions conducted within deregistered key habitats after date of deregistration.

3. RESULTS

3.1 Testing the new inventory method

The results from testing the new inventory method on forests included in this study confirms that all surveyed forests would be classified as WKHs according to the new method for northwestern Sweden. Thus, all currently registered and deregistered WKHs in our study meets the criteria for classification as WKHs under both the old and the new method. Forest D2 (located on Abmoberget) was the only exception that did not fulfill the criteria in boths tracks, but still met the criteria in track 2, which is sufficient for WKH classification (see Table S1 in Appendix for detailed results and scores from inventories.). The map over the area further illustrates that forest D2 is part of a green infrastructure from a landscape perspective. The new method favors large areas and areas in proximity to other WKHs. According to the developed method, a so-called "50/50 rule" can be applied in a continuous forest landscape characterized by high conservation values, especially when borders are hard to delimit. This rule dictates that if 50% of the area reaches the criteria for WKH standard, the entire area should be designated as a WKH. The classified WKH in this continuous forest

landscape has an area of over 450 hectares and the two deregistered forests cover together much less than half of this (less than 1/4th of the size of the surrounding WKH). The 50/50-method thus strengthens the assessment to keep the classification of this forest as a WKH (see figure 1).

The classification of the deregistered WKHs at the time of the field survey is presented in Table S3 (Appendix 1). Forest D2 is classified as a "production forest" and the remaining eight deregistered forests were re-labeled as "non-replaceable promises". A "non-replaceable promise" is a somewhat vaguely defined category of voluntary set-aside area with a higher protection from exploitation through forestry than a regular set-aside area, but which still lacks proper long-term protection from exploitation (Skogsindustrierna 2019). The protection of these areas relies on an agreement between Sveaskog and the county administrative board, but they are not protected by law (Naturvårdsverket 2015). During 2023, however, these 8 ”non-replaceable promises" areas were again reclassified and are now part of a long term protection.

Fig 1. This is the Fig 1 Title: Map of study area.

This is the Fig 1 legend.

Figure 1: The star in map A denotes the location of the study area in northwestern Sweden, with red dots illustrating the distribution of deregistered WKH. Map B in a closeup of the study area, where all WKHs are indicated in blue and deregistered WKH are indicated with red, all surveyed areas are marked with a label. Map C presents deregistered WKH D2 (now classified as production forest), in a landscape perspective. Boundaries of forests of high conservation value according to the County Administrative Board are delineated by a white line. (skogsgruppen 2019, Skogsstyrelsen 2020, Skogsstyrelsen 2019,c, Lantmäteriet 2019)

3.2 Conservation status in registered and deregistered WKH.

A majority of the interior of the surveyed forests displayed no clear indications of human disturbance and had clearly never undergone large-scale exploitation. The biodiversity and conservation value of all forests were notably high, all having more than 100 conservation trees per hectare and many red-listed species. The forests are old, dominated by spruce, with a high amount of dead wood and many old living trees with an age over 250 years and draped with pendulous lichens. Species of conservation interest were not part of an

exhaustive search, but sub sampled during the field study and the index of the number of red-listed and signal species in the studied forests varied from 3 to 21 with an average of 7,4 species per WKH (Appendix 1). The largest deregistered WKH (D6) had the highest number of observed red-listed species, with 18 species belonging to the Swedish red list, of which 5 classified as vulnerable (VU). In the deregistered WKH, D2, currently classified as production forest, nine red-listed species were identified, including two VU species.

3.3 Comparison between findings in studied WKH and set criteria in new inventory method

All surveyed forests exceeded the set criteria of a minimum of 20 conservation trees per hectare for track 1. Additionally, the number of spruce conservation trees was also above the criteria in track 1 for all forests, with at least 30 conservation trees per hectare. The total quantity of dead wood (>10cm) exceeded the set criteria of a minimum of 60 dead trees per hectare in track 1 for all areas, except for WKH D2. As illustrated in Figure 4, current production forest D2 had a relatively low amount of dead wood per hectare. However, this forest exhibited signs of previous wildfire, which could reduce dead wood quantity without diminishing the conservation value of the forest – an observation critical for evaluating the method. To examine potential statistical differences between the two groups of forests (current WKH and deregistered WKH) a significance test was conducted analyzing deviance with a Chi-square test and 17 degrees of freedom. The average number of conservation trees per hectare showed a nearly significant difference between the two groups, with slightly higher density observed for the registered key habitats (see Figure 2). This suggests a probability of approximately 5% that the observed difference between registered and unregistered habitats is a false positive caused by stochasticity in the sampling. From a practical perspective, this p-value is low enough to assume that registered key habitats host a larger number of trees compared to unregistered key habitats. The index of numbers of signal and red-listed species found in the two sorts of habitats was also tested in

the same way and here no significant difference was found between the two groups of forests ($p < 0.08$), see Figure 3.

Fig 2. This is the Fig 2 Title: Conservation trees per hectare**.**

This is the Fig 2 legend.

Figure 2: Variation of the mean number of conservation trees per hectare within each forest. The small box plots show the variation within each forest and the large box plots show the variation within the categories registered WKH (R) and deregistered WKH (D). The significance of the effect of registration status on the mean number of conservation trees per habitat was $p=0.05$. Red line shows criteria for track 1 by the new inventory method, i.e., 20 conservation trees per hectare. It can be noted that all forests are well above the limit of 20 trees per hectare set as a criteria for WKH.

Fig 3. This is the Fig 3 Title: number of red-listed and signal species**.**

This is the Fig 3 legend.

Figure 3: Variation of the number of red-listed and signal species observed during a short visit within each forest (thus not the total number of red listed species). The box plots show the variation within the categories registered WKH (R1-9) and deregistered WKH (D1-9). The Chi-square test between the two groups registered and unregistered WKH shows no significant difference in the index of number of red-listed and signal species ($p < 0.08$).

3.4 Status of WKH and deregistered WKH in Västerbotten county

Two important properties in evaluating the conservation status of a forest are the amount of dead wood and the presence of old trees. In general, the total amount of dead wood and living trees of high age in the studied forests was high compared to the criteria in the inventory method. The total amount of dead wood (both standing and lying) per hectare is shown in figure 4. The number of living spruce trees of old age (>250 years) per hectare is shown in Figure 5. Evaluation of age was done according to the field manual (When using this variable in the developed method), 11 of the 18 forests passed, with more than 10 spruce of old age per hectare, however this variable had a high variation between the sample sites.

Fig 4. This is the Fig 4 Title: Average number of dead wood**.**

This is the Fig 4 legend.

Figure 4: The average total number of dead wood objects per hectare, both standing and lying and standard deviation (SD) with registered showed in purple and deregistered showed in red. The dead wood criteria are 50/hectare in track one (dashed line)and 60/hectare in track two (solid line).

Fig 5. This is the Fig 5 Title: Number of living spruce trees of high age **.**

This is the Fig 5 legend.

Figure 5: Number of living spruce trees of high age >250 years per hectare and standard deviation (SD) in the randomly assigned sample plot in studied forests. Criteria for track 2 (black line) in the developed method is 10 trees per hectare.

Harvest actions within deregistered key habitats since deregistration

The majority of the deregistered key habitats included in this study underwent deregistration between 2015 and 2017. By going through the data base of cutting-reports within the areas of these deregistered key habitats, it has been determined that harvesting activities have been conducted within 874 hectares of deregistered key habitat forest, 300 hectares of which were cut after deregistration.

Fig 6. This is the Fig 6 Title: harvested and reported forest per year **.**

This is the Fig 6 legend.

Figure 6
To the left harvested forest in hectares per year within the deregistered key habitats in Sweden. To the right Forest reported for cutting in hectares per year within the deregistered key habitats in Sweden.

Fig 7. This is the Fig 7 Title: Deregistered key habitats included in the new agreement**.**

This is the Fig 7 legend.

Figure 7

Deregistered key habitats included in the new agreement of permanent protection and the total amount of deregistered key habitats in Sweden (Skogsgruppen 2019).

4. DISCUSSION

Based on the analysis of the field survey we conclude that all the 18 surveyed registered and deregistered WKHs in this study possess the qualities to be classified as a WKH according to the criteria of the new method. These areas represent patches of old forest in a fragmented forest landscape, mostly surrounded by mires, plantations and clear-cuts, without designated buffer zones around the old forests. One of the deregistered WKHs has recently been reclassified as a production forest, while the remaining deregistered WKHs were, at the time of the survey, categorized as ’non-replaceable promise’, a classification without any strict protection. The field survey revealed few or no signs of prior exploitation within these areas. Since the time of the field survey, 8 out of the 9 deregistered WKHs have been reclassified and are now part of a permanent protection plan of valuable mountain forests (Naturvårdsverket 2023). This is however not the case for most of the deregistered WKHs as shown in figure 8.

4.1 Current conservation value of registered and deregistered WKH in Västerbotten

The examined forests exhibited a high amount of dead wood and in many of the forests the spruces were draped in pendulous lichens. Signal and red-listed species were present in all forests, and six of them housed species classified as vulnerable (VU) on the red list. The levels of dead wood and conservation trees were notably high compared to the criteria in the developed method for most forests. A noteworthy observation is that the studied WKHs, whether registered or deregistered, often lack buffer zones to clear-cut areas or new plantations. Considering the observed high conservation value of these forests and the presence of trees with very high conservation values on adjacent clear-cut areas of old forests, the study suggests a need for more frequent inspections before cutting near WKHs. This proactive measure is essential to ensuring that forests with significant nature conservation values are not replaced by clear-cuts followed by planted production forests.

4.2 No significant differences in analyzed conservation values between registered or deregistered WKH

There was a lower, but not statistically significant, quantity of conservation trees in deregistered WKH, and no significant difference was observed in the number of red-listed species. As illustrated in Figure 4, it is evident that the amount of dead wood in all forests is very high compared to the established criteria, even in the new method. With the exception of forest D2, all forests met the criteria in both tracks 1 and 2. The amount of dead wood and old spruce trees were low in D2, contributing to this forest not reaching the requirements in track 1. However, there were signs of a previous forest fire in this forest, which might diminish these characteristics. On the other hand, such fires are known to correlate with high biodiversity values, emphasizing the risk of overlooking crucial forest habitats due to the absence of dead wood following wildfires. All forests in this study satisfy the current criteria to be deemed a WKH, despite the critera beeing stricter than those of the previous method. Valuable forests outside of this study, may be at risk of falling below the criteria of the new method that would have been ~~indirect~~ protected by the old method.

4.3 Deregistered WKHs in Västerbotten and their current classification

All examined deregistered WKHs were classified as likely WKHs according to this study, and eight of them still have the quality to be WKH according to Sveaskog. These eight areas, labelled as ’non-replaceable promises’ to the County Administrative Board after deregistration (Sveaskog 2019d) are regulated through the agreements between Sveaskog and the County Administrative Board in Västerbotten (Naturvårdsverket 2015). Since the time of the field survey these eight forests have been included in a new agreement for long-term protection of mountain forests. This agreement between the Swedish Environmental Protection Agency and Sveaskog, signed in February 2023, protects nearly 100, 000 ~~hecteras~~ of mountain forests, including 48,000 hectares of productive forestland and 50,000 hectares of low productive

forestland(Naturvårdsverket 2023). Forests within the study included in the agreement within their respective areas include; D1,D3,D9 and D4 (Vatioberget), area D5 and D8 (Vatjomyran), area D6 (Välling träsket) and area D7 (Storliden) (Naturvårdsverket 2023). Of the 61 000 hectares of deregistered key habitats only 2100 hectares were included in this agreement of protection. To enhance transparency and secure the long-term protection, we propose that all forests demonstrating the quality of a WKH should remain classified as WKH. This classification serves as a label indicating the conservation value of the forest. A registered WKH does not possess direct legal protection from forestry but is indirectly protected since forest companies certified by FSC are prohibited from harvesting within a WKH (FSC 2010).

The deregistration of WKHs followed by re-classification as production forests, despite having the qualities of WKHs, is also apparent. The deregistered WKH (D2) now classified as a production forest does not only fulfill the criteria for being a likely WKH in track 2 but it is also a part of a green infrastructure directly connected to other WKHs. This should allow the application of the 50/50 rule, an addition to the new method, which states that if there are uncertainties about classification and 50% of the area is of WKH standard, then the entire area should be registered as a WKH. The consequence of neglecting to apply the 50/50 rule to D2 is that this old-growth forest with unique biodiversity values, contributing to a green infrastructure by connecting other segments of old growth forests, can now be subjected to clear-cutting with only six weeks of prior notice.

According to surveys conducted by the Swedish Society for Nature Conservation and by the County Administrative Board in Västerbotten in 2003, D2 (Located in Abmoberget) should qualify as a WKH. The survey report from the County Administrative Board in Västerbotten states that the forest at Abmoberget (see Figure 1) is a large continuous area with very high nature conservation values and any clear signs of forestry are absent throughout the area. The oldest investigated spruce in the entire area reached the age of 497 years (Nygren and Eilertsen 2015). The County Administrative Board argues that the forest landscape on Abmoberget is of high nature conservation value and can be severely damaged by forestry and other

exploitation (Länsstyrelsen Västerbotten 2017). In forest D2, nine red-listed species were found during this short field study and surveys of the greater Abmoberget area found 13 red-listed species and 11 signal species (Länsstyrelsen Västerbotten 2017). The varying conclusions regarding Abmoberget highlight the importance of correctly classifying and delimited a forest. Results from spatial analysis show that harvesting is taking place in these deregistered key habitats.

4.4 Evaluating the developed inventory method

The quality of the WKHs in this so high that the new method, despite higher criteria, did not affect the classification of already existing WKHs. The new method is well structured and, to some extent, aligns with the objectives of transparency, objectivity and predictability. However, certain aspects of the method remain inevitably subjective and dependent on the surveyor. Identifying species of interest, for instance, requires prior knowledge of recognizing them, which may vary among surveyors. For example, some of the species live underneath logs of dead wood or are small and difficult to locate. This places a lot of responsibility on the surveyor and introduces subjectivity to the method. To address this subjectivity, an independent observer could conduct the survey. The new method was in part developed to (better) consider regional differences, however, it does not consider one crucial regional difference: snow. An unfortunate oversight, as the current survey method is impossible to conduct properly without clear visibility of the forest floor, typical effect of witer conditions across northern Sweden. The forests in the inland of Västerbotten, for example, might be covered by snow five months of the year, which stands in contrast to the current rule of a six weeks prior notice before clear cutting. If this period of notice would be extended, regional weather aspects could be better considered, and the forest agency would have more time to survey and identify these valuable forests. The new method includes guidelines that favor large WKHs and those situated near other WKHs. This approach has the potential to enhance the creation of a less fragmented forest landscape, promoting larger areas of old-growth forests that are less vulnerable to species extinction.

4.5 The significance of WKH and the future of WKH inventory

The concept of WKH was not created as a protection form but a classification of the quality of the forest (Skogsstyrelsen 2016). The concept was turned into an indirect protection form by sustainable forest companies who use the WKH concept in their criteria. In the standard for both the Forest Stewardship Council (FSC) and the Program for the Endorsement of Forest Certification (PEFC), there are regulations for certified companies to avoid cutting and selling wood from WKHs. Forest companies certified by FSC are not allowed to cut down any WKH, while companies certified by PEFC must save at least 5% of their woodland where WKHs have the highest priority (FSC 2010; PEFC 2017). In this way the WKH concept indirectly implies a protection status for all certified companies, as a market-driven commitment to save them. The challenge with employing WKH as a protection form lies in the fact that the WKH concept was not originally designed to function as a protected area. Consequently, the WKH concept lacks the inclusion of buffer zones. Another issue arises from the concept's primary focus on identifying small hotspots in the landscape. To ensure a sustainable future for the forest landscape and the species that are depending on it, it is necessary to protect a larger continuous green infrastructure, also incorporating these hotspots (Ericsson et al. 2005, Rybicki and Hanski 2013). To ensure that these valuable areas of old-growth forest won't be lost to production forest we need a common terminology and open communication. Without open access to complete information we cannot see the full picture and are unable to follow the ongoing actions within these forests and unfortunately we see a shift towards even less open access to information about WHK. A complete map of WKH in Sweden is no longer available since many companies have had them removed from the SFA maps and show them only in their own system, making it harder to evaluate ongoing changes. The long term system of finding and registering valuable woodland key habitats occurred between 1990 – 2019. During 2021 it was decided that SFA no longer should identify and register WKHs, resulting in already registered WKH being deregistered since the SFA no longer have the authority to register these as from June 2019.

5. CONCLUSION

The quality of the studied spruce forest woodland key habitats (WKHs) in Västerbotten County is high, with a low level of human disturbance. Despite this, many WKHs in the area have been deregistered. The aim of introducing a new inventory method was to provide an objective, predictable and transparent method, with the goal of enhancing the accuracy and reliability of the method. However, whether the new developed method for the northwestern part of Sweden, featuring higher criteria for WKH (compared to other parts of Sweden) aligns with national and international goals for biodiversity and nature protection, is questionable. A recommended course of action is to grant the Swedish Forest Agency the authority to conduct surveys of all old growth forests before any cutting or thinning activities take place. In addition to regional adaptations in the new method we propose implementing a notice period of at least six snow-free weeks before harvesting a forest. There is also a need of a standardized terminology and a standardized classification system among forest companies, along with open access to information regarding classification of old growth forest areas, ensuring a transparent and secure approach to harvesting old growth forest in Sweden. Deregistered key habitats, with high conservation values, can still be turned into production forests after deregistration, which means that it is important to conduct more assessments of these forests to verify the legitimacy of deregistration and prevent the loss of these valuable ecosystems. As emphasized by the SFA, there are most likely many still unidentified WKHs. These areas must be identified and protected to ensure that Woodland key habitats can persist as refuges for threatened species in the future. The green belt of old growth forests in northwestern Sweden still has the potential to support high biodiversity and are of great importance for adaptation to climate changes, as well as for mitigating the ongoing mass extinction.

Acknowledgments.

We thank Gudrun Norstedt, Frank Götmark and Tero Härkönen for assisting with design of the field study as well as useful comments on the manuscript. Jonas Nordenström is thanked for helping out with species identification and support in the field. Also thanks to Swedish society for nature conservation and the local forest group of Kronoberg for making the map of deregistered key habitats.

REFERENCES

Ahlström, A., Canadell, J. G., & Metcalfe, D. B. (2022). Widespread unquantified conversion of old boreal forests to plantations. Earth’s Future, 10, e2022EF003221. https://doi.org/10.1029/2022EF003221

ArtDatabanken (2015). "Rödlistade arter i Sverige 2015."

Claesson, S. (2018). Nulägesbeskrivning av nordvästra Sverige. kunskapsunderlag. Skogsstyrelsen.

Djupström, L. B., Weslien, J., Schroeder, L. M. (2008). Dead wood and saproxylic beetles in set-aside and non set-aside forests in a boreal region. Forest Ecology and Management, 255: 3340-3350.

Ericsson, Tysk & Berglund, Håkan & Östlund, Lars. (2005). History and biodiversity of woodland key habitats in south boreal Sweden. Biological Conservation. 122. 289-303. 10.1016/j.biocon.2004.07.019.

Esseen Per-Anders and Renhorn Karl-Erik (1998). " Edge Effects on an Epiphytic Lichen in Fragmented Forests." Conservation Biology Vol. 12, No. 6 (Dec., 1998), pp. 1307-1317

FSC 2010 Swedish FSC Standard for Forest Certification including SLIMF indicators FSC-STD-SWE-02-02-2010 Sweden Natural, Plantations and SLIMF EN
FSC. 2019. https://se.fsc.org/se-se/klagoml/offentliga-certifierarrapporter retrieved 2019-12-11

Gustafsson, L., Hannerz, M. 2018. 20 års forskning om nyckelbiotoper – Här är resultaten. Institutionen för ekologi, Sveriges lantbruksuniversitet (SLU), Uppsala. 134 pp.

Hansen, M.C., Potapov, P.V., Moore, R., Hancher, M., Turubanova, S.A.,Tyukavina, A., Thau, D., Stehman, S.V., Goetz, S.J., Loveland, T.R., Kommareddy, A., Egorov, A., Chini., L., Justice, C.O., Townshend, J.R.G. (2013). High-resolution global maps of 21st-century forest cover change. Science 342: 850-853.

Heino, M.; Kummu, M.; Makkonen, M.; Mulligan, M.; Verburg, P.H.; Jalava, M.; Räsänen, T.A. 2015, Forest loss in protected areas and intact forest landscapes: A global analysis. PLoS ONE 2015,10: e0138918.

Jonsson, B., Svensson, J. Mikusiński, Grzegorz, Manton, Michael, Angelstam, P.. (2019). European Union’s Last Intact Forest Landscapes are at A Value Chain Crossroad between Multiple Use and Intensified Wood Production. Forests. 10. 564. 10.3390/f10070564.

Lantmäteriet (2019) Background map for mapping in GIS Retrieved 2019-10-01

Larsson, A. (red) 2011 Tillståndet i skogen – rödlistade arter i ett nordiskt perspektiv. ArtDatabanken Rapporterar 9. ArtDatabanken SLU, Uppsala

Länsstyrelsen Västerbotten 2017, Skogar med höga naturvärden i Västerbottens län - Länsstyrelsens skogsinventeringar nedan gränsen för fjällnära skog 2003–2015

https://www.lansstyrelsen.se/download/18.304d30c1612954524550dee/1526067883061/2017-09-15_Skogar%20med%20h%C3%B6ga%20naturv%C3%A4rden%20i%20V%C3%A4sterbottens%20l%C3%A4n.pdf

Naturvårdsverket. 2015. .Skyddsvärda statliga skogar - Kategorisering av områden på Sveaskogs markinnehav i Västerbottens län. publikation 5497.

Naturvårdsverket 2023. Fjällskog - överenskommelse om långsiktigt skydd https://www.naturvardsverket.se/om-oss/aktuellt/nyheter-och-pressmeddelanden/2023/februari/vardefulla-fjallskogar-skyddas-i-ny-overenskommelse/fjallskogar--overenskommelse-langsiktigt-skydd/ 2023-05-20

Nitare, J. (2019). Skyddsvärd skog Naturvårdsarter och andra kriterier för naturvärdesbedömning. Stibo Graphics, Skogsstyrelsen Jönköping.

Nygren P., Eilertssen L. 2015, Naturskyddsföreningen Västerbottenslän - Abmoberget https://docplayer.se/156084859-Text-patrik-nygren-forutom-avsnitt-6-6-och-6-7-som-skrivits-av-lilleilertsen.html retrieved 2019-12-01

Potapov, P., Yaroshenko, A., Turubanova, S., et al. (2008). Mapping the world's intact forest landscapes by remote sensing. Ecol. Soc., 13, 51, available from: http://www.ecologyandsociety.org/vol13/iss2/art51/. retrieved April 8, 2014.

Potapov P., Yaroshenko A., Turubanova S., Dubinin M., Laestadius L., Thies C., Aksenov D., Egorov A., Yesipova Y., Glushkov I., Karpachevskiy M., Kostikova A., Manisha A., Tsybikova E., Zhuravleva I. 2008. Mapping the World's Intact Forest Landscapes by Remote Sensing. Ecology and Society, 13 (2) Map of Intact forest landscapes. http://www.intactforests.org/data.ifl.html Retrieved 2020-05-01.

Renhorn K.-E. , Esseen P.-A. , Palmqvist K. , Sundberg B. Growth and vitality of epiphytic lichens I. Responses to microclimate along a forest edge-interior gradient Oecologia, 109 (1997), pp. 1-9

Rybicki, Joel & Hanski, Ilkka. (2013). Species-area relationships and extinctions caused by habitat loss and fragmentation. Ecology letters. 16. 10.1111/ele.12065.

Sabatini, F. et al. (2018). Where are Europe's last primary forests?. Diversity and Distributions. 10.1111/ddi.12778.

Skogsindustrierna, (2019). https://www.skogsindustrierna.se/skogsindustrin/hallbarhet/hallbartskogsbruk/frivilligt-avsatt-skog/karta/ retrieved 2019-11-15

Skogsgruppen 2019 https://www.skogsgrupp.se/bolagens-borttagna-nb/ retrieved 2019-06-20

Skogsstyrelsen (2016). "Nulägesbeskrivning om nyckelbiotoper." rapport 7 2016.

Skogsstyrelsen (2019,a). "Handbok för nyckelbiotopsinventering."

Skogsstyrelsen. (2019,b, 2019-07-02). "vårt arbete med nyckelbiotoper."

Skogsstyrelsen (2019,c), https://kartor.skogsstyrelsen.se/kartor/ retrieved 2019-10-01

Skogsstyrelsen (2019,d), utveckling av metod för nyckelbiotopsinventering i nordvästra Sverige.

Skogsstyrelsen (2020) Map of woodland key habitats from Swedish Forest Agency https://www.skogsstyrelsen.se/sjalvservice/karttjanster/ retrieved 2020-01-20.

SLU & Sveriges officiella statistik (2018). Forest statistics 2018, Official Statistics of Sweden Swedish University of Agricultural Sciences. Umeå.

Svensson, J. et al. (2018). Landscape trajectory of natural boreal forest loss as an impediment to green infrastructure. Conservation Biology. 33. 10.1111/cobi.13148.

SUPPORTING INFORMATION

S1- Appendix – Table with inventory information

Appendix

ID	Size	Border (m)	Criteria track 1	Criteria track 2	deregistered (year)	Class-At time of survey	Survey result	Red-listed and signal species (classification from red list)
R1	small	1538	6/6	18/22		WKH	WKH	phlebia mellea(VU), Dryocopus martius(NT), Lobaria pulmonaria(NT), Lobaria scrobiculata(NT), Alectoria sarmentosa(NT), phellinus chrysoloma(NT), Nephroma bellum(LC).
R2	small	10456	6/6	15/22		WKH	WKH	phellinus chrysoloma (NT), Alectoria sarmentosa (NT), fomitopsis rosea(NT)
R3	large*	11222	5/6	16/22		WKH	WKH	Haploporus odorus (VU), Diplomitoporus lenis (VU), Antrodia albobrunnea (VU), Alectoria sarmentosa (NT), Lobaria pulmonaria (NT), Dryocopus martius (NT), phellinus chrysoloma (NT), Lobaria scrobiculatus (NT), Nephroma bellum(LC), Nephroma resupinatum (LC), Icmadophila ericetorum(LC).
R4	large	14802	6/6	15/22		WKH	WKH	Alectoria sarmentosa (NT), phellinus chrysoloma (NT), Picoides tridactylus(NT), pseudographis pinicola (NT)
R5	small	5402	6/6	12/22		WKH	WKH	Alectoria sarmentosa (NT), phellinus chrysoloma (NT), Lobaria scrobiculata(NT), Icmadophila ericetorum(LC).
R6	large	4714	5/6	14/22		WKH	WKH	Alectoria sarmentosa (NT), Dryocopus martius (NT), Cystostereum murrayi(NT), Phellinus chrysoloma (NT), Lobaria scrobiculatus (NT), Nephroma resupinatum (LC).
R7	large	6925	6/6	16/22		WKH	WKH	Phellinus chrysoloma (NT), Alectoria sarmentosa (NT), Perisoreus infaustus(LC), Matteuccia struthiopteris(LC).
R8	large*	6386	6/6	18/22		WKH	WKH	Fomitopsis rosea (NT), Leptoporus mollis(NT), Picoides tridactylus(NT), Alectoria sarmentosa (NT), Pseudographis pinicola (NT), phellinus chrysoloma (NT), Perisoreus infaustus (LC), Nephroma bellum(LC).
R9	large	9431	6/6	16/22		WKH	WKH	Cystostereum murrayi(NT), Alectoria sarmentosa (NT), phellinus chrysoloma (NT), Onnia leporina(NT), Perisoreus infaustus(LC), Icmadophila ericetorum (LC), Aconitum lycoctonum (LC), Icmadophila ericetorum (LC), Nephroma resupinatum (LC).
D1	large*	4259	6/6	16/22	2015	promise	WKH	Alectoria sarmentosa (NT), phellinus chrysoloma (NT), Icmadophila ericetorum (LC).
D2	large	5470	2/6	12/22	2017	product-ion	WKH	Haploporus odorus (VU), skeletocutis odora (VU), Phellinidium ferrugineofuscum (NT), Carbonicola anthracophila (NT) , Dryocopus martius (NT), pseudographis pinicola (NT), Alectoria sarmentosa (NT), Phellopilus nigrolimitatus (NT), Onnia leporina (NT), Nephroma resupinatum (LC).
D3	large*	2871	6/6	18/22	2015	promise	WKH	Poecile cinctus (VU), Alectoria sarmentosa (NT), phellinus chrysoloma (NT), Onnia leporina(NT),Picoides tridactylus (NT) Nephroma bellum (LC), Nephroma resupinatum (LC), Meruliopsis taxicola (LC).
D4	large	5981	6/6	18/22	2015	promise	WKH	Skeletocutis odora(VU), fomitopsis rosea (NT), phellinus chrysoloma (NT), Alectoria sarmentosa (NT), Phellinidium ferrugineofuscus (NT), Leptoporus mollis (NT), Perisoreus infaustus(LC), Icmadophila ericetorum (LC).
D5	large	10436	6/6	15/22	2015	promise	WKH	Alectoria sarmentosa (NT), Phellinidium ferrugineofuscus,(NT) fomitopsis rosea (NT), Onnia leporina(NT), phellinus chrysoloma (NT), Phellopilus nigrolimitatus (NT), Icmadophila ericetorum (LC).
D6	large	18606	5/6	18/22	2015	promise	WKH	Antrodia albobrunnea (VU), Diplomitoporus lenis (VU), Skeletocutis odora(VU), Phlebia mellea(VU), Laurilia sulcata(VU), Onnia leporina (NT), Alectoria sarmentosa (NT), phellinus chrysoloma (NT), pseudographis pinicola (NT), Cystostereum murrayi (NT), Leptoporus mollis (NT), Phellinidium ferrugineofuscum (NT), Lobaria pulmonaria (NT), fomitopsis rosea (NT), Phellopilus nigrolimitatus (NT), Picoides tridactylus (NT), Hypogymnia bitteri (NT), Icmadophila ericetorum (LC), Meruliopsis taxicola(LC), Perisoreus infaustus (LC), Nephroma bellum(LC).
D7	small	1616	6/6	13/22	2015	promise	WKH	Phellinus ferrugineofuscus (NT), Alectoria sarmentosa (NT), Onnia leporina (NT), fomitopsis rosea (NT), Nephroma bellum(LC).
D8	large	17585	6/6	16/22	2015	promise	WKH	Laurilia sulcata (VU), Phellinidium ferrugineofuscum (NT), fomitopsis rosea (NT), Cystostereum murrayi (NT), phellinus chrysoloma (NT), Phellopilus nigrolimitatus (NT), Alectoria sarmentosa (NT), Picoides tridactylus (NT), Icmadophila ericetorum(LC), Perisoreus infaustus (LC),
D9	large*	1445	6/6	15/22	2015	promise	WKH	Alectoria sarmentosa (NT), phellinus chrysoloma (NT), pseudographis pinicola (NT), Nephroma bellum(LC)

Table S1: All 20 forests randomly selected from the study area. Size is a category of importance when performing the developed method. A spruce forest >20 hectares are considered large and a spruce forest < 20 hectares are considered small. The exception is if the forest is closer than 100m to another key habitat the size of that key habitat is added and if the sum together becomes larger than 20 hectares the forest is considered large*. Table also shows results from the WKH survey with the developed method. Criteria track 1 - forest likely a WKH if more than 5 criteria are fulfilled. Criteria track 2 - forest likely a WKH if more than eight or nine criteria are fulfilled (depending on the size and surrounding). All findings of red-listed and signal species from field survey.

Figure

Click here to access/download;Figure;Figure 1.tiff

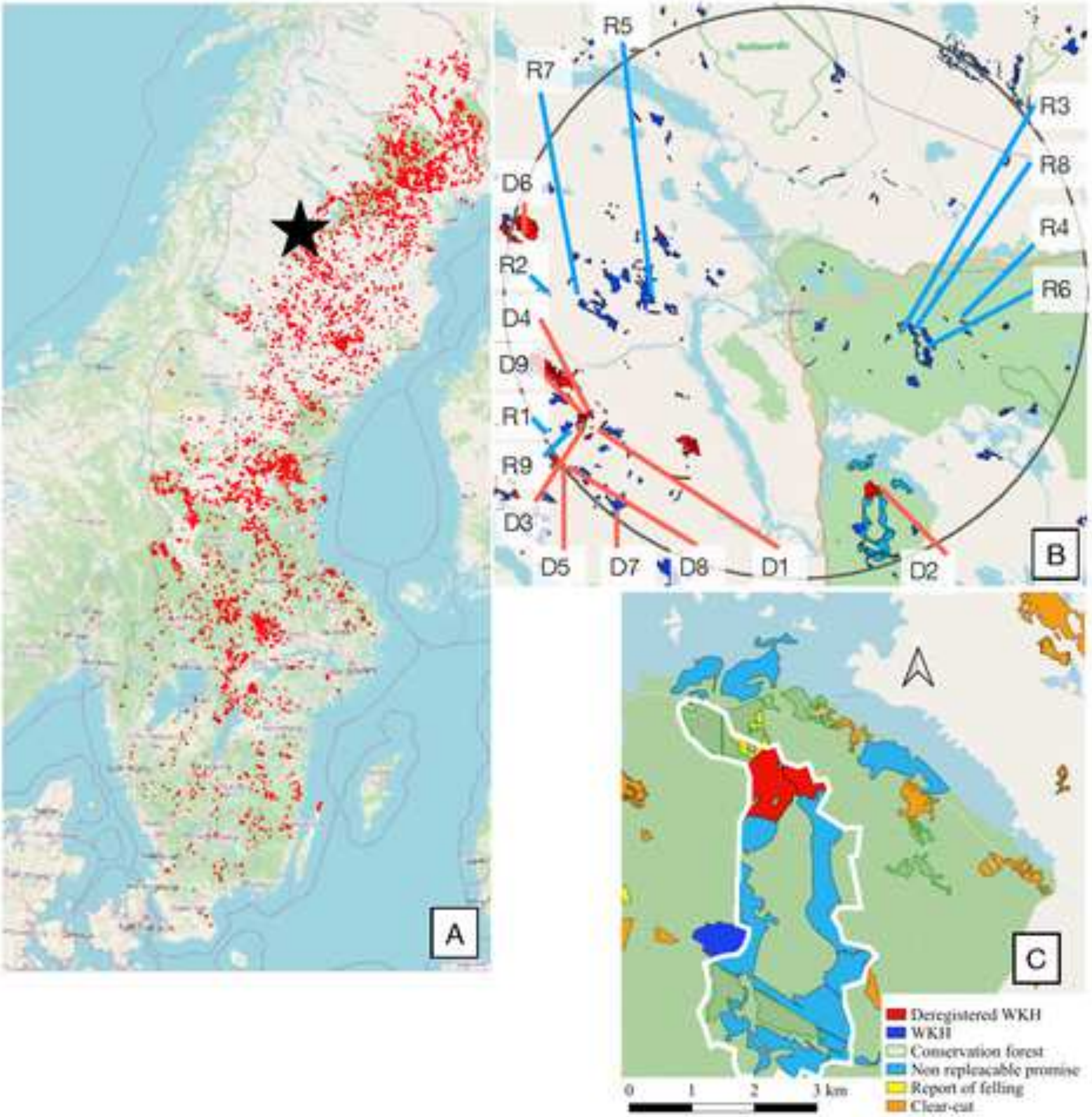

 Click here to access/download;Figure;figure 2.tiff

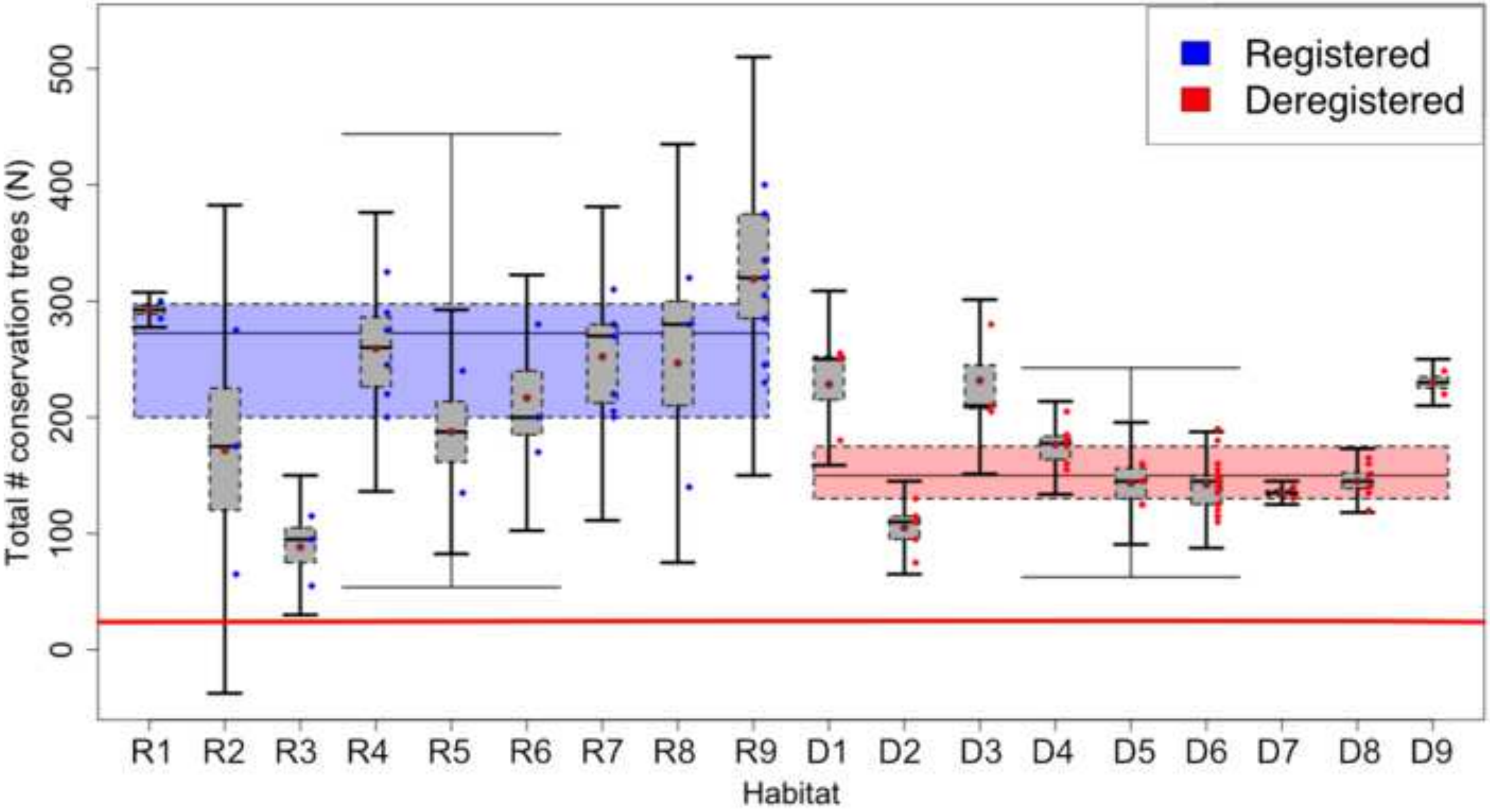

Figure

Click here to access/download;Figure;figure 3.tiff

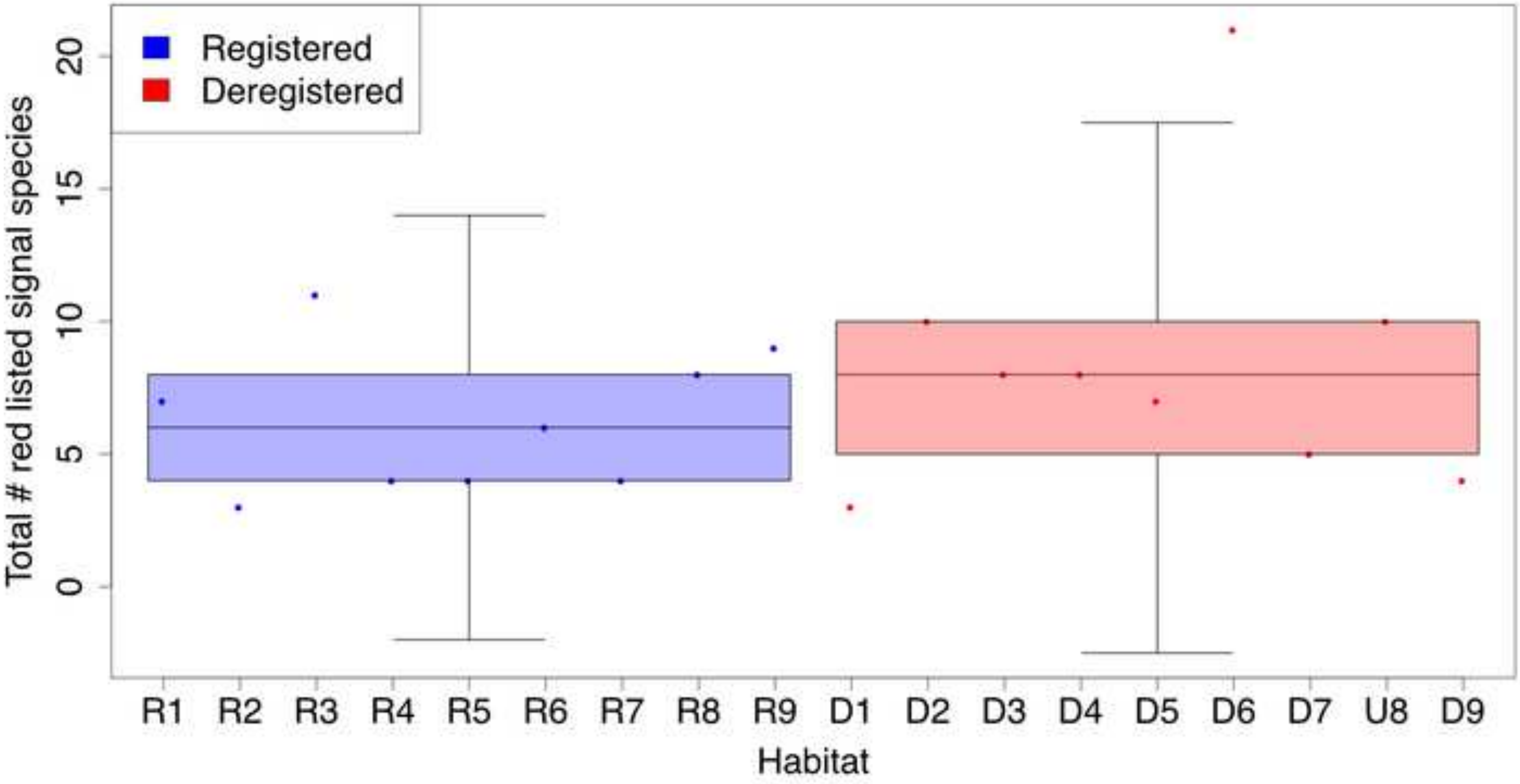

Click here to access/download;Figure;figure 4.tiff

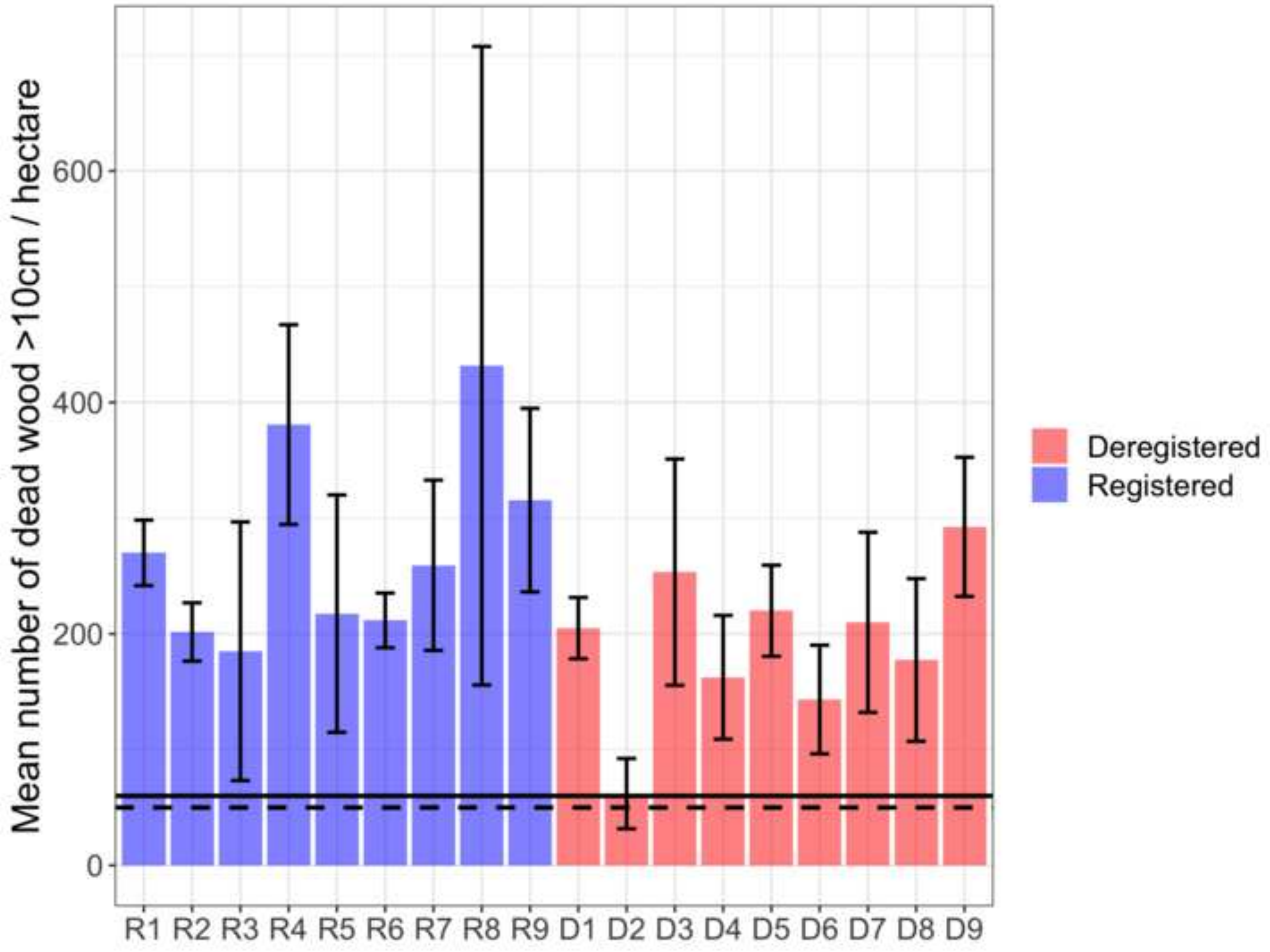

Figure

Click here to access/download;Figure;figure 5.tiff

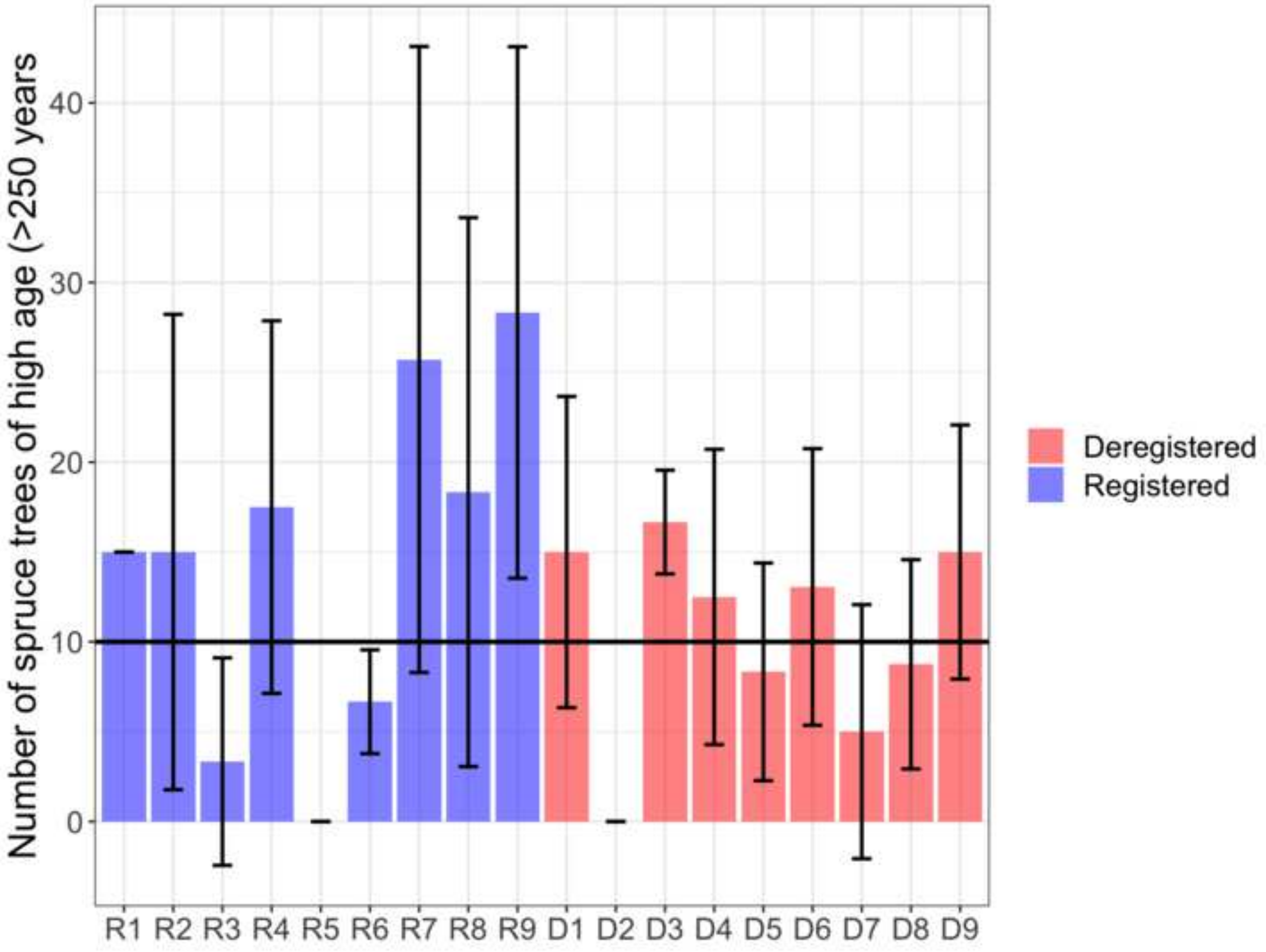

Figure

Click here to access/download;Figure;figure 6.tiff

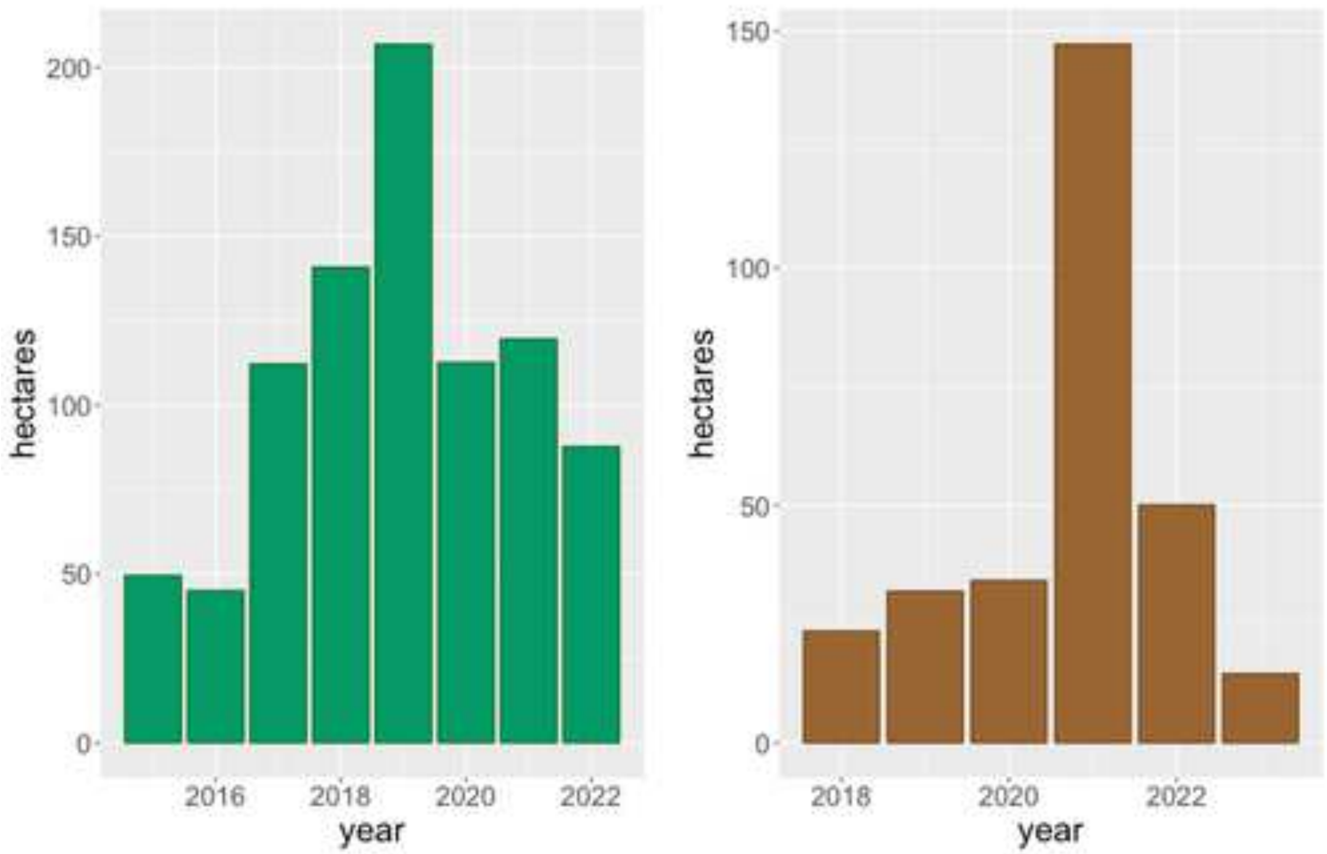

Figure
Click here to access/download;Figure;figure 7.tiff

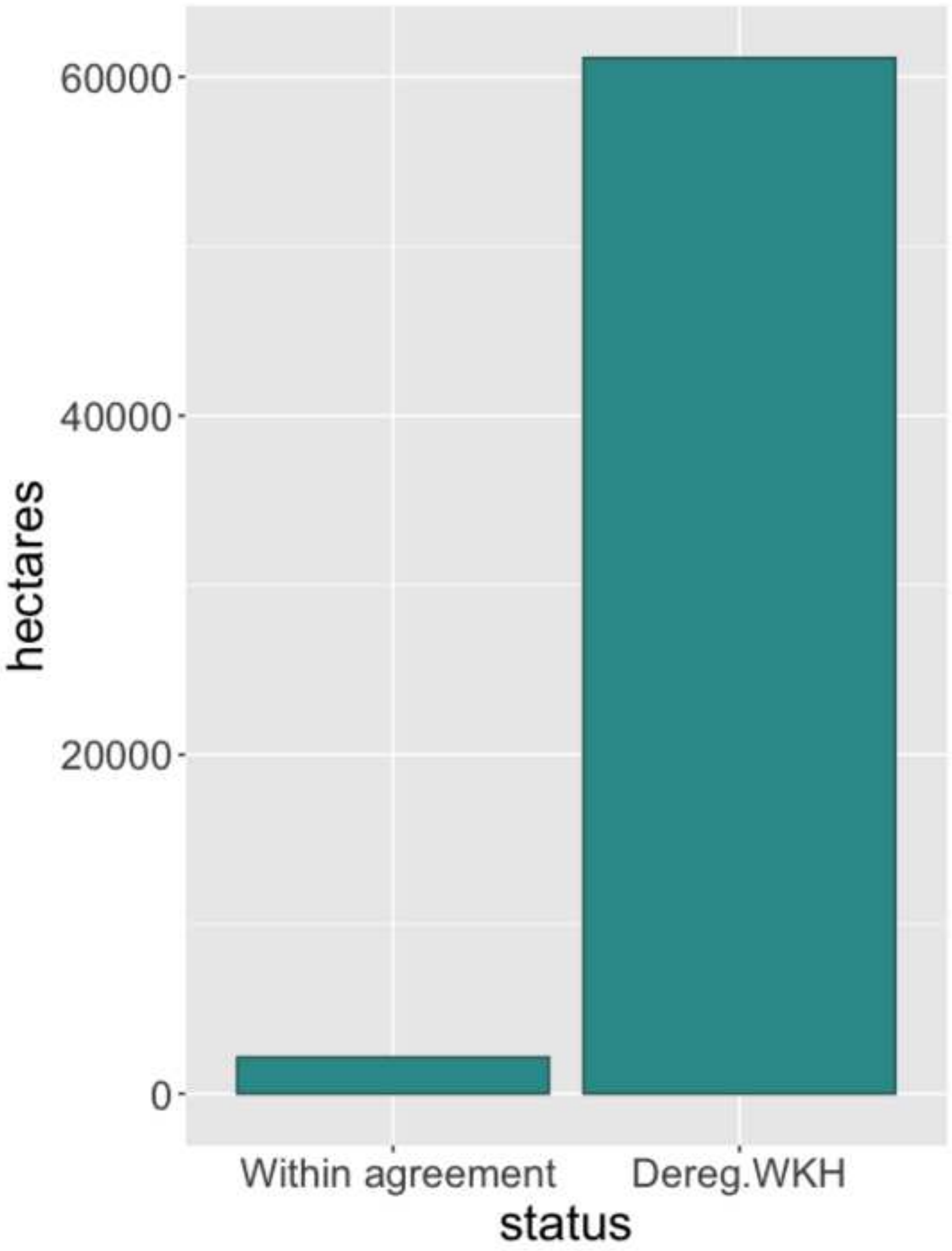

Supplement: Attachment — Submitted filename: PONE-D-24-09128.pdf [file pone.0331191.s003.pdf]
