# Supplementary material for: Status and future prospect of deregistered woodland key habitats in Northwestern Sweden
Source: PLoS One. 2025 Oct 8;20(10):e0331191. doi: 10.1371/journal.pone.0331191 (PMC12507246; doi:10.1371/journal.pone.0331191)
Supplement: Attachment — Submitted filename: PONE-D-24-09128_R1_reviewer.pdf [file pone.0331191.s005.pdf]

PLOS ONE

Status and future prospect of deregistered woodland key habitats in northwestern Sweden

--Manuscript Draft--

Manuscript Number:	PONE-D-24-09128R1
Article Type:	Research Article
Full Title:	Status and future prospect of deregistered woodland key habitats in<br><br>northwestern Sweden
Short Title:	Status and future prospect of deregistered woodland key habitats in northwestern Sweden
Corresponding Author:	ulrika Ervander<br>University of Gothenburg: Goteborgs Universitet<br>gothenburg, SWEDEN
Keywords:	boreal forest; biodiversity; Conservation; old growth forests; inventories
Abstract:	Extensive primary boreal forests within Europe are primarily located in northwestern Sweden, Finland and Russia. These forests host numerous endemic and red-listed species but are exploited rapidly. Over the past thirty years, certain highly valuable areas within Swedish forests have been designated as “woodland key habitats” (WKH) and safeguarded from clear-cutting within certified forests. Despite their high value, WKHs lack proper legislative protection and recently many WKHs were “deregistered”. In this study we conducted a field inventory following a novel inventory method proposed by the Swedish forest Agency, featuring more stringent criteria for classification of WKH in north western Sweden. The inventory was conducted in 9 registered and 9 recently deregistered WKHs to asses their conservation values. Our inventory results shows that all 18 areas reach the criterias in the new method, despite the higher threshholds for conservation values. Analysis of recent harvest actions within deregistered key habitats in Sweden indicated than over 800 hectares of forest cutting occurred within these areas post-deregistration. The study also highlights conflicts between the new inventory method and the national goals for species protection. Since WKH harbor red-listed species in the forest landscape it is crucial to exclude these areas from forestry practices to preserve biodiversity in boreal forests.
Order of Authors:	ulrika Ervander
	Willian Arestides Ferreira Da Silva
	Karin Hårding
Response to Reviewers:	Answers to Journal requirements:<br>When submitting your revision, we need you to address these additional requirements.<br>1. Please ensure that your manuscript meets PLOS ONE's style requirements, including those for file naming. The PLOS ONE style templates can be found at https://journals.plos.org/plosone/s/file?id=wjVg/PLOSOne_formatting_sample_main_body.pdf and https://journals.plos.org/plosone/s/file?id=ba62/PLOSOne_formatting_sample_title_authors_affiliations.pdf<br>2. Please note that PLOS ONE has specific guidelines on code sharing for submissions in which author-generated code underpins the findings in the manuscript. In these cases, all author-generated code must be made available without restrictions upon publication of the work. Please review our guidelines at https://journals.plos.org/plosone/s/materials-and-software-sharing#loc-sharing-code and ensure that your code is shared in a way that follows best practice and facilitates reproducibility and reuse.<br>A: The code used is ready to be shared and will be made avaliable upon publication of the work<br>3. We note that Figure 1 in your submission contain copyrighted images. All PLOS content is published under the Creative Commons Attribution License (CC BY 4.0),

Powered by Editorial Manager® and ProduXion Manager® from Aries Systems Corporation

	which means that the manuscript, images, and Supporting Information files will be freely available online, and any third party is permitted to access, download, copy, distribute, and use these materials in any way, even commercially, with proper attribution. For more information, see our copyright guidelines: http://journals.plos.org/plosone/s/licenses-and-copyright.<br>A: The correct copyrite information has been added to the map and ledgend for Fig 1.<br><br>4. We notice that your supplementary table is included in the manuscript file. Please remove them and upload them with the file type 'Supporting Information'. Please ensure that each Supporting Information file has a legend listed in the manuscript after the references list.<br>A: The supplementary table is now removed and added to the manuscript as Table 1 since there were a lot of comments on both the method and the results which we hope can be more clear when showing the summary from the field survey in the the text.
Additional Information:	
Question	Response
**Financial Disclosure**<br><br>Enter a financial disclosure statement that describes the sources of funding for the work included in this submission. Review the submission guidelines for detailed requirements. View published research articles from *PLOS ONE* for specific examples.<br><br>This statement is required for submission and **will appear in the published article** if the submission is accepted. Please make sure it is accurate.<br><br>**Funded studies**<br>Enter a statement with the following details:<br>• Initials of the authors who received each award<br>• Grant numbers awarded to each author<br>• The full name of each funder<br>• URL of each funder website<br>• Did the sponsors or funders play any role in the study design, data collection and analysis, decision to publish, or preparation of the manuscript?<br><br>Did you receive funding for this work?	The author(s) received no specific funding for this work.
**Competing Interests**<br><br>Use the instructions below to enter a competing interest statement for this submission. On behalf of all authors, disclose any competing interests that	the authors have declared that no competing interests exist

could be perceived to bias this work—acknowledging all financial support and any other relevant financial or non-financial competing interests.<br><br>This statement is required for submission and **will appear in the published article** if the submission is accepted. Please make sure it is accurate and that any funding sources listed in your Funding Information later in the submission form are also declared in your Financial Disclosure statement.<br><br>View published research articles from *PLOS ONE* for specific examples.<br><br>**NO authors have competing interests**<br><br>Enter: *The authors have declared that no competing interests exist.*<br><br>**Authors with competing interests**<br><br>Enter competing interest details beginning with this statement:<br><br>*I have read the journal's policy and the authors of this manuscript have the following competing interests: [insert competing interests here]*<br><br>* typeset	
**Ethics Statement**<br><br>Enter an ethics statement for this submission. This statement is required if the study involved:<br><br>• Human participants<br>• Human specimens or tissue<br>• Vertebrate animals or cephalopods<br>• Vertebrate embryos or tissues<br>• Field research<br><br>Write "N/A" if the submission does not require an ethics statement.	N/A

General guidance is provided below. Consult the submission guidelines for detailed instructions. **Make sure that all information entered here is included in the Methods section of the manuscript.**<br><br>**Format for specific study types**<br><br>**Human Subject Research (involving human participants and/or tissue)**<br>• Give the name of the institutional review board or ethics committee that approved the study<br>• Include the approval number and/or a statement indicating approval of this research<br>• Indicate the form of consent obtained (written/oral) or the reason that consent was not obtained (e.g. the data were analyzed anonymously)<br><br>**Animal Research (involving vertebrate animals, embryos or tissues)**<br>• Provide the name of the Institutional Animal Care and Use Committee (IACUC) or other relevant ethics board that reviewed the study protocol, and indicate whether they approved this research or granted a formal waiver of ethical approval<br>• Include an approval number if one was obtained<br>• If the study involved *non-human primates*, add *additional details* about animal welfare and steps taken to ameliorate suffering<br>• If anesthesia, euthanasia, or any kind of animal sacrifice is part of the study, include briefly which substances and/or methods were applied<br><br>**Field Research**<br><br>Include the following details if this study involves the collection of plant, animal, or other materials from a natural setting:<br>• Field permit number<br>• Name of the institution or relevant body that granted permission	
**Data Availability**<br><br>Authors are required to make all data	Yes - all data are fully available without restriction

underlying the findings described fully available, without restriction, and from the time of publication. PLOS allows rare exceptions to address legal and ethical concerns. See the PLOS Data Policy and FAQ for detailed information.<br><br>A Data Availability Statement describing where the data can be found is required at submission. Your answers to this question constitute the Data Availability Statement and **will be published in the article**, if accepted.<br><br>**Important:** Stating 'data available on request from the author' is not sufficient. If your data are only available upon request, select 'No' for the first question and explain your exceptional situation in the text box.<br><br>Do the authors confirm that all data underlying the findings described in their manuscript are fully available without restriction?	
**Describe where the data may be found in full sentences. If you are copying our sample text, replace any instances of XXX with the appropriate details.**<br><br>• If the data are **held or will be held in a public repository**, include URLs, accession numbers or DOIs. If this information will only be available after acceptance, indicate this by ticking the box below. For example: *All XXX files are available from the XXX database (accession number(s) XXX, XXX.*).<br>• If the data are all contained **within the manuscript and/or Supporting Information files**, enter the following: *All relevant data are within the manuscript and its Supporting Information files.*<br>• If neither of these applies but you are able to provide **details of access elsewhere**, with or without limitations, please do so. For example:<br><br>*Data cannot be shared publicly because of [XXX]. Data are available from the*	"The field data collected for this study are available as Supporting Information. Table S1 includes all information on plots within each area, and Table S2 includes all collected data in field. The data used for geographical analysis in GIS on harvest and harvest reports are publicly available from Skogsstyrelsen ((2020) Map of woodland key habitats from Swedish Forest Agency)) (https://www.skogsstyrelsen.se/sjalvservice/karttjanster) and (2023) (https://www.skogsstyrelsen.se/sjalvservice/karttjanster/geodatatjanster/). The data are available directly from the website, and for this study the authors downloaded shapefiles within the date interval described in the paper. Data on deregistered key habitats were gathered by the forest group in Kronoberg within the Swedish Society for Nature Conservation (Skogsgruppen Kronobergs Naturskyddsförening (2019)). These data are available in the Supporting Information files (skogsgruppen_data_2019.shp)."

Powered by Editorial Manager® and ProduXion Manager® from Aries Systems Corporation

XXX Institutional Data Access / Ethics Committee (contact via XXX) for researchers who meet the criteria for access to confidential data.<br><br>*The data underlying the results presented in the study are available from (include the name of the third party and contact information or URL).*<br>• This text is appropriate if the data are owned by a third party and authors do not have permission to share the data.<br><br>* typeset	
Additional data availability information:	Tick here if the URLs/accession numbers/DOIs will be available only after acceptance of the manuscript for publication so that we can ensure their inclusion before publication.

Powered by Editorial Manager® and ProduXion Manager® from Aries Systems Corporation

Manuscript Click here to access/download;Manuscript;manuscript.docx

Status and future prospect of deregistered woodland key habitats in northwestern Sweden

Ulrika Ervander[1] , Willian T.A.F. Silva[1,2] and Karin C. Harding[1].

[1]Department of Biology and Environmental Sciences, University of Gothenburg, Gothenburg, Sweden
[2]*Department of Aquatic Resources, Swedish University of Agricultural Sciences, Turistgatan 5, 453 30 Lysekil, Sweden. ORCID: 0000-0002-9517-485X.*

Abstract

Extensive primary boreal forests within Europe are primarily located in northwestern Sweden, Finland and Russia. These forests host numerous endemic and red-listed species but are exploited rapidly. Over the past thirty years, certain highly valuable areas within Swedish forests have been designated as “woodland key habitats” (WKH) and safeguarded from clear-cutting within certified forests. Despite their high value, WKHs lack proper legislative protection and recently many WKHs were “deregistered”. In this study we conducted a field inventory following a novel inventory method proposed by the Swedish forest Agency, featuring more stringent criteria for classification of WKH in north western Sweden. The inventory was conducted in 9 registered and 9 recently deregistered WKHs to asses their conservation values. Our inventory results shows that all 18 areas reach the criterias in the new method, despite the higher threshholds for conservation values. Analysis of recent harvest actions within deregistered key habitats in Sweden indicated than over 800 hectares of forest cutting occurred within these areas post-deregistration. The study also highlights conflicts between the new inventory method and the national goals for species protection. Since WKH harbor red-listed species in the forest landscape it is crucial to exclude these areas from forestry practices to preserve biodiversity in boreal forests.

Keywords: boreal forest, biodiversity, conservation, old growth forests, inventories.

Commented [UE1]: Add nature conservation and old growth forest and something more?

1.Introduction

Primary forests and intact forest landscapes worldwide play a crucial role in maintaining global ecological diversity and mitigating the impacts of global warming [1]. Despite growing awareness of their ~~great~~ importance, primary forests continue to decline and become increasingly fragmented at an alarming rate [2, 3]. Approximately 44% of the intact forest landscapes globally consist of boreal forests [1]. In Europe, large intact boreal forests are now restricted to northwestern Russia and Fennoscandia [4,5]. In northwestern Sweden there are still old-growth forests that have never been clear-cut, which gives these forests a unique status in the European Union [4]. This green belt of old-growth forest sustains rich biodiversity and holds significant conservation values. Despite their unique ecological importance, the natural forests in northwestern Sweden are constantly diminishing due to high demands for wood and land for establishing new production forests [6, 3]. The rapidly changing and fragmented forest landscape in boreal Sweden has resulted in a substantial loss of both habitat area and quality of habitat for red listed species and 'signal species' [7]. Signal species are species that are indicators for forests of old age and high biodiversity values [8]. Currently there are 4127 species listed on the Swedish red list, with 2131 of these associated with the forest biotope [7]. Notably, 75% of these forest species are red-listed because of the diminishing habitat due to the deforestation of old-growth forests [7]. The conversion of continuous primary forests and old-growth forests into clear-cuts and plantations is recognized as having the most pronounced negative impact on redlisted species in a Nordic perspective and contributes significantly to the ongoing loss of biodiversity globally [9, 7, 37].

In 1990, the Swedish Forest Agency (SFA) initiated the identification of Woodland Key Habitats (WKHs) to pinpoint the most valuable areas within the Swedish forests [10, 11]. Woodland Key Habitats are defined as habitats capable of supporting red-listed species [11]. On average, more than 20 red-listed and signal species can be found in a WKH [10]. Currently, many WKHs are relatively small areas, with an average area of 4.6 hectares [12]. WKH areas are often relics in a fragmented forestlandscape and

serve as refuges for threatened species and can host source populations for future recolonization efforts [13,14]. It has been proposed that special attention should be given to forests adjacent to WKHs to enhance the long-term spread and survival of red-listed species [10]. An assessment of a recently developed method for classifying WKHs by the SFA, revealed that over 55,000 hectares of WKHs were subjected to clear-cutting between 2000 and 2018 [15]. The logging of valuable WKHs occurs despite the fact that most big forest companies in Sweden are certified according to the standards of the Forest Stewardship Council (FSC) or the Program for the Endorsement of Forest Certification (PEFC). These certifications do not allow harvesting of WKHs, however, the classification of a forest as a WKH does not confer formal legal protection [16]. Instead, it represents a classification of the forest's conservation values based on inventory results [14].

The WKH inventory method relies on an visual inventory, wherein the surveyor visits a forest identified as a likely WKH and documents the presence and frequency of structures and species associated with old growth forests. The forest history, age, tree layers and elements of high conservation value are documented, and these data serve as the basis for the classification of the area [11]. The SFA has developed checklists for northern Sweden in the old inventory method, but these have generally not been consistently and systematically applied. These checklists were primarily developed for surveyors lacking biological training or expertise in classifying a WKH. The aim of the inventory was to promptly identify areas with high nature conservation value using a straightforward and practical method. The application of these checklists was not based on systematic surveys or test areas [17]. Due to substantial regional variations in Swedish forest landscapes, spanning 4000 km from north to south, applying the same method for WKH inventories in the whole country can be problematic. The major part of the old growth forests in northwestern Sweden is within or close to the mountain region [18]. Forests in northwestern Sweden differ significantly from forests in other regions in terms of climate (lower temperatures), productivity (lower light incidence), accessibility (difficult terrain), and consequently have a history of less extensive exploitation. Because of that, the proportion of natural old-growth forests is significantly greater

Commented [UE2]: May serve as … and may host

in these regions than elsewhere in Sweden and the formally protected areas as well as the voluntary set-aside areas are larger. Registered WKHs are also more common in this region, and it is highly probable that there are numerous still unidentified WKHs and other valuable areas for biodiversity in this inadequately documented region [17].

The inventory method for woodland key habitats has remained relatively unchanged since the start of the WKH identification survey in 1990, with the same method applied across the country. However, in 2017 the Swedish Forest Agency decided to halt the WKH inventory work in northwestern Sweden [11], an area identified for having the highest density of unprotected old forests [18]. In 2021 SFA announced that they will stop the WKH inventories for the entire country, shifting the responsibility for identifying these areas to the forest owner. The decision to halt WKH inventories in 2017 was driven by the need to develop a new method that could address challenges in assessing and delimiting WKHs in a more effective way. Especially in regions with extensive areas of old forests [19]. The inventory suspension continued until January 2018, during which the new inventory method was evaluated throughout 2018 and published in 2019. The new method is intended to be more systematic with higher thresholds for an area to qualify as a woodland key habitat in northwestern Sweden [11]. The classification of an area is determined by specific criteria outlined in a checklist, which is applied within each area. The result obtained from the checklists should be used as a strong guidance in the classification of a forest, however, it is crucial to emphasize that the classification of a key habitat involves a comprehensive assessment of both the forest and the surrounding landscape. [11]. In this study we investigate the performance of the developed survey method for WKHs in northwestern Sweden and compare the conservation value of registered WKHs and recently ‘’deregistered’’ WKHs. The study highlights the potential consequences that a switch in inventory method and the ongoing deregistration of WKH may have on these valuable forest areas. If an area loses its WKH classification status, this may have consequences for the long-term protection of the area as certified forest owners are not allowed to harvest WKHs. To see how de-registration of an area may affect

the future of the forest, we also investigate the level of harvesting and harvesting reports recorded within de-registered WKHs.

2.Materials and Methods

2.1 Study area

The fieldwork was carried out in the boreal forests in northwestern Sweden close to the town of Sorsele in in the county of Västerbotten durig 2019. Within the study area, covering a 20 km radius from Sorsele (see Figure 1A), all registered and recently deregistered woodland key habitats(WKH) owned by the state-owned forest company Sveaskog AB were identified on maps obtained from the SFA. Out of the 96 identified areas initially classified as WKHs using the old method, 41 of them have been deregistered, and thus lost their status as WKHs. Among these 9 registered and 9 deregistered WKHs were randomly selected and included in the study. To minimize variations arising from company-specific inventory methods, only forests managed by the state-owned forest company, Sveaskog AB, were included.

The software QGIS (3.16.15 Hannover) was used to generate a grid of 200 m x 200 m squares on the map of the study area. The number of sample sites per forest was determined according to the SFA's guidelines for the new developed inventory method (i.e., forest areas between 0,5-10 hectares require one sample site for every 2-3 hectares while a smaller forest requires only one sample site). The specified number of sample sites were randomly distributed across the map (Figure 1). Coordinates and number of plots are presented in additional information.

2.2 Field survey

Each forest was surveyed according to the field manual for the new method for identifying WKHs in northwestern Sweden [11]. The filed manual includes checklist of parameters, adjusted according to what kind of forest it is (spruce dominate or pine dominated etc.). All checklists include the following parameters: conservation trees of different species per hectare, lying and standing dead wood of different

species per hectare, continuity of tree age, continuity of dead wood, number of old trees, presence of red listed and signal species and other descriptors specific to the surveyed forest type [15]. The term “conservation tree” is defined in the method by SFA as a tree with significant importance for biodiversity such as really old trees (>250 years), trees with hanging lichens or those with holes for birds, for example. Trees that are likely to develop properties that have great importance for biodiversity are also included within the term “conservation tree”. Species are recorded by area during the inventory and is included in the list regardless of whether they are present within a sample plot or not. All signal and red-listed species are included in the overall assessment of the key biotope [15].

A forest is defined as a likely WKH if a certain number of criteria specific to forests with high conservation values are met. There are two different routes to reach the status of WKH (track 1 and Track 2): in track 1 a few high-value criteria suffice, while in track 2 a longer list of lower-value criteria is required. For example, in track 1, having 30 conservation spruce trees per hectare is adequate to reach one of the six criteria, whereas in track 2, 20 conservation spruce trees per hectare is required to reach one of the 22 criteria. In northwestern Sweden, a surveyed forest is designated as a WKH if it satisfies five out of the six criteria in track 1. The number of criteria in track 2 that must be fulfilled depends on the location as well as size of the forest: forests smaller than 20 hectares need to fulfill at least nine criteria, whereas larger forests need to fulfill at least eight criteria. All checklist and full description of the method used for the survey in this study are included in the report by SFA [15].

2.3 Statistical and geographical analyses

To asses statistical differences in number of conservation trees, old trees and dead wood between registered and deregistered forests, a linear mixed-effects model was used, accounting for the nested structure of the data (multiple plots within habitats). The model included registration (registered vs. deregistered) as a fixed effect and habitat as a random effect. For looking at the number of redlisted and signal species between the two groups registered and deregistered forests a a generalized linear

model, was fitted to the data. The total number of ~~speceis~~ served as the response variable, while registration status and habitat were considered as independent variables. The statistical significance of the effect of the independent variables on the response variable was tested using an analysis of deviance with the Chi-square test. All statistical analyses were conducted using the software R and R-studio (Version 2022.12.0+353). To study the frequency of harvesting in these areas, QGIS was used to analyse maps of conducted harvesting from the Swedish forest agency together with maps of deregistered WKHs. Geographical analysis was performed using QGIS (3.16.15 – Hannover) and the geoprocessing tool intersection. Geographical data of harvesting operations carreid out within deregistered key habitats after the deregistration date was combined and the intersecting areas was calculated. The data of harvesting operations from SFA spans from 2010-01-01 to 2023-03-02, but only data of harvesting operations from dates after deregisration of key habitats included in this study was analyzed [25].

3. Results

3.1 Testing the new inventory method

The results of testing the new inventory method on forests included in this study confirm that all surveyed forests fulfill the criteria for WKHs according to the new method for northwestern Sweden. Forest D2 (located on Abmoberget) did not fulfill the criteria in boths tracks within the method, but still met the criteria in track 2, which is sufficient for WKH classification (see Table 1).

Table 1 : Summery of results from the field survey

ID	Criteria track 1	Criteria track 2	deregistered (year)	Class- At time of survey	Survey result	Red-listed and signal species (classification from red list)
R1	6/6	18/22		WKH	WKH	phlebia mellea(VU), Dryocopus martius(NT), Lobaria pulmonaria(NT), Lobaria scrobiculata(NT), Alectoria sarmentosa(NT), phellinus chrysoloma(NT), Nephroma bellum(LC).
R2	6/6	15/22		WKH	WKH	phellinus chrysoloma (NT), Alectoria sarmentosa (NT), fomitopsis rosea(NT)
R3	5/6	16/22		WKH	WKH	Haploporus odorus (VU), Diplomitoporus lenis (VU), Antrodia albobrunnea (VU), Alectoria sarmentosa (NT), Lobaria pulmonaria (NT), Dryocopus martius (NT), phellinus chrysoloma (NT), Lobaria scrobiculatus (NT), Nephroma bellum(LC), Nephroma resupinatum (LC), Icmadophila ericetorum(LC).
R4	6/6	15/22		WKH	WKH	Alectoria sarmentosa (NT), phellinus chrysoloma (NT), Picoides tridactylus(NT), pseudographis pinicola (NT)
R5	6/6	12/22		WKH	WKH	Alectoria sarmentosa (NT), phellinus chrysoloma (NT), Lobaria scrobiculata(NT), Icmadophila ericetorum(LC).
R6	5/6	14/22		WKH	WKH	Alectoria sarmentosa (NT), Dryocopus martius (NT), Cystostereum murrayi(NT), Phellinus chrysoloma (NT), Lobaria scrobiculatus (NT), Nephroma resupinatum (LC).
R7	6/6	16/22		WKH	WKH	Phellinus chrysoloma (NT), Alectoria sarmentosa (NT), Perisoreus infaustus(LC), Matteuccia struthiopteris(LC).
R8	6/6	18/22		WKH	WKH	Fomitopsis rosea (NT), Leptoporus mollis(NT), Picoides tridactylus(NT), Alectoria sarmentosa (NT), Pseudographis pinicola (NT), phellinus chrysoloma (NT), Perisoreus infaustus (LC), Nephroma bellum(LC).
R9	6/6	16/22		WKH	WKH	Cystostereum murrayi(NT), Alectoria sarmentosa (NT), phellinus chrysoloma (NT), Onnia leporina(NT), Perisoreus infaustus(LC), Icmadophila ericetorum (LC), Aconitum lycoctonum (LC), Icmadophila ericetorum (LC), Nephroma resupinatum (LC).
D1	6/6	16/22	2015	promise	WKH	Alectoria sarmentosa (NT), phellinus chrysoloma (NT), Icmadophila ericetorum (LC).
D2	2/6	12/22	2017	production	WKH	Haploporus odorus (VU), skeletocutis odora (VU), Phellinidium ferrugineofuscum (NT), Carbonicola anthracophila (NT) , Dryocopus martius (NT), pseudographis pinicola (NT), Alectoria sarmentosa (NT), Phellopilus nigrolimitatus (NT), Onnia leporina (NT), Nephroma resupinatum (LC).
D3	6/6	18/22	2015	promise	WKH	Poecile cinctus (VU), Alectoria sarmentosa (NT), phellinus chrysoloma (NT), Onnia leporina(NT),Picoides tridactylus (NT) Nephroma bellum (LC), Nephroma resupinatum (LC), Meruliopsis taxicola (LC).
D4	6/6	18/22	2015	promise	WKH	Skeletocutis odora(VU), fomitopsis rosea (NT), phellinus chrysoloma (NT), Alectoria sarmentosa (NT), Phellinidium ferrugineofuscus (NT), Leptoporus mollis (NT), Perisoreus infaustus(LC), Icmadophila ericetorum (LC).
D5	6/6	15/22	2015	promise	WKH	Alectoria sarmentosa (NT), Phellinidium ferrugineofuscus,(NT) fomitopsis rosea (NT), Onnia leporina(NT), phellinus chrysoloma (NT), Phellopilus nigrolimitatus (NT), Icmadophila ericetorum (LC).
D6	5/6	18/22	2015	promise	WKH	Antrodia albobrunnea (VU), Diplomitoporus lenis (VU), Skeletocutis odora(VU), Phlebia mellea(VU), Laurilia sulcata(VU), Onnia leporina (NT), Alectoria sarmentosa (NT), phellinus chrysoloma (NT), pseudographis pinicola (NT), Cystostereum murrayi (NT), Leptoporus mollis (NT), Phellinidium ferrugineofuscum (NT), Lobaria pulmonaria (NT), fomitopsis rosea (NT), Phellopilus nigrolimitatus (NT), Picoides tridactylus (NT), Hypogymnia bitteri (NT), Icmadophila ericetorum (LC), Meruliopsis taxicola(LC), Perisoreus infaustus (LC), Nephroma bellum(LC).
D7	6/6	13/22	2015	promise	WKH	Phellinus ferrugineofuscus (NT), Alectoria sarmentosa (NT), Onnia leporina (NT), fomitopsis rosea (NT), Nephroma bellum(LC).
D8	6/6	16/22	2015	promise	WKH	Laurilia sulcata (VU), Phellinidium ferrugineofuscum (NT), fomitopsis rosea (NT), Cystostereum murrayi (NT), phellinus chrysoloma (NT), Phellopilus nigrolimitatus (NT), Alectoria sarmentosa (NT), Picoides tridactylus (NT), Icmadophila ericetorum(LC), Perisoreus infaustus (LC),
D9	6/6	15/22	2015	promise	WKH	Alectoria sarmentosa (NT), phellinus chrysoloma (NT), pseudographis pinicola (NT), Nephroma bellum(LC)

Table 1: Survey result of all 18 forests randomly selected from the study area. Criteria track 1 - forest likely a WKH if more than 5 criteria are fulfilled. Criteria track 2 - forest likely a WKH if more than eight or nine criteria are fulfilled (depending on the size and surrounding). Results of all findings of red-listed and signal species from field survey.

As mentioned in the introduction the classification of a key habitat is a comprehensive assessment of both the forest and the surrounding landscape. The map in Figure 1 illustrates that forest D2 is part of a green infrastructure from a landscape perspective. The new method favors large areas and areas in proximity to other WKHs. According to the developed method, a so-called “50/50 rule” can be applied in a continuous forest landscape characterized by high conservation values, especially when borders are hard to delimit [11]. This rule dictates that if 50% of the area reaches the criteria for WKH standard, the entire area should be designated as a WKH. The area of classified WKH in the sourrounding forest landscape cover over 450 hectares and the two deregistered forests cover together less than 1/4th of the size of the surrounding WKH. The 50/50-method thus strengthens the assessment to classify his forest as a WKH (see Figure 1).

The classification of the deregistered WKHs at the time of the field survey is also presented in Table 1. Forest D2 is classified as a production forest and the remaining eight deregistered forests were re-labeled as “non-exchangeable promises”. A “non-exchangeable promise” is a category of voluntary set-aside area with a higher protection from exploitation through forestry than a regular set-aside area [20]. The protection of these areas relies on an agreement between Sveaskog and the county board [21]. During 2023, however, these 8 ”non-exchangeable promises” areas were again reclassified and are now part of a long term protection. Of the more than 61000 hectares of deregistered WKH included in the map of deregistered WKH included in this study, 2149 hectares were included in this new agreement for long-term protection of valuable forests in the mountain region [22, 23]

Fig 1. This is the Fig 1 Title: Map of study area.

This is the Fig 1 legend.

Figure 1: The star in map A denotes the location of the study area in northwestern Sweden, with red dots illustrating the distribution of all deregistered WKH included in the map used in this study [23] . Map B in a closeup of the study area, where all WKHs are indicated in blue and deregistered WKH are indicated with red, all surveyed areas are marked with a label. Map C presents deregistered WKH D2 (now classified as production forest), in a landscape perspective. Boundaries of forests of high conservation value according to the County Administrative Board are delineated by a white line. [23, 24, 25, Basemap from OpenStreetMap https://www.openstreetmap.org/]

3.2 Conservation values in registered and deregistered WKH.

A majority of the interior of the surveyed forests displayed no clear indications of human disturbance and had clearly never undergone large-scale exploitation. The biodiversity and conservation value of all forests were notably high, all having more than 100 conservation trees per hectare and many red-listed species. The forests are old, dominated by spruce, with a high amount of dead wood and many old living trees with an age over 250 years and draped with pendulous lichens. The number of red-listed and signal species in the surveyed forests varied from 3 to 21 with an average of 7.4 species per WKH. Full list of species is presented in Table 1. The largest deregistered WKH (D6) had the highest number of observed red-listed species, with 18 species belonging to the Swedish red list, of which 5 classified as vulnerable (VU). In the deregistered WKH, D2, currently classified as production forest, nine red-listed species were identified, including two VU species.

3.3 Comparison between findings in studied WKH and established criteria in new inventory method

Important quantifiable parameters within the method are the amount of conservation trees, amount of dead wood, the presence of old trees and important species. All surveyed forests exceeded the set criteria of a minimum of 20 conservation trees per hectare for track 1. Additionally, the number of spruce conservation trees was also above the criteria in track 1 for all forests, with at least 30 conservation trees per hectare. The total amount of dead wood (>10cm) exceeded the set criteria of a minimum of 60 dead trees per hectare in track 1 for all areas, except for WKH D2. As illustrated in Figure 4, current production forest D2 had a relatively low amount of dead wood per hectare. The analysis of number of conservation trees per hectare showed a marginally significant difference between the two groups (see Figure 2). The p-value is low enough to assume that registered key habitats host a larger number of trees compared to unregistered key habitats ($p=0.0531$). The random effect for habitat indicated significant variability in conservation tree counts between habitats (variance = 3024). No significant difference was found

between the two categories of forests when looking at the difference in number of signal and red-listed species ($p < 0.08$), see Figure 3.

Fig 2. This is the Fig 2 Title: Conservation trees per hectare**.**

This is the Fig 2 legend.

Figure 2: Variation of the mean number of conservation trees per hectare within each forest. The small box plots show the variation within each forest and the large box plots show the variation within the categories registered WKH (R) and deregistered WKH (D). The significance of the effect of registration status on the mean number of conservation trees per habitat was p=0.0531. Red line shows criteria for track 1 by the new inventory method, i.e., 20 conservation trees per hectare. It can be noted that all forests are well above the limit of 20 trees per hectare set as a criteria for WKH.

Fig 3. This is the Fig 3 Title: number of red-listed and signal species**.**

This is the Fig 3 legend.

Figure 3: Variation of the number of red-listed and signal species observed during survey within each forest. The box plots show the variation within the categories registered WKH (R1-9) and deregistered WKH (D1-9). The Chi-square test between the two groups registered and unregistered WKH shows no significant difference in the index of number of red-listed and signal species ($p < 0.08$).

The total amount of dead wood and living trees of high age in the studied forests was high compared to the criteria in the inventory method. The total average amount of dead wood (both standing and lying) in all areas was 192 per hectare and is well above the citerias in the method, 50/ hectare in track one and 60/ hectare in track two (See Figure 4) . The average amount of dead wood for each individual area was well above the critaria except for area D2 where the average amount within the area was 62 per hectare an just met the criteria. A linear mixed model with habitat as a random effect revealed a significant difference in total deadwood between registered and deregistered key biotopes. Registered key biotopes had higher amount of total deadwood than deregistered ones ($p = 0.000831$). Habitat explained a significant portion of the variation in total deadwood, with a variance of 1133 (SD = 33.67), while residual variance was 1934 (SD = 43.98). These findings suggest that registration status significantly influences deadwood levels, with habitat-specific variation also playing a role.

Fig 4. This is the Fig 4 Title: Average number of dead wood**.**

This is the Fig 4 legend.

Figure 4: The average total number of dead wood objects per hectare, both standing and lying and standard deviation (SD) with registered showed in purple and deregistered showed in red. The dead wood criteria are 50/hectare in track one (dashed line)and 60/hectare in track two (solid line).

The number of living spruce trees of old age (>250 years) per hectare is shown in Figure 5, 11 of the 18 forests passed, with an average of more than 10 spruce of old age per hectare, however this variable had a high variation between the sample sites.The analysis found no significant difference in the number of old spruce trees between registered and deregistered key biotopes ($p = 0.154$). The random effect for habitat indicated variability in the number of old spruce trees across habitats (variance = 36.36).

Fig 5. This is the Fig 5 Title: Number of living spruce trees of high age **.**

This is the Fig 5 legend.

Figure 5: Number of living spruce trees of high age >250 years per hectare and standard deviation (SD) in the randomly assigned sample plot in studied forests. Criteria for track 2 (black line) in the developed method is 10 trees per hectare.

3.4 Harvest actions within deregistered key habitats since deregistration

The deregistered WKH included in this study underwent deregistration between 2015 and 2018, before the SFA stopped to intentify and register WKH in 2021. Combining the database of harvesting reports with the maps of deregistered WKH shows that harvesting has been conducted within 874 hectares of deregistered WKH and 301 hectares were reported for harvesting after deregistration (Figure 6).

Fig 6. This is the Fig 6 Title: harvested forest and forest reported for cutting per year**.**

This is the Fig 6 legend.

Figure 6
To the left harvested forest in hectares per year within the deregistered key habitats in Sweden. To the right Forest reported for cutting in hectares per year within the deregistered key habitats in Sweden.

4. Discussion

SAMMANFATTA RESULTAT

Based on the analysis of the field survey, we conclude that all 18 surveyed registered and deregistered WKHs in this study meet the criteria to be classified as WKH under the new method. These areas represent remnants of old forest in a fragmented forest landscape, typically surrounded by mires, plantations and clear-cuts. The surveyed forests showed high numbers for many of the core qualities within the method like the number of dead wood, conservation trees, trees of high age and important species. Signal and red-listed species were present in all forests, and in six of them species classified as vulnerable (VU) on the red list were found. One of the deregistered WKHs has recently been reclassified as a production forest, while the remaining deregistered WKHs have been incorporated into a permanent protection plan for valuable mountain forests [22]. However, this protection plan does not include most of the deregistered WKHs across Sweden. The deregistration of WKH poses a potential threat to the conservation of valuable forest areas as these habitats are indirectly protected within certified forest management. There can be various reasons behind a deregistration, for example to register the area as another form of nature conservation or to register the area as a production forest. The results of this study shows that logging has been conducted within many deregistered WKH after deregistration.

4.1 Current classification of deregistered WKH in Västerbotten

Eight of the deregistered forests included in the study are now part of a new agreement for long-term protection of mountain forests. This agreement between the Swedish Environmental Protection Agency and Sveaskog, signed in February 2023, protects nearly 100, 000 hectares of mountain forests, including 48,000 hectares of productive forestland and 50,000 hectares of low productive forestland [22]. Of the 61 000 hectares of deregistered key habitats only 2100 hectares were included in this agreement. The deregistered WKH D2 were deregistration as WKH and registered as production forests, despite having high conservation values according to both this study and previous field surveys [26, 27]. The amount of dead wood and old spruce trees were lower in D2, contributing to this forest not reaching the requirements in track 1. However, there were signs of a previous forest fire in this forest, which can diminish the amount

of dead wood. On the other hand, such fires are known to correlate with other biodiversity values. Area D2 fulfill the criteria for being a likely WKH in track 2 and is also a part of a green infrastructure directly connected to other WKHs. This should allow the application of the 50/50 rule, an addition to the new method, which states that if there are uncertainties about classification and 50% of the area is of WKH standard, then the entire area should be registered as a WKH [11]. The consequence of deregister D2 as a WKH is that this old-growth forest with unique biodiversity values, contributing to a green infrastructure by connecting other segments of old growth forests, can now be subjected to clear-cutting with only six weeks of prior notice according to the current Swedish forestry laws [28]. According to previous surveys conducted by the Swedish Society for Nature Conservation and by the County Administrative Board in Västerbotten in 2003, D2 (Located in Abmoberget) should qualify as a WKH [26,27]. The survey report from the County Administrative Board in Västerbotten states that the forest at Abmoberget (see Figure 1) is a large continuous area with very high nature conservation values and any clear signs of forestry are absent throughout the area [27]. The oldest investigated spruce in the entire area reached the age of 497 years [26]. The County Administrative Board argues that the forest landscape on Abmoberget is of high nature conservation value and can be severely damaged by forestry and other exploitation [27]. In forest D2, nine red-listed species were found during this field study and surveys of the greater Abmoberget area found 13 red-listed species and 11 signal species [27]. The varying conclusions regarding Abmoberget highlight the importance of correctly classifying and delimiting a forest and how a deregistration of a WKH might lead to loss of voulnerable forest areas.

4.2 Evaluating the developed inventory method

The new method is well structured and, to some extent, aligns with the objectives of transparency, objectivity and predictability. However, certain aspects of the method remain inevitably subjective and dependent on the surveyor. The identification of redlisted and signal species, places a lot of responsibility on the surveyor and introduces subjectivity to the method. To address this subjectivity, an independent

observer could conduct the survey. The new method was in part developed to (better) consider regional differences, however, it does not consider one crucial regional difference: snow. An unfortunate oversight, as the method is impossible to conduct properly without clear visibility of the forest floor, typical effect of witer conditions across northern Sweden. The forests in the inland of Västerbotten, for example, might be covered by snow up to five months of the year, which stands in contrast to the current rule of a six week prior notice before clear cutting. If this period of notice would be extended, regional weather aspects could be better considered. A longer period between reporting and harvest could provide more time for inspections, which could help prevent the fragmentation of these valuable forests. The ongoing fragmentation of forests is a major driver of ecosystem degradation and biodiversity loss [29,30]. The new method includes guidelines that favor large WKHs and those situated near other WKHs and has the potential to enhance the creation of a less fragmented forest landscape, promoting larger areas of old-growth forests that are less vulnerable to species extinction. One consequence with a method with stricter critera, in an area with higher proportion of old growth forests and many potentially unregistered WKH, may be that valuable forests may be at risk of falling below the criteria of the new method that would have been included by the old method.

4.3 The significance of WKH and the future of WKH inventory

The concept of WKH was not created as a protection form but a classification of the quality of the forest [17]. The concept was turned into an indirect protection form by sustainable forest companies who use the WKH concept in their criteria. In the standard for both the Forest Stewardship Council (FSC) and the Program for the Endorsement of Forest Certification (PEFC), there are regulations for certified companies to avoid cutting and selling wood from WKHs [16, 31]. In this way the WKH concept indirectly implies a protection status for all certified companies, as a market-driven commitment to save them. The challenge with employing WKH as a protection form lies in the fact that the WKH concept was not originally designed to function as a protected area. Consequently, the WKH concept lacks the inclusion of buffer zones.

Another issue arises from the concept's primary focus on identifying hotspots in the landscape, while to ensure a sustainable future for the forest landscape and the species that are depending on it, it is necessary to protect a larger continuous green infrastructure including these hotspots [32,33]. To ensure that these valuable areas of old-growth forest won't be harvested and transformed into production forests we need a common classification and avaliable open information. Without access to information we cannot see the full picture and are unable to follow the ongoing actions within these forests, unfortunately we see a shift towards less avaliable access to information about WHK. Since SFA stopped register WKH, a complete map of WKH in Sweden is no longer available since many companies now only show them in their own system, making it harder to get an overview of ongoing changes. The long term system of finding and registering valuable woodland key habitats occurred between 1990 – 2019. During 2021 it was decided that SFA no longer should identify and register WKHs, resulting in already registered WKH being deregistered since the SFA no longer had the authority to register these as from June 2019.

4.4 The role of Swedish woodland Key habitats in a global perspective

Deregistration of WKH and higher citeria for classification of WKH in the northwestern part of Sweden, were we find the highest portiotion of old-growth forests does not align with national and international goals for biodiversity conservation and nature protection. By the year 2030 the EU biodiversity strategy sets out a commitment to protect at least 30 % of areas of the land, to enhance biodiversity and ecosystem functions.[34]. At leat one third should be under strict protection and include all remaining primary and old-growth forests [34]. The focus on high yeild wood production during a long period has resulted in loss of natural and diverse forests in many European regions while the forests of north western Sweden still have areas of unique old-growth forest left [5]. Despite local and global goals for restoring terrestrial ecosystems, old growth forests are being harvested at an alarming rate in Sweden [3]. Considering the uinque value of these forests there is a need for more inspections before cutting near WKHs. Proactive measures and a strict system of controls before harvest are essential to ensure that forests with significant nature conservation values are not replaced by clear-cuts followed by

planted production forests. Protected areas around the world lost 4.1% of their forested area between 2001 and 2018 which also highligt the importance to keep control over deforestation of valuable forest areas[36].

5. Conclusion

The quality of the studied registered and deregistered woodland key habitats (WKHs) in Västerbotten County is high, with a low level of human disturbance. Despite the unique values of forests in northwestern Sweden, the developed method for WKH inventory have higher cirteria for WKHs this area. In addition to regional adaptations in the new method we propose implementing a notice period of at least six snow-free weeks before harvesting a forest. A recommended course of action is to grant the Swedish Forest Agency the authority to conduct surveys of all old growth forests before any cutting or thinning activities take place. There is a also need of a standardized classification system among forest companies, along with open access to information regarding classification of old growth forest areas, ensuring a transparent and secure approach to harvesting old growth forest in Sweden. Deregistered key habitats, with high conservation values, can still be turned into production forests after deregistration, which means that it is important to conduct more controls within these forests to verify the legitimacy of a deregistration and prevent the loss of valuable ecosystems. As emphasized by the SFA, there are most likely many still unidentified WKHs. These areas must be identified and protected to ensure that Woodland key habitats can persist as refuges for threatened species in the future. The green belt of old growth forests in northwestern Sweden still has the potential to support high biodiversity and should be excluded from forestry to meet the European biodiversity strategy.

Acknowledgments.

We thank Gudrun Norstedt, Frank Götmark and Tero Härkönen for assisting with design of the field study as well as useful comments on the manuscript. Jonas Nordenström is thanked for helping out with species identification and support in the field. Also thanks to Swedish society for nature conservation and the local forest group of Kronoberg for the work behind the map of deregistered key habitats.

References

1. Potapov P., Yaroshenko A., Turubanova S., Dubinin M., Laestadius L., Thies C., Aksenov D., Egorov A., Yesipova Y., Glushkov I., Karpachevskiy M., Kostikova A., Manisha A., Tsybikova E., Zhuravleva I. 2008. Mapping the World's Intact Forest Landscapes by Remote Sensing. Ecology and Society, 13 (2) Map of Intact forest landscapes.

2. Heino, M.; Kummu, M.; Makkonen, M.; Mulligan, M.; Verburg, P.H.; Jalava, M.; Räsänen, T.A. 2015, Forest loss in protected areas and intact forest landscapes: A global analysis. PLoS ONE 2015,10: e0138918.http://www.intactforests.org/data.ifl.html Retrieved 2020-05-01.

3. Ahlström, A., Canadell, J. G., & Metcalfe, D. B. (2022). Widespread unquantified conversion of old boreal forests to plantations. Earth's Future, 10, e2022EF003221. https://doi.org/10.1029/2022EF003221

4. Jonsson, B., Svensson, J. Mikusiński, Grzegorz, Manton, Michael, Angelstam, P.. (2019). European Union's Last Intact Forest Landscapes are at A Value Chain Crossroad between Multiple Use and Intensified Wood Production. Forests. 10. 564. 10.3390/f10070564.

5. Sabatini, F. et al. (2018). Where are Europe's last primary forests?. Diversity and Distributions. 10.1111/ddi.12778.

6. Svensson, J. et al. (2018). Landscape trajectory of natural boreal forest loss as an impediment to green infrastructure. Conservation Biology. 33. 10.1111/cobi.13148.

7. ArtDatabanken (2015). "Rödlistade arter i Sverige 2015."

8. Skogsstyrelsen (2023) Skogsstyrelsens signalarter – en komplett förteckning, Version 2023-1, https://www.skogsstyrelsen.se/globalassets/miljo-och-klimat/nyckelbitoper/skogsstyrelsens-signalarter---version2023-1.pdf retrieved 2024-11-19

9. Larsson, A. (red) 2011 Tillståndet i skogen – rödlistade arter i ett nordiskt perspektiv. ArtDatabanken Rapporterar 9. ArtDatabanken SLU, Uppsala

10. Nitare, J. (2019). Skyddsvärd skog Naturvårdsarter och andra kriterier för naturvärdesbedömning. Stibo Graphics, Skogsstyrelsen Jönköping.

11. Skogsstyrelsen (2019,a). "Handbok för nyckelbiotopsinventering." https://www.skogsstyrelsen.se/globalassets/miljo-och-klimat/nyckelbitoper/handbok-nyckelbiotopsinventering.pdf

12. Gustafsson, L., Hannerz, M. 2018. 20 års forskning om nyckelbiotoper – Här är resultaten. Institutionen för ekologi, Sveriges lantbruksuniversitet (SLU), Uppsala. 134 pp.

13. Djupström, L. B., Weslien, J., Schroeder, L. M. (2008). Dead wood and saproxylic beetles in set-aside and non set-aside forests in a boreal region. Forest Ecology and Management, 255: 3340-3350.

14. Skogsstyrelsen. (2019,b, 2019-07-02). "vårt arbete med nyckelbiotoper."

15. Skogsstyrelsen (2019,d), utveckling av metod för nyckelbiotopsinventering i nordvästra Sverige. https://www.skogsstyrelsen.se/globalassets/om-oss/rapporter/rapporter-20222021202020192018/rapport-2019-12-utveckling-av-metod-for-nyckelbiotopsinventering-i-nordvastra-sverige.pdf

16. FSC 2010 Swedish FSC Standard for Forest Certification including SLIMF indicators FSC-STD-SWE-02-02-2010 Sweden Natural, Plantations and SLIMF EN FSC. 2019. https://se.fsc.org/se-se/klagoml/offentliga-certifierarrapporter retrieved 2019-12-11

17. Skogsstyrelsen (2016). "Nulägesbeskrivning om nyckelbiotoper." rapport 7 2016. https://cdn.abicart.com/shop/9098/art33/84164433-105ca2-Nyckelbiotoper_webb.pdf

18. Skogsdata (2018), Aktuella uppgifter om de svenska skogarna från Riksskogstaxeringen. SLU, Sveriges officiella Statistik, Umeå.

19. Claesson, S. (2018). Nulägesbeskrivning av nordvästra Sverige. kunskapsunderlag. Skogsstyrelsen.

20. Skogsindustrierna, (2019). https://www.skogsindustrierna.se/skogsindustrin/hallbarhet/hallbartskogsbruk/frivilligt-avsatt-skog/karta/ retrieved 2019-11-15

21. Naturvårdsverket. 2015. .Skyddsvärda statliga skogar - Kategorisering av områden på Sveaskogs markinnehav i Västerbottens län. publikation 5497.

22. Naturvårdsverket 2023. Fjällskog - överenskommelse om långsiktigt skydd https://www.naturvardsverket.se/om-oss/aktuellt/nyheter-och-pressmeddelanden/2023/februari/vardefulla-fjallskogar-skyddas-i-ny-overenskommelse/fjallskogar--overenskommelse-langsiktigt-skydd/ 2023-05-20

23. Skogsgruppen 2019, Naturskyddsföreningen Kronoberg, work by Johan Marand and Erland Lindblad, https://www.skogsgrupp.se/bolagens-borttagna-nb/ retrieved 2019-06-20

24. Skogsstyrelsen (2020) Map of woodland key habitats from Swedish Forest Agency https://www.skogsstyrelsen.se/sjalvservice/karttjanster/ retrieved 2020-01-20.

25. Skogsstyrelsen (2023), https://www.skogsstyrelsen.se/sjalvservice/karttjanster/geodatatjanster/ Retrieved: 2023-03-02

26. Nygren P., Eilertssen L. 2015, Naturskyddsföreningen Västerbottenslän - Abmoberget https://docplayer.se/156084859-Text-patrik-nygren-forutom-avsnitt-6-6-och-6-7-som-skrivits-av-lilleilertsen. html retrieved 2019-12-01

27. Länsstyrelsen Västerbotten 2017, Skogar med höga naturvärden i Västerbottens län - Länsstyrelsens skogsinventeringar nedan gränsen för fjällnära skog 2003–2015 https://www.lansstyrelsen.se/download/18.304d30c1612954524550dee/1526067883061/2017-09-15_Skogar%20med%20h%C3%B6ga%20naturv%C3%A4rden%20i%20V%C3%A4sterbottens%20l%C 3%A4n.pdf

28. Skogsstyrelsen (2022), Skogsvårdslagstiftningen gällande regler september 2022, https://www.skogsstyrelsen.se/globalassets/lag-och-tillsyn/skogsvardslagen/skogsvardslagstiftningen-2022.pdf

29. Pfeifer, M., Lefebvre, V., Peres, C. et al. Creation of forest edges has a global impact on forest vertebrates. Nature 551, 187–191 (2017). https://doi.org/10.1038/nature24457

30. Undin, M., Atrena, A., Carlsson, F. et al. To what extent does surrounding landscape explain stand-level occurrence of conservation-relevant species in fragmented boreal and hemi-boreal forest? – a systematic review. Environ Evid 13, 19 (2024). https://doi.org/10.1186/s13750-024-00346-1

31. PEFC, 2024 https://pefc.se/vara-standarder/svenska-pefc-standarden/pefc-om-nyckelbiotoper

32. Ericsson, Tysk & Berglund, Håkan & Östlund, Lars. (2005). History and biodiversity of woodland key habitats in south boreal Sweden. Biological Conservation. 122. 289-303. 10.1016/j.biocon.2004.07.019.

33. Rybicki, Joel & Hanski, Ilkka. (2013). Species-area relationships and extinctions caused by habitat loss and fragmentation. Ecology letters. 16. 10.1111/ele.12065.

34. European Parliament, Council of the European Union (2024), Regulation (EU) 2024/1991 of the European Parliament and of the Council of 24 June 2024 on nature restoration and amending Regulation (EU) 2022/869 (Text with EEA relevance), ELI: http://data.europa.eu/eli/reg/2024/1991/oj

35. OpenStreetMap, (2021), Basemap from OpenStreetMap https://www.openstreetmap.org/

36. Wade, C. M., Austin, K. G., Cajka, J., Lapidus, D., Everett, K. H., Galperin, D., Maynard, R., & Sobel, A. (2020). What Is Threatening Forests in Protected Areas? A Global Assessment of Deforestation in Protected Areas, 2001–2018. Forests, 11(5), 539. https://doi.org/10.3390/f11050539

37. FAO and UNEP. 2020. The State of the World's Forests 2020. Forests, biodiversity and people. Rome, https://doi.org/10.4060/ca8642en

Supporting information

1. S2. Table. Coordinates of plots . table S1 Show the coordinates and number of plots per for all surveyed areas.
2. S3 Table. Results from field survey. Table S2 show the full result from the field survey.

Figure 1

Click here to access/download;Figure;Figure 1.tiff

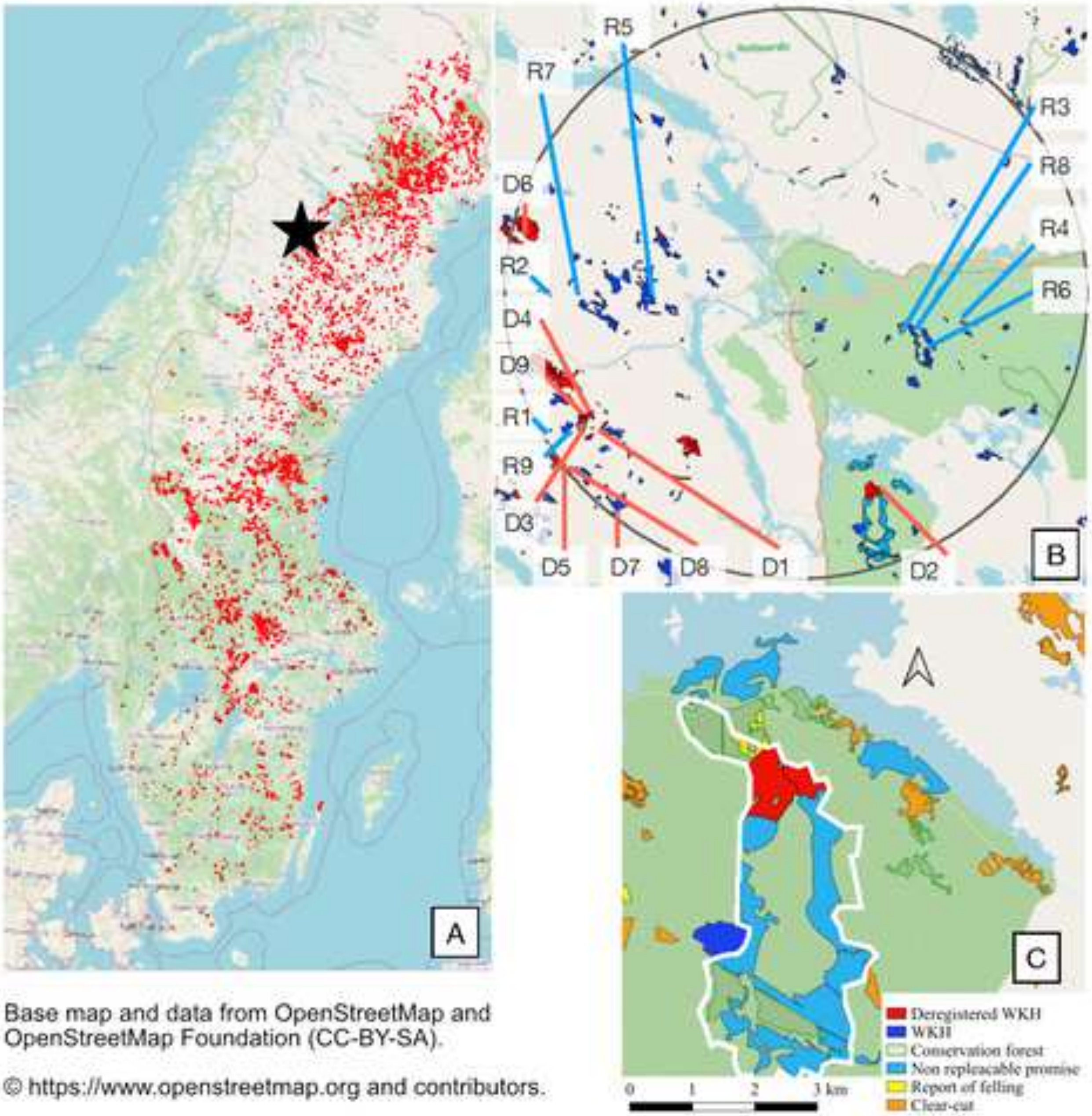

Base map and data from OpenStreetMap and OpenStreetMap Foundation (CC-BY-SA).

© https://www.openstreetmap.org and contributors.

Figure 6 Click here to access/download;Figure;Figure 6.tiff

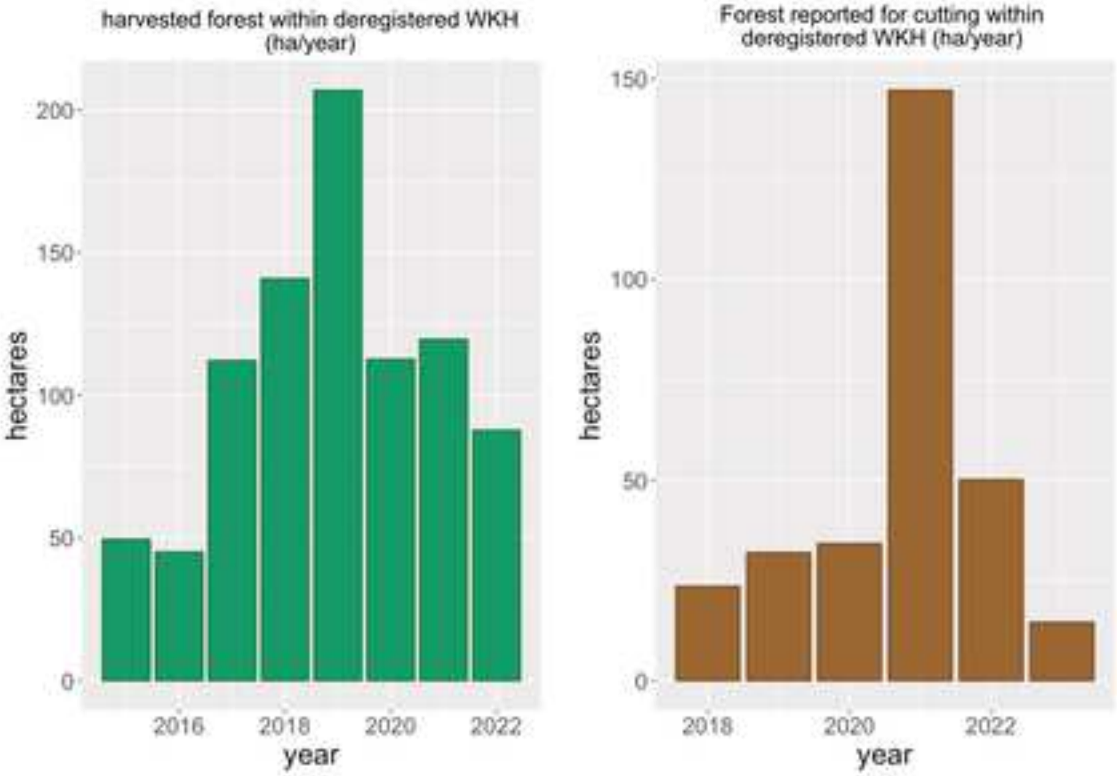

Figure 2

Click here to access/download;Figure;figure 2.tiff

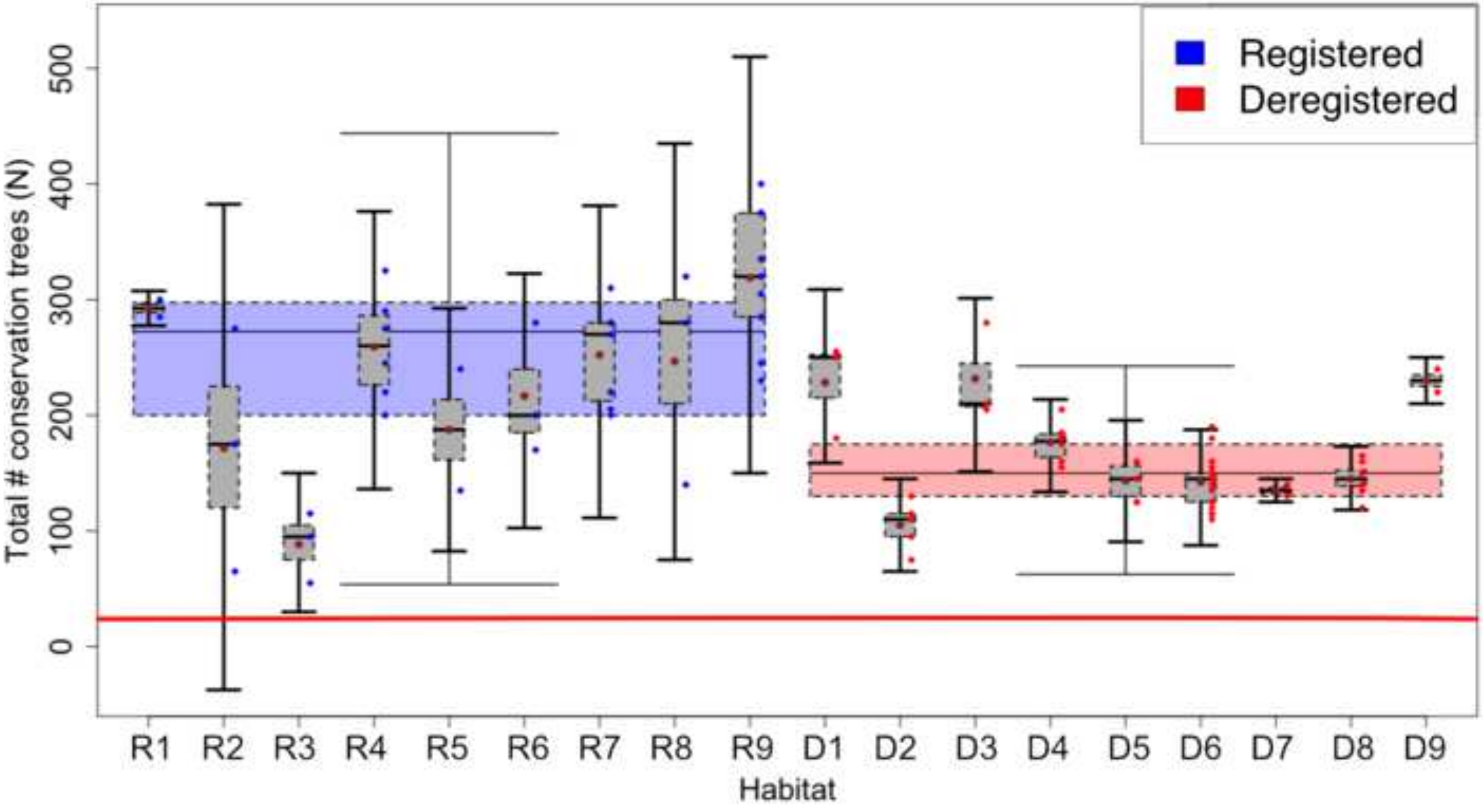

Figure 3

Click here to access/download;Figure;figure 3.tiff

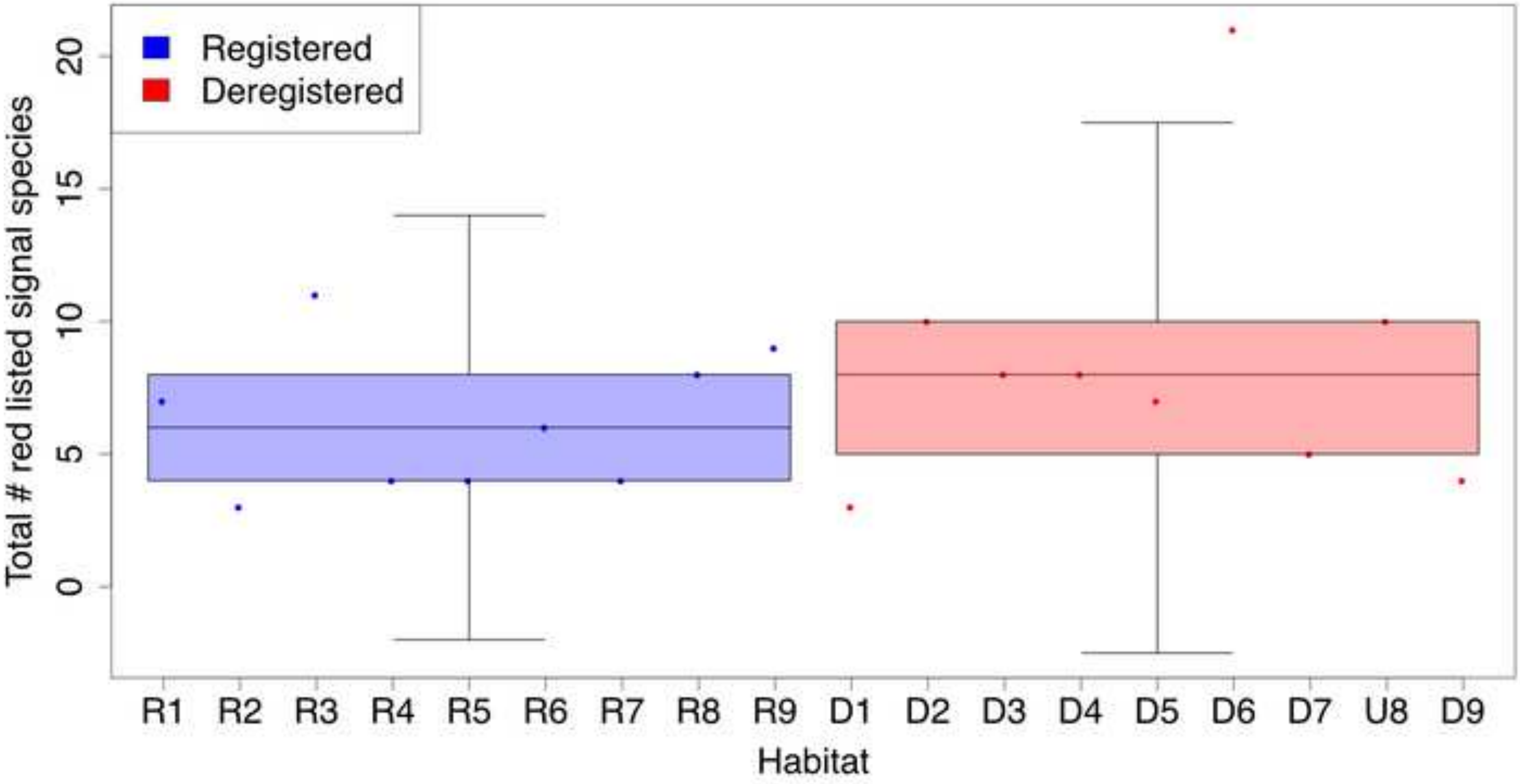

Figure 4

Click here to access/download;Figure;figure 4.tiff

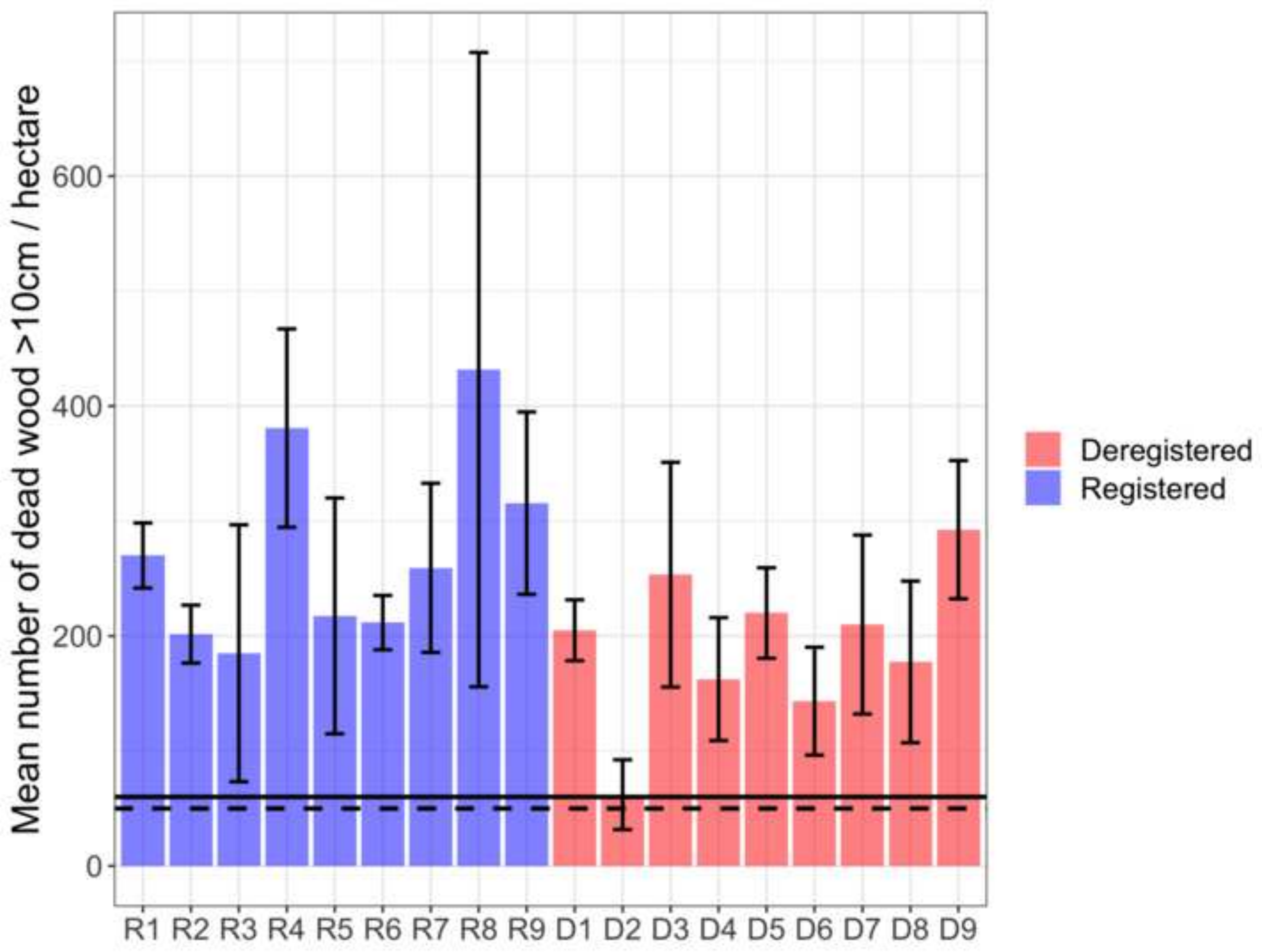

Figure 5

Click here to access/download;Figure;figure 5.tiff

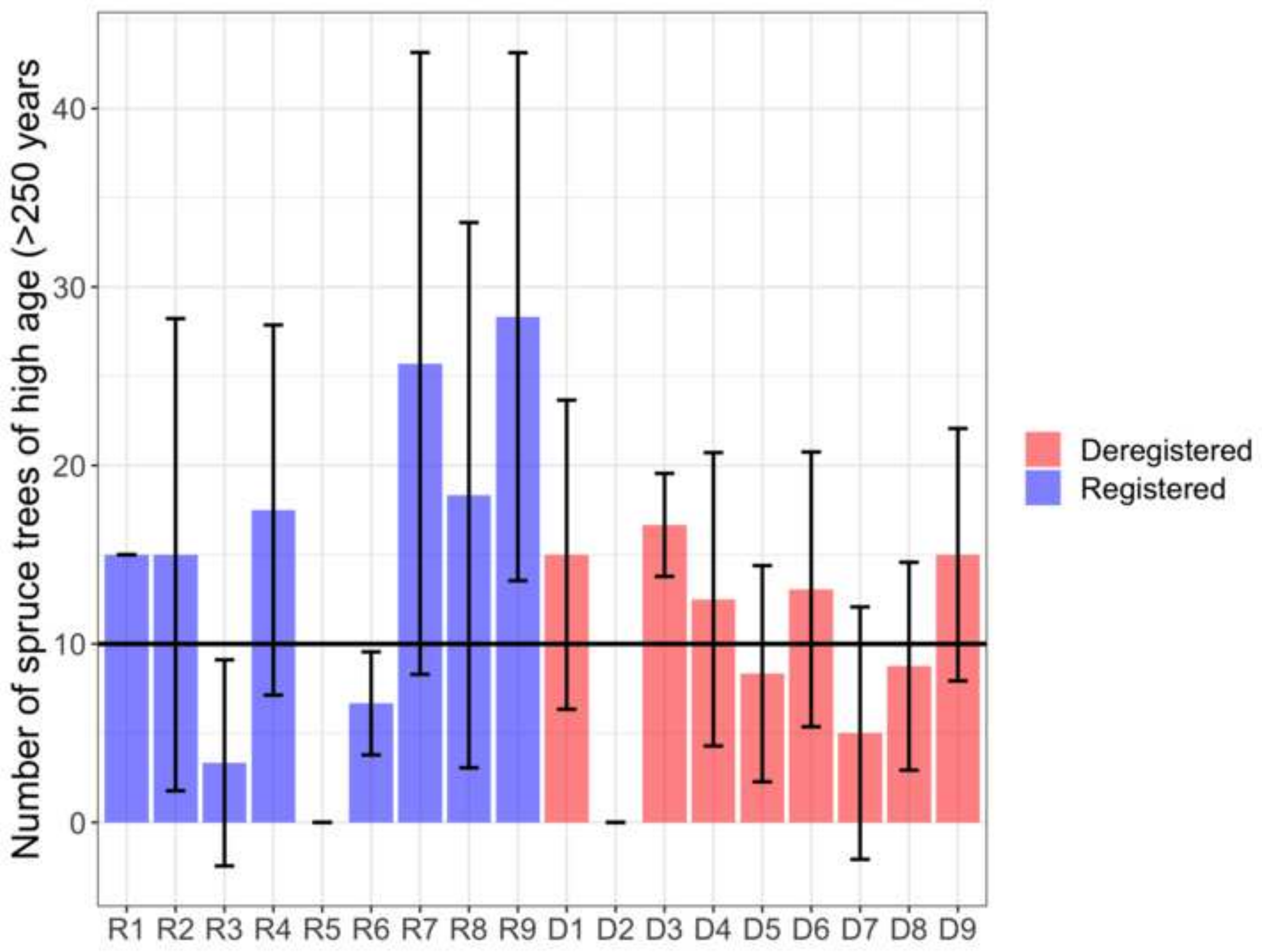

Supporting Information table S1

Click here to access/download
Supporting Information
Table S1 - Supporting info.xlsx

Supporting Information table S2

Click here to access/download
Supporting Information
Table S2- Supporting info.xlsx

Click here to access/download
Supporting Information
skogsgruppen_data_2019.shp

data availability statement

Click here to access/download
Supporting Information
Data avaliability statement.docx

Status and future prospect of deregistered woodland key habitats in northwestern Sweden

Ulrika Ervander[1] , Willian T.A.F. Silva[1,2] and Karin C. Harding[1].

[1]Department of Biology and Environmental Sciences, University of Gothenburg, Gothenburg, Sweden
[2]*Department of Aquatic Resources, Swedish University of Agricultural Sciences, Turistgatan 5, 453 30 Lysekil, Sweden. ORCID: 0000-0002-9517-485X.*

Abstract~~BSTRACT~~

Extensive primary boreal forests within ~~the~~ Europe~~an Union~~ are primarily located in northwestern Sweden, ~~and~~ Finland and Russia~~d~~. These forests host numerous endemic and red-listed species but are exploited rapidly. Over the past thirty years, certain highly valuable areas within Swedish forests have been designated as “woodland key habitats” (WKH) and safeguarded from clear-cutting within certified forests. Despite their high value, WKHs lack proper legislative protection and recently many WKHs were “deregistered~~classified~~”. In this study we conducted a field inventory following a novel inventory method proposed by the Swedish forest Agency, featuring more stringent criteria for classification of WKH in north western Sweden. The inventory was conducted in ~~of~~ 9 registered and 9 recently der~~-r~~egistered WKHs to asses their conservation values. ~~Additionally, we tested a novel inventory method proposed by the Swedish forest Agency, featuring more stringent criteria.~~ Our inventory results ~~revealed~~ shows that all 18 areas ~~were~~ reach the criterias~~classified as WKH using~~in the new method, despite the ~~its~~ higher threshholds for conservation values~~criteria~~. Analysis of recent harvest actions within deregistered key habitats in Sweden indicated than over 800 hectares of forest cutting occurred within these areas post-deregistration. The study also highlights conflicts between the new inventory method and the national goals for species protection. Since WKH harbor red-listed species in the forest landscape it is crucial to exclude these areas from forestry practices to preserve biodiversity in boreal forests.

Keywords: ~~woodland key habitat,~~ boreal forest, ~~deregistered woodland key habitats,~~ biodiversity, conservation, old growth forests, inventories~~.~~

Formatted: Font: 18 pt

Formatted: Font: 18 pt

Commented [UE1]: Add nature conservation and old growth forest and something more?

1.INTRODUCTIONIntroduction

Primary forests and expansive intact forest landscapes worldwide play a crucial role in maintaining global ecological diversity and mitigating the impacts of global warming [1]. Despite growing awareness of their great importance, primary forests continue to decline and become increasingly fragmented at an alarming rate [(Potapov et al. 2008, 2Heino et al. 2015, 3]). Approximately 44% of the intact forest landscapes globally consist of boreal forests [(Potapov et al. 20081]). In Europe, large intact boreal forests are now restricted to northwestern Russia and Fennoscandia [4(Jonsson 2019, Sabatini 2019, Hansen 2013)5]. In northwestern Sweden there are still old-growth forests that have never been clear-cut, which gives these forests a unique status in the European Union [(4Jonsson et al. 2019]). This green belt of old-growth forest sustains rich biodiversity and holds significant conservation values. Despite their unique ecological importance, the natural forests in northwestern Sweden are constantly diminishing due to high demands for wood and land for establishing new production forests [(6Svensson et al. 2018, Ahlström et al 20223]). The rapidly changing and fragmented forest landscape in boreal Sweden has resulted in a substantial loss of both habitat area and quality of habitat for threatened red listed species and 'signal species' [7]. Signal species are, i.e., species that are indicators for forests of old age and high biodiversity values [8].. Currently there are 4127 species listed on the Swedish red list, with 2131 of these associated with the forest biotope [7](Artdatabanken 2015). Notably, 75% of these forest species are red-listed because of the diminishing habitat due to the deforestation of old-growth forests [7]. (Artdatabanken 2015). The conversion of continuous primary forests and old-growth forests into clear-cuts and plantations is recognized as having the most pronounced negative impact on redlisted species in a Nordic perspective and contributes significantly to the ongoing loss of biodiversity globally [(Larsson et al. 20119, 7, 37Artdatabanken 2015]).

Formatted: Font: 18 pt

Formatted: Font: 18 pt

Formatted: Font: 12 pt

In 1990, the Swedish Forest Agency (-SFASkogsstyrelsen) initiated the identification of Woodland Key Habitats (WKHs) to pinpoint the most valuable areas within the Swedish forests [10(Nitare 2019, 11]Skogsstyrelsen 2019,a). Woodland Key Habitats are defined as habitats capable of supporting red-listed species [11](Skogsstyrelsen 2019,a). On average, more than 20 red-listed and signal species can be found in a WKH [10](Nitare 2019). Currently, many WKHs are relatively small areas, with an average area of 4.,6 hectares [12](Gustafsson et al. 2018). WKH areas are often , relics in a fragmented forestlandscape and surrounded by production forests and clear-cut areas. WKHs serve as refuges for threatened species and can host source populations for future recolonization efforts [13,14](Djupström 2008). It has been proposed that special attention should be given to forests adjacent to WKHs to enhance the long-term spread and survival of red-listed species [10](Nitare 2019). An assessment of a recently developed method for classifying WKHs by the SFA, revealed that over 55,000 hectares of WKHs were subjected to clear-cutting between 2000 and 2018 [15](Skogsstyrelsen 2019,d). The logging of of old-growth forestsvaluable WKHs occurs despite the fact that all most main big forest companies in Sweden are certified according to the standards of the Forest Stewardship Council (FSC) or the Program for the Endorsement of Forest Certification (PEFC). These certifications do not allow harvesting of WKHs, (Skogsstyrelsen 2019,d), however, the classification of a forest as a WKH does not confer formal legal protection, [16]. Instead, it represents a classification of the forest's conservation values based on inventory results [(14]Skogsstyrelsen 2019,b).

Commented [UE2]: May serve as … and may host

Formatted: Not Highlight

Formatted: Not Highlight

The WKH inventory method relies on an ocular visual inventory, wherein the surveyor visits a forest identified as a likely WKH on a map and documents the presence and frequency of structures and species associated with old growth forests. The forest history, age, tree layers and elements of high conservation value are documented, and these data serve as the basis for the classification of the area [11](Skogsstyrelsen 2019a).

The SFA has developed checklists for northern Sweden in the old inventory method, but these have generally not been consistently and systematically applied. These checklists were primarily developed for surveyors lacking biological training or expertise in classifying a WKH. The aim of the inventory was to promptly identify areas with high nature conservation value using a straightforward and practical method. The application of these checklists was not based on systematic surveys or test areas [17]~~(Skogsstyrelsen 2016)~~. Due to substantial regional variations in Swedish forest landscapes, spanning 4000 km from north to south, applying the same method for WKH inventories in the whole country can be~~is~~ problematic. The major part of the old growth forests in northwestern Sweden is within or close to the mountain region [18]. Forests in northwestern Sweden differ significantly from forests in other regions in terms of climate (lower temperatures), productivity (lower light incidence), accessibility (difficult terrain), and consequently have a history of less extensive exploitation. Because of that, the proportion of natural old-growth forests is significantly greater in ~~those~~ these regions than elsewhere in Sweden and the formally protected areas as well as the voluntary set-aside areas are larger. ~~-~~Registered WKHs are also more common in this region, and it is highly probable that there are numerous still unidentified WKHs and other valuable areas for biodiversity in this inadequately documented region [17]~~(Skogsstyrelsen 2016)~~.

The inventory method for woodland key habitats has remained relatively unchanged since the start of the WKH identification survey in 1990~~in 1990~~, with the same method applied across the country. However, in 2017 the Swedish Forest Agency decided to halt the WKH inventory work in northwestern Sweden [11]~~(Skogsstyrelsen 2019,a)~~, an area ~~identifeid~~identified for having the highest density of unprotected old forests [~~(SLU & Sveriges officiella statistik 20~~18]~~)~~. In 2021 SFA announced that they will stop the WKH inventories for the entire country, shifting the responsibility for identifying these areas to the forest owner.

Formatted: Not Highlight

The decision to halt WKH inventories in 2017 was ~~motivated~~ driven by the need to develop a new method ~~capable~~ that could ~~of addressing~~address challenges in assessing and delimiting WKHs in a more effective way. ~~,~~ Especially~~particularly~~ in regions with extensive areas of old forests [19]~~(Claesson 2018)~~. The inventory ~~standstill~~ suspension ~~persisted~~ continued until January 2018, ~~with~~ during which the new inventory method ~~being~~ was evaluated ~~during~~ throughout 2018 and published in 2019. The new method is intended to be more systematic with higher thresholds for an area to qualify as a woodland key habitat in northwestern Sweden [11]~~(Skogsstyrelsen 2019,a)~~. The classification of an area is determined by specific criteria outlined in a checklist, which is applied within each area. The result obtained from the checklists should be used as a strong guidance in the classification of a forest, however, it is~~'s~~ crucial to emphasize that the classification of a key habitat involves a comprehensive assessment of both the forest and the surrounding landscape. [11]~~(Skogsstyrelsen 2019a)~~. In this study w~~W~~e investigate the performance of the developed survey method for WKHs in northwestern Sweden and compare the conservation value of registered WKHs and recently ''deregistered'' WKHs. The study highlights the potential consequences that a switch in inventory method and the ongoing deregistration of WKH may have on these valuable forest areas~~biodiversity~~. If an area loses its WKH classification status, this may have consequences for the long-term protection of the area as certified forest owners are not allowed to harvest WKHs. To see how de-registration of an area may affect the future of the forest, we also investigate the level of harvesting and harvesting reports recorded within de-registered WKHs.

2. Materials and Methods~~ATERIALS AND METHODS~~

2.1 Study area

Formatted: Not Highlight

Formatted: Not Highlight

Formatted: Not Highlight

Formatted: Font: 18 pt

Formatted: Font: 18 pt

Formatted: Font: 18 pt

Formatted: Font: 16 pt

The fieldwork was carried out in the boreal forests in northwestern Sweden close to the town of Sorsele in in the county of Västerbotten durig 2019. Within the study area, covering a 20 km radius from Sorsele (see Figure 1A), all registered and recently deregistered woodland key habitats(WKH) owned by the state-owned forest company Sveaskog AB were identified on maps obtained from the SFA. Out of the 96 identified areas initially classified as WKHs using the old method, 41 of them have been designated as "deregistered", and thus lost their status as WKHs. Among these 9 registered and 9 deregistered WKHs were randomly selected and included in the study. To minimize variations arising from company-specific inventory methods, only forests managed by the state-owned forest company, Sveaskog AB, were included.

The software QGIS (3.16.15 Hannover) was used to generate a grid of 200 m x 200 m squares on the map of the study area. The number of sample sites per forest was determined according to the SFA's guidelines for the new developed inventory method (i.e., forest areas between 0,5-10 hectares require one sample site for every 2-3 hectares while a smaller forest requires only one sample site). The specified number of sample sites were randomly distributed across the map (Figure 1 and Table S1). Coordinates and number of plots are presented in additional information.

2.2 Field survey

Each forest was surveyed according to the field manual for the new method for identifying WKHs in northwestern Sweden [11]. The filed manual includes checklist of parameters, adjusted according to what kind of forest it is (spruce dominate or pine dominated etc.). All checklists includes various the following parameters: conservation trees of different species per hectare, lying and standing dead wood of different species per hectare, continuity of tree age, continuity of dead wood, factors, such as the density of standing and lying dead wood, the density of trees with conservation value, the number of old spruce trees, presence of red listed and signal specieshanging lichens and about 30 other descriptors specific to the surveyed forest type [15]. escriptors (Figure S1). Red-listed species were also recorded, although it's important to note that

Formatted: Font: 16 pt

Formatted: Highlight

~~the method is not designed as a systematic survey of all red-listed species but rather offers a subsample.~~ The term "conservation tree" is defined in the method by SFA as a tree with significant importance for biodiversity such as really old trees (>250 years), trees with hanging lichens or those with holes for birds, for example. Trees that are likely to develop properties that have great importance for biodiversity are also included within the term "conservation tree". Species are recorded by area during the inventory and is included in the list regardless of whether they are present within a sample plot or not. All signal and red-listed species are included in the overall assessment of the key biotope [15].

A forest is defined as a likely WKH if a certain number of criteria specific to forests with high conservation values are met. There are two different routes to reach the status of WKH (track 1 and Track 2): in track 1 a few high-value criteria suffice, while in track 2 a longer list of lower-value criteria is required. For example, in track 1, having 30 conservation spruce trees per hectare is adequate to reach one of the six criteria, whereas in track 2, 20 conservation spruce trees per hectare is required to reach one of the 22 criteria. In northwestern Sweden, a surveyed forest is designated as a WKH if it satisfies five out of the six criteria in track 1. The number of criteria in track 2 that must be fulfilled depends on the location as well as size of the forest: forests smaller than 20 hectares need to fulfill at least nine criteria, whereas larger forests need to fulfill at least eight criteria. All checklist and full description of the method used for the survey in this study are included in the report by SFA [15].

2.3 Statistical and geographical analyses

To asses statistical ~~the potential~~ differences in ~~the~~ number of conservation trees, old trees and dead wood between registered and deregistered forests, a linear mixed-effects model was used, accounting for the nested structure of the data (multiple plots within habitats). The model included registration (registered vs.

Formatted: Font: 16 pt

Formatted: Font: 16 pt

Formatted: Font: Arial Narrow, 12 pt

deregistered) as a fixed effect and habitat as a random effect. a generalized linear model, assuming a Poisson distribution, was fitted to the data. The total number of conservation trees served as the response variable, while registration status and habitat were considered as independent variables. The statistical significance of the effect of the independent variables on the response variable was tested using an analysis of deviance with the Chi-square test. Similarly, using the same test For looking at the number of redlisted and signal species between the two groups registered and deregistered forests a a generalized linear model, was fitted to the data. The total number of speceis served as the response variable, while registration status and habitat were considered as independent variables. The statistical significance of the effect of the independent variables on the response variable was tested using an analysis of deviance with the Chi-square test. s, we examined whether the total number of red-listed and signal species depended on registration status. All statistical analyses were conducted using the software R and R-studio (Version 2022.12.0+353). To study the frequency of harvesting in these areas, QGIS was used to analyse maps of conducted harvesting from the Swedish forest agency together with maps of deregistered WKHs.

Geographical analysis was performed using the QGIS (3.16.15 – Hannover) and the QGIS geoprocessing tool intersection. to combine Ggeographical data for of various harvesting operations carreid out actions conducted within deregistered key habitats after the date of deregistration date was combined and the intersecting areas was calculated. The data of harvesting operations from SFA spans from 2010-01-01 to 2023-03-02, but only data of harvesting operations from dates after deregisration of key habitats included in this study was analyzed [25].

Formatted: Not Highlight

3. ResultsESULTS

Formatted: Font: 18 pt, Bold

Formatted: Font: 18 pt, Bold

3.1 Testing the new inventory method

Formatted: Font: 16 pt

The results from of testing the new inventory method on forests included in this study confirms that all surveyed forests would be classified asfulfill the criteria for WKHs according to the new method for

northwestern Sweden. ~~Thus, all currently registered and deregistered WKHs in our study meets the criteria for classification as WKHs under both the old and the new method.~~ Forest D2 (located on Abmoberget) ~~was the only exception that~~ did not fulfill the criteria in boths tracks within the method, but still met the criteria in track 2, which is sufficient for WKH classification (see Table 1~~S1 in Appendix for detailed results and scores from inventories.~~).

Table 1 : Summery of results from the field survey

ID	Criteria track 1	Criteria track 2	deregistered (year)	Class-At time of survey	Survey result	Red-listed and signal species (classification from red list)
R1	6/6	18/22		WKH	WKH	phlebia mellea(VU), Dryocopus martius(NT), Lobaria pulmonaria(NT), Lobaria scrobiculata(NT), Alectoria sarmentosa(NT), phellinus chrysoloma(NT), Nephroma bellum(LC).
R2	6/6	15/22		WKH	WKH	phellinus chrysoloma (NT), Alectoria sarmentosa (NT), fomitopsis rosea(NT)
R3	5/6	16/22		WKH	WKH	Haploporus odorus (VU), Diplomitoporus lenis (VU), Antrodia albobrunnea (VU), Alectoria sarmentosa (NT), Lobaria pulmonaria (NT), Dryocopus martius (NT), phellinus chrysoloma (NT), Lobaria scrobiculatus (NT), Nephroma bellum(LC), Nephroma resupinatum (LC), Icmadophila ericetorum(LC).
R4	6/6	15/22		WKH	WKH	Alectoria sarmentosa (NT), phellinus chrysoloma (NT), Picoides tridactylus(NT), pseudographis pinicola (NT)
R5	6/6	12/22		WKH	WKH	Alectoria sarmentosa (NT), phellinus chrysoloma (NT), Lobaria scrobiculata(NT), Icmadophila ericetorum(LC).
R6	5/6	14/22		WKH	WKH	Alectoria sarmentosa (NT), Dryocopus martius (NT), Cystostereum murrayi(NT), Phellinus chrysoloma (NT), Lobaria scrobiculatus (NT), Nephroma resupinatum (LC).
R7	6/6	16/22		WKH	WKH	Phellinus chrysoloma (NT), Alectoria sarmentosa (NT), Perisoreus infaustus(LC), Matteuccia struthiopteris(LC).
R8	6/6	18/22		WKH	WKH	Fomitopsis rosea (NT), Leptoporus mollis(NT), Picoides tridactylus(NT), Alectoria sarmentosa (NT), Pseudographis pinicola (NT), phellinus chrysoloma (NT), Perisoreus infaustus (LC), Nephroma bellum(LC).
R9	6/6	16/22		WKH	WKH	Cystostereum murrayi(NT), Alectoria sarmentosa (NT), phellinus chrysoloma (NT), Onnia leporina(NT), Perisoreus infaustus(LC), Icmadophila ericetorum (LC), Aconitum lycoctonum (LC), Icmadophila ericetorum (LC), Nephroma resupinatum (LC).
D1	6/6	16/22	2015	promise	WKH	Alectoria sarmentosa (NT), phellinus chrysoloma (NT), Icmadophila ericetorum (LC).
D2	2/6	12/22	2017	production	WKH	Haploporus odorus (VU), skeletocutis odora (VU), Phellinidium ferrugineofuscum (NT), Carbonicola anthracophila (NT) , Dryocopus martius (NT), pseudographis pinicola (NT), Alectoria sarmentosa (NT), Phellopilus nigrolimitatus (NT), Onnia leporina (NT), Nephroma resupinatum (LC).
D3	6/6	18/22	2015	promise	WKH	Poecile cinctus (VU), Alectoria sarmentosa (NT), phellinus chrysoloma (NT), Onnia leporina(NT),Picoides tridactylus (NT) Nephroma bellum (LC), Nephroma resupinatum (LC), Meruliopsis taxicola (LC).
D4	6/6	18/22	2015	promise	WKH	Skeletocutis odora(VU), fomitopsis rosea (NT), phellinus chrysoloma (NT), Alectoria sarmentosa (NT), Phellinidium ferrugineofuscus (NT), Leptoporus mollis (NT), Perisoreus infaustus(LC), Icmadophila ericetorum (LC).
D5	6/6	15/22	2015	promise	WKH	Alectoria sarmentosa (NT), Phellinidium ferrugineofuscus,(NT) fomitopsis rosea (NT), Onnia leporina(NT), phellinus chrysoloma (NT), Phellopilus nigrolimitatus (NT), Icmadophila ericetorum (LC).
D6	5/6	18/22	2015	promise	WKH	Antrodia albobrunnea (VU), Diplomitoporus lenis (VU), Skeletocutis odora(VU), Phlebia mellea(VU), Laurilia sulcata(VU), Onnia leporina (NT), Alectoria sarmentosa (NT), phellinus chrysoloma (NT), pseudographis pinicola (NT), Cystostereum murrayi (NT), Leptoporus mollis (NT), Phellinidium ferrugineofuscum (NT), Lobaria pulmonaria (NT), fomitopsis rosea (NT), Phellopilus nigrolimitatus (NT), Picoides tridactylus (NT), Hypogymnia bitteri (NT), Icmadophila ericetorum (LC), Meruliopsis taxicola(LC), Perisoreus infaustus (LC), Nephroma bellum(LC).
D7	6/6	13/22	2015	promise	WKH	Phellinus ferrugineofuscus (NT), Alectoria sarmentosa (NT), Onnia leporina (NT), fomitopsis rosea (NT), Nephroma bellum(LC).
D8	6/6	16/22	2015	promise	WKH	Laurilia sulcata (VU), Phellinidium ferrugineofuscum (NT), fomitopsis rosea (NT), Cystostereum murrayi (NT), phellinus chrysoloma (NT), Phellopilus nigrolimitatus (NT), Alectoria sarmentosa (NT), Picoides tridactylus (NT), Icmadophila ericetorum(LC), Perisoreus infaustus (LC).
D9	6/6	15/22	2015	promise	WKH	Alectoria sarmentosa (NT), phellinus chrysoloma (NT), pseudographis pinicola (NT), Nephroma bellum(LC)

Formatted: Font: 14 pt, Bold

Formatted: Font: Bold

Formatted Table

Formatted: Font: 8 pt

Formatted: Font: 8 pt

Formatted: Font: 8 pt

Formatted: Font: 8 pt

Formatted: Font: 8 pt

Formatted: Font: 8 pt

Formatted: Font: 8 pt

Formatted: Font: 8 pt

Formatted: Font: 8 pt

Formatted: Font: 8 pt

Formatted: Font: 8 pt

Formatted: Font: 8 pt

Formatted: Font: 8 pt

Formatted: Font: 8 pt

Formatted: Font: 8 pt

Formatted: Font: 8 pt

Formatted: Font: 8 pt

Formatted: Font: 8 pt

Table ~~S~~1: Survey result of a~~A~~ll 18 ~~20~~ forests randomly selected from the study area. ~~Size is a category of importance when performing the developed method. A spruce forest >20 hectares are considered large and a spruce forest < 20 hectares are considered small. The exception is if the forest is closer than 100m to another key habitat the size of that key habitat is added and if the sum together becomes larger than 20 hectares the forest is considered large*. Table also shows results from the WKH survey with the developed method.~~ Criteria track 1 - forest likely a WKH if more than 5 criteria are fulfilled. Criteria track 2 - forest likely a WKH if more than eight or nine criteria are fulfilled (depending on the size and surrounding). Results of all~~All~~ findings of red-listed and signal species from field survey.

As mentioned in the introduction the classification of a key habitat is a comprehensive assessment of both the forest and the surrounding landscape. The map in Figure 1 ~~over the area further~~ illustrates that forest D2 is part of a green infrastructure from a landscape perspective. The new method favors large areas and areas in proximity to other WKHs. According to the developed method, a so-called "50/50 rule" can be applied in a continuous forest landscape characterized by high conservation values, especially when borders are hard to delimit [11]. This rule dictates that if 50% of the area reaches the criteria for WKH standard, the entire area should be designated as a WKH. The area of classified WKH in ~~this continuous~~the sourrounding forest landscape ~~has an area of~~cover over 450 hectares and the two deregistered forests cover together ~~much less than half of this (~~less than 1/4th of the size of the surrounding WKH~~)~~. The 50/50-method thus strengthens the assessment to ~~keep the~~ classif~~y~~ication of ~~t~~his forest as a WKH (see F~~f~~igure 1).

The classification of the deregistered WKHs at the time of the field survey is also presented in Table 1. ~~S3 (Appendix 1).~~ Forest D2 is classified as~~as~~ a ~~"~~production forest~~"~~ and the remaining eight deregistered forests were re-labeled as "non-exchangeable~~replaceable~~ promises". A "non-exchangeable ~~replaceable~~ promise" is a ~~somewhat vaguely defined~~ category of voluntary set-aside area with a higher protection from exploitation through forestry than a regular set-aside area~~, but which still lacks proper long-term protection from exploitation~~ [20]~~(Skogsindustrierna 2019)~~. The protection of these areas relies on an agreement between Sveaskog and the county ~~administrative~~ board~~, but they are not protected by law~~ [21]~~(Naturvårdsverket 2015)~~. During 2023, however, these 8 "non-~~replaceable~~exchangeable promises" areas were again reclassified and are now part of a long term protection. Of the more than 61000 hectares

Formatted: Font: Bold

Formatted: Line spacing: single

Formatted: Not Highlight

Formatted: Not Highlight

Formatted: Highlight

of deregistered WKH included in the map of deregistered WKH included in this study, 2149 hectares were included in this new agreement for long-term protection of valuable forests in the mountain region [22, 23]

Fig 1. This is the Fig 1 Title: Map of study area.

This is the Fig 1 legend.

Figure 1: The star in map A denotes the location of the study area in northwestern Sweden, with red dots illustrating the distribution of all deregistered WKH included in the map used in this study [23] . Map B in a closeup of the study area, where all WKHs are indicated in blue and deregistered WKH are indicated with red, all surveyed areas are marked with a label. Map C presents deregistered WKH D2 (now classified as production forest), in a landscape perspective. Boundaries of forests of high conservation value according to the County Administrative Board are delineated by a white line. [23~~(skogsgruppen 2019~~, ~~Skogsstyrelsen 2020,~~24, ~~Skogsstyrelsen 2019,c~~25, Basemap from OpenStreetMap https://www.openstreetmap.org/, ~~Lantmäteriet 2019~~])

3.2 Conservation ~~status~~ values in registered and deregistered WKH.

A majority of the interior of the surveyed forests displayed no clear indications of human disturbance and had clearly never undergone large-scale exploitation. The biodiversity and conservation value of all forests were notably high, all having more than 100 conservation trees per hectare and many red-listed species. The forests are old, dominated by spruce, with a high amount of dead wood and many old living trees with an age over 250 years and draped with pendulous lichens. ~~Species of conservation interest were not part of an exhaustive search, but sub sampled during the field study and the index of the~~ The number of red-listed and signal species in the ~~studied~~ surveyed forests varied from 3 to 21 with an average of 7 ,.4 species per WKH. Full list of species is presented in Table 1 ~~(Appendix 1)~~. The largest deregistered WKH (D6) had the highest number of observed red-listed species, with 18 species belonging to the Swedish red list, of which 5 classified as vulnerable (VU). In the deregistered WKH, D2, currently classified as production forest, nine red-listed species were identified, including two VU species.

3.3 Comparison between findings in studied WKH and ~~set~~ established criteria in new inventory method

Important quantifiable parameters~~Two important properties~~ within the method are ~~in evaluating the conservation status of a forest are~~ the amount of conservation trees, amount of dead wood, ~~and~~ the presence

Formatted: Font: Arial Narrow, 12 pt
Formatted: Font color: Custom Color(RGB(67,67,67))
Formatted: English (United States)
Formatted: English (United States)
Formatted: Font: 16 pt, English (United States)
Formatted: English (United States)
Formatted: Font: 16 pt, English (United States)
Formatted: Font: 16 pt, English (United Kingdom)
Formatted: Font: 16 pt, English (United States)
Formatted: Font: 16 pt, English (United Kingdom)
Formatted: Font: 16 pt, English (United States)
Formatted: Font: 16 pt
Formatted: Font: 16 pt

of old trees and important species. All surveyed forests exceeded the set criteria of a minimum of 20 conservation trees per hectare for track 1. Additionally, the number of spruce conservation trees was also above the criteria in track 1 for all forests, with at least 30 conservation trees per hectare. The total quantity amount of dead wood (>10cm) exceeded the set criteria of a minimum of 60 dead trees per hectare in track 1 for all areas, except for WKH D2. As illustrated in Figure 4, current production forest D2 had a relatively low amount of dead wood per hectare. However, this forest exhibited signs of previous wildfire, which could reduce dead wood quantity without diminishing the conservation value of the forest – an observation critical for evaluating the method. To examine potential statistical differences between the two groups of forests (current WKH and deregistered WKH) a significance test was conducted analyzing deviance with a Chi-square test and 17 degrees of freedom. The analysis of average number of conservation trees per hectare showed a nearly marginally significant difference between the two groups, with slightly higher density observed for the registered key habitats (see Figure 2). This suggests a probability of approximately 5% that the observed difference between registered and unregistered habitats is a false positive caused by stochasticity in the sampling. The From a practical perspective, this p-value is low enough to assume that registered key habitats host a larger number of trees compared to unregistered key habitats ($p=0.0531$). The random effect for habitat indicated significant variability in conservation tree counts between habitats (variance = 3024). The index of numbers of signal and red-listed species found in the two sorts of habitats was also tested in the same way and here Nno significant difference was found between the two groups categories of forests when looking at the difference in number of signal and red-listed species ($p < 0.08$), see Figure 3.

Fig 2. This is the Fig 2 Title: Conservation trees per hectare**.**

This is the Fig 2 legend.

Formatted: Not Highlight

Formatted: Not Highlight

Formatted: Not Highlight

Formatted: Font: 12 pt, Not Highlight

Formatted: Not Highlight

Formatted: Font: 12 pt, Not Highlight

Formatted: Not Highlight

Formatted: Not Highlight

Formatted: Not Highlight

Formatted: Not Highlight

Formatted: Not Highlight

Figure 2: Variation of the mean number of conservation trees per hectare within each forest. The small box plots show the variation within each forest and the large box plots show the variation within the categories registered WKH (R) and deregistered WKH (D). The significance of the effect of registration status on the mean number of conservation trees per habitat was p=0.0531. Red line shows criteria for track 1 by the new inventory method, i.e., 20 conservation trees per hectare. It can be noted that all forests are well above the limit of 20 trees per hectare set as a criteria for WKH.

Fig 3. This is the Fig 3 Title: number of red-listed and signal species**.**

This is the Fig 3 legend.

Figure 3: Variation of the number of red-listed and signal species observed during ~~a short visit~~survey within each forest ~~(thus not the total number of red listed species)~~. The box plots show the variation within the categories registered WKH (R1-9) and deregistered WKH (D1-9). The Chi-square test between the two groups registered and unregistered WKH shows no significant difference in the index of number of red-listed and signal species ($p < 0.08$).

~~3.4 Status of WKH and deregistered WKH in Västerbotten county~~

~~Two important properties in evaluating the conservation status of a forest are the amount of dead wood and the presence of old trees.~~ T~~In general, t~~he total amount of dead wood and living trees of high age in the studied forests was high compared to the criteria in the inventory method. The total average amount of dead wood (both standing and lying) in all areas ~~per hectare~~ was 192 per hectare and is well above the citerias in the method, 50/ hectare in track one and 60/ hectare in track two (See Figure 4) . The average amount of dead wood for each individual area was well above the critaria except for area D2 where the average amount within the area was 62 per hectare an just met the criteria~~shown in figure 4~~. A linear mixed model with habitat as a random effect revealed a significant difference in total deadwood between registered and deregistered key biotopes. Registered key biotopes had higher amount of total deadwood than deregistered ones ($p = 0.000831$). Habitat explained a significant portion of the variation in total deadwood, with a variance of 1133 (SD = 33.67), while residual variance was 1934 (SD = 43.98). These findings suggest that registration status significantly influences deadwood levels, with habitat-specific variation also playing a role. ~~The number of living spruce trees of old age (>250 years) per hectare is shown in Figure 5. Evaluation of age was done according to the field manual (When using this variable in the developed method), 11 of the 18 forests passed,~~

Formatted: Font: Times New Roman, Not Bold, Font color: Auto

Formatted: Normal, Justified, Space Before: 0 pt, After: 6 pt

Formatted: Not Highlight

with more than 10 spruce of old age per hectare, however this variable had a high variation between the sample sites.

Fig 4. This is the Fig 4 Title: Average number of dead wood**.**

This is the Fig 4 legend.

Figure 4: The average total number of dead wood objects per hectare, both standing and lying and standard deviation (SD) with registered showed in purple and deregistered showed in red. The dead wood criteria are 50/hectare in track one (dashed line)and 60/hectare in track two (solid line).

The number of living spruce trees of old age (>250 years) per hectare is shown in Figure 5, 11 of the 18 forests passed, with an average of more than 10 spruce of old age per hectare, however this variable had a high variation between the sample sites.The analysis found no significant difference in the number of old spruce trees between registered and deregistered key biotopes (p = 0.154). The random effect for habitat indicated variability in the number of old spruce trees across habitats (variance = 36.36).

Fig 5. This is the Fig 5 Title: Number of living spruce trees of high age **.**

This is the Fig 5 legend.

Figure 5: Number of living spruce trees of high age >250 years per hectare and standard deviation (SD) in the randomly assigned sample plot in studied forests. Criteria for track 2 (black line) in the developed method is 10 trees per hectare.

3.4 Harvest actions within deregistered key habitats since deregistration

The The majority of the deregistered key habitatsWKH included in this study underwent deregistration between 2015 and 20187, before the SFA stopped to intentify and register WKH in 2021. . CBy ombining the dgoing through the data-base of harvesting cutting reports within with the areas maps of these deregistered key habitatsWKH shows, it has been determined that harvesting activities hasve been conducted within 874 hectares of deregistered WKHkey habitat forest and , 3010 hectares of which were reported for cut harvesting after deregistration (Figure 6).

Formatted: Font: 14 pt

Formatted: Not Highlight

Formatted: Not Highlight

Formatted: Not Highlight

Formatted: Not Highlight

Fig 6. This is the Fig 6 Title: harvested forest and reported forest reported for cutting forest per year-.

This is the Fig 6 legend.

Figure 6
To the left harvested forest in hectares per year within the deregistered key habitats in Sweden. To the right Forest reported for cutting in hectares per year within the deregistered key habitats in Sweden.

Fig 7. This is the Fig 7 Title: Deregistered key habitats included in the new agreement.

This is the Fig 7 legend.

Figure 7
Deregistered key habitats included in the new agreement of permanent protection and the total amount of deregistered key habitats in Sweden (Skogsgruppen 2019).

4. Discussion

SAMMANFATTA RESULTAT

Based on the analysis of the field survey, we conclude that all 18 surveyed registered and deregistered WKHs in this study meet the criteria to be classified as WKH under the new method. These areas represent remnants of old forest in a fragmented forest landscape, typically surrounded by mires, plantations and clear-cuts. The surveyed forests showed high numbers for many of the core qualities within the method like the number of dead wood, conservation trees, trees of high age and important species. Signal and red-listed species were present in all forests, and in six of them species classified as vulnerable (VU) on the red list were found. One of the deregistered WKHs has recently been reclassified as a production forest, while the remaining deregistered WKHs have been incorporated into a permanent protection plan for valuable mountain forests [22]. However, this protection plan does not include most of the deregistered WKHs across Sweden. The deregistration of WKH poses a potential threat to the conservation of valuable forest areas as these habitats are indirectly protected within certified forest management. There can be various reasons behind a deregistration, for example to register the area as another form of nature conservation or to register

the area as a production forest. The results of this study shows that logging has been conducted within many deregistered WKH after deregistration.

4.1 Current classification of deregistered WKH in Västerbotten

Eight of the deregistered forests included in the study are now part of a new agreement for long-term protection of mountain forests. This agreement between the Swedish Environmental Protection Agency and Sveaskog, signed in February 2023, protects nearly 100, 000 hectares of mountain forests, including 48,000 hectares of productive forestland and 50,000 hectares of low productive forestland [22]. Of the 61 000 hectares of deregistered key habitats only 2100 hectares were included in this agreement. The deregistered WKH D2 were deregistration as WKH and registered as production forests, despite having high conservation values according to both this study and previous field surveys [26, 27]. The amount of dead wood and old spruce trees were lower in D2, contributing to this forest not reaching the requirements in track 1. However, there were signs of a previous forest fire in this forest, which can diminish the amount of dead wood. On the other hand, such fires are known to correlate with other biodiversity values. Area D2 fulfill the criteria for being a likely WKH in track 2 and is also a part of a green infrastructure directly connected to other WKHs. This should allow the application of the 50/50 rule, an addition to the new method, which states that if there are uncertainties about classification and 50% of the area is of WKH standard, then the entire area should be registered as a WKH [11]. The consequence of deregister D2 as a WKH is that this old-growth forest with unique biodiversity values, contributing to a green infrastructure by connecting other segments of old growth forests, can now be subjected to clear-cutting with only six weeks of prior notice according to the current Swedish forestry laws [28]. According to previous surveys conducted by the Swedish Society for Nature Conservation and by the County Administrative Board in Västerbotten in 2003, D2 (Located in Abmoberget) should qualify as a WKH [26,27]. The survey report from the County Administrative Board in Västerbotten states that the forest at Abmoberget (see Figure 1) is a large continuous area with very high nature

Formatted: Not Highlight

conservation values and any clear signs of forestry are absent throughout the area [27]. The oldest investigated spruce in the entire area reached the age of 497 years [26]. The County Administrative Board argues that the forest landscape on Abmoberget is of high nature conservation value and can be severely damaged by forestry and other exploitation [27]. In forest D2, nine red-listed species were found during this field study and surveys of the greater Abmoberget area found 13 red-listed species and 11 signal species [27]. The varying conclusions regarding Abmoberget highlight the importance of correctly classifying and delimiting a forest and how a deregistration of a WKH might lead to loss of voulnerable forest areas.

4.2 Evaluating the developed inventory method

The new method is well structured and, to some extent, aligns with the objectives of transparency, objectivity and predictability. However, certain aspects of the method remain inevitably subjective and dependent on the surveyor. The identification of redlisted and signal species, places a lot of responsibility on the surveyor and introduces subjectivity to the method. To address this subjectivity, an independent observer could conduct the survey. The new method was in part developed to (better) consider regional differences, however, it does not consider one crucial regional difference: snow. An unfortunate oversight, as the method is impossible to conduct properly without clear visibility of the forest floor, typical effect of witer conditions across northern Sweden. The forests in the inland of Västerbotten, for example, might be covered by snow up to five months of the year, which stands in contrast to the current rule of a six week prior notice before clear cutting. If this period of notice would be extended, regional weather aspects could be better considered. A longer period between reporting and harvest could provide more time for inspections, which could help prevent the fragmentation of these valuable forests. The ongoing fragmentation of forests is a major driver of ecosystem degradation and biodiversity loss [29,30]. The new method includes guidelines that favor large WKHs and those situated near other WKHs and has the potential to enhance the creation of a less

fragmented forest landscape, promoting larger areas of old-growth forests that are less vulnerable to species extinction. One consequence with a method with stricter critera, in an area with higher proportion of old growth forests and many potentially unregistered WKH, may be that valuable forests may be at risk of falling below the criteria of the new method that would have been included by the old method.

4.3 The significance of WKH and the future of WKH inventory

The concept of WKH was not created as a protection form but a classification of the quality of the forest [17]. The concept was turned into an indirect protection form by sustainable forest companies who use the WKH concept in their criteria. In the standard for both the Forest Stewardship Council (FSC) and the Program for the Endorsement of Forest Certification (PEFC), there are regulations for certified companies to avoid cutting and selling wood from WKHs [16, 31]. In this way the WKH concept indirectly implies a protection status for all certified companies, as a market-driven commitment to save them. The challenge with employing WKH as a protection form lies in the fact that the WKH concept was not originally designed to function as a protected area. Consequently, the WKH concept lacks the inclusion of buffer zones. Another issue arises from the concept's primary focus on identifying hotspots in the landscape, while to ensure a sustainable future for the forest landscape and the species that are depending on it, it is necessary to protect a larger continuous green infrastructure including these hotspots [32,33]. To ensure that these valuable areas of old-growth forest won't be harvested and transformed into production forests we need a common classification and avaliable open information. Without access to information we cannot see the full picture and are unable to follow the ongoing actions within these forests, unfortunately we see a shift towards less avaliable access to information about WHK. Since SFA stopped register WKH, a complete map of WKH in Sweden is no longer available since many companies now only show them in their own system, making it harder to get an overview of ongoing changes. The long term system of finding and registering valuable woodland key habitats occurred

Formatted: Not Highlight

between 1990 – 2019. During 2021 it was decided that SFA no longer should identify and register WKHs, resulting in already registered WKH being deregistered since the SFA no longer had the authority to register these as from June 2019.

4.4 The role of Swedish woodland Key habitats in a global perspective

Deregistration of WKH and higher citeria for classification of WKH in the northwestern part of Sweden, were we find the highest portiotion of old-growth forests does not align with national and international goals for biodiversity conservation and nature protection. By the year 2030 the EU biodiversity strategy sets out a commitment to protect at least 30 % of areas of the land, to enhance biodiversity and ecosystem functions.[34]. At leat one third should be under strict protection and include all remaining primary and old-growth forests [34]. The focus on high yeild wood production during a long period has resulted in loss of natural and diverse forests in many European regions while the forests of north western Sweden still have areas of unique old-growth forest left [5]. Despite local and global goals for restoring terrestrial ecosystems, old growth forests are being harvested at an alarming rate in Sweden [3]. Considering the uinque value of these forests there is a need for more inspections before cutting near WKHs. Proactive measures and a strict system of controls before harvest are essential to ensure that forests with significant nature conservation values are not replaced by clear-cuts followed by planted production forests. Protected areas around the world lost 4.1% of their forested area between 2001 and 2018 which also highligt the importance to keep control over deforestation of valuable forest areas[36].

Formatted: Font: 16 pt, Bold

Formatted: Font: 16 pt, Bold

Formatted: Not Highlight

Formatted: Not Highlight

Formatted: Not Highlight

Formatted: Not Highlight

Formatted: Highlight

5. Conclusion

The quality of the studied registered and deregistered woodland key habitats (WKHs) in Västerbotten County is high, with a low level of human disturbance. Despite the unique values of forests in northwestern Sweden, the developed method for WKH inventory have higher cirteria for WKHs this area. In addition to

regional adaptations in the new method we propose implementing a notice period of at least six snow-free weeks before harvesting a forest. A recommended course of action is to grant the Swedish Forest Agency the authority to conduct surveys of all old growth forests before any cutting or thinning activities take place. There is a also need of a standardized classification system among forest companies, along with open access to information regarding classification of old growth forest areas, ensuring a transparent and secure approach to harvesting old growth forest in Sweden. Deregistered key habitats, with high conservation values, can still be turned into production forests after deregistration, which means that it is important to conduct more controls within these forests to verify the legitimacy of a deregistration and prevent the loss of valuable ecosystems. As emphasized by the SFA, there are most likely many still unidentified WKHs. These areas must be identified and protected to ensure that Woodland key habitats can persist as refuges for threatened species in the future. The green belt of old growth forests in northwestern Sweden still has the potential to support high biodiversity and should be excluded from forestry to meet the European biodiversity strategy.

Formatted: Highlight

~~4. DISCUSSION~~

Formatted: Font: 18 pt

~~Based on the analysis of the field survey we conclude that all the 18 surveyed registered and deregistered WKHs in this study possess the qualities to be classified as a WKH according to the criteria of the new method. These areas represent patches of old forest in a fragmented forest landscape, mostly surrounded by mires, plantations and clear-cuts, without designated buffer zones around the old forests. One of the deregistered WKHs has recently been reclassified as a production forest, while the remaining deregistered WKHs were, at the time of the survey, categorized as 'non-replaceable promise', a classification without any strict protection. The field survey revealed few or no signs of prior exploitation within these areas. Since the time of the field survey, 8 out of the 9 deregistered WKHs have been reclassified and are now part of a~~

permanent protection plan of valuable mountain forests (Naturvårdsverket 2023). This is however not the case for most of the deregistered WKHs as shown in figure 8.

4.1 Current conservation value of registered and deregistered WKH in Västerbotten

The examined forests exhibited a high amount of dead wood and in many of the forests the spruces were draped in pendulous lichens. Signal and red-listed species were present in all forests, and six of them housed species classified as vulnerable (VU) on the red list. The levels of dead wood and conservation trees were notably high compared to the criteria in the developed method for most forests. A noteworthy observation is that the studied WKHs, whether registered or deregistered, often lack buffer zones to clear-cut areas or new plantations. Considering the observed high conservation value of these forests and the presence of trees with very high conservation values on adjacent clear-cut areas of old forests, the study suggests a need for more frequent inspections before cutting near WKHs. This proactive measure is essential to ensuring that forests with significant nature conservation values are not replaced by clear-cuts followed by planted production forests.

Formatted: Line spacing: Double

4.2 No significant differences in analyzed conservation values between registered or deregistered WKH

There was a lower, but not statistically significant, quantity of conservation trees in deregistered WKH, and no significant difference was observed in the number of red-listed species. As illustrated in Figure 4, it is evident that the amount of dead wood in all forests is very high compared to the established criteria, even in the new method. With the exception of forest D2, all forests met the criteria in both tracks 1 and 2. The amount of dead wood and old spruce trees were low in D2, contributing to this forest not reaching the requirements in track 1. However, there were signs of a previous forest fire in this forest, which might diminish these characteristics. On the other hand, such fires are known to correlate with high biodiversity values,

Formatted: Highlight

emphasizing the risk of overlooking crucial forest habitats due to the absence of dead wood following wildfires. All forests in this study satisfy the current criteria to be deemed a WKH, despite the critera beeing stricter than those of the previous method. Valuable forests outside of this study, may be at risk of falling below the criteria of the new method that would have been indirect protected by the old method.

Formatted: Highlight

Formatted: Highlight

4.3 Deregistered WKHs in Västerbotten and their current classification

All examined deregistered WKHs were classified as likely WKHs according to this study, and eight of them still have the quality to be WKH according to Sveaskog. These eight areas, labelled as 'non-replaceable promises' to the County Administrative Board after deregistration (Sveaskog 2019d) are regulated through the agreements between Sveaskog and the County Administrative Board in Västerbotten (Naturvårdsverket 2015). Since the time of the field survey these eight forests have been included in a new agreement for long-term protection of mountain forests. This agreement between the Swedish Environmental Protection Agency and Sveaskog, signed in February 2023, protects nearly 100, 000 hecteras of mountain forests, including 48,000 hectares of productive forestland and 50,000 hectares of low productive forestland(Naturvårdsverket 2023). Forests within the study included in the agreement within their respective areas include; D1,D3,D9 and D4 (Vatioberget), area D5 and D8 (Vatjomyran), area D6 (Välling träsket) and area D7 (Storliden) (Naturvårdsverket 2023). Of the 61 000 hectares of deregistered key habitats only 2100 hectares were included in this agreement of protection. To enhance transparency and secure the long-term protection, we propose that all forests demonstrating the quality of a WKH should remain classified as WKH. This classification serves as a label indicating the conservation value of the forest. A registered WKH does not possess direct legal protection from forestry but is indirectly protected since forest companies certified by FSC are prohibited from harvesting within a WKH (FSC 2010).

Formatted: Font: (Default) Arial Narrow

Formatted: Font: (Default) Arial Narrow

Formatted: Highlight

Commented [UE3]: Ska jag även skriva här om att det ger en missvisande bild av avregistreringen också eftersom att många skogar kan vara avregistrerade utan att anses ha ett sämre värde än WKH

Formatted: Highlight

The deregistration of WKHs followed by re-classification as production forests, despite having the qualities of WKHs, is also apparent. The deregistered WKH (D2) now classified as a production forest does not only fulfill the criteria for being a likely WKH in track 2 but it is also a part of a green infrastructure directly connected to other WKHs. This should allow the application of the 50/50 rule, an addition to the new method, which states that if there are uncertainties about classification and 50% of the area is of WKH standard, then the entire area should be registered as a WKH. The consequence of neglecting to apply the 50/50 rule to D2 is that this old-growth forest with unique biodiversity values, contributing to a green infrastructure by connecting other segments of old growth forests, can now be subjected to clear-cutting with only six weeks of prior notice.

According to surveys conducted by the Swedish Society for Nature Conservation and by the County Administrative Board in Västerbotten in 2003, D2 (Located in Abmoberget) should qualify as a WKH. The survey report from the County Administrative Board in Västerbotten states that the forest at Abmoberget (see Figure 1) is a large continuous area with very high nature conservation values and any clear signs of forestry are absent throughout the area. The oldest investigated spruce in the entire area reached the age of 497 years (Nygren and Eilertsen 2015). The County Administrative Board argues that the forest landscape on Abmoberget is of high nature conservation value and can be severely damaged by forestry and other exploitation (Länsstyrelsen Västerbotten 2017). In forest D2, nine red-listed species were found during this short field study and surveys of the greater Abmoberget area found 13 red-listed species and 11 signal species (Länsstyrelsen Västerbotten 2017). The varying conclusions regarding Abmoberget highlight the importance of correctly classifying and delimited a forest. Results from spatial analysis show that harvesting is taking place in these deregistered key habitats.

4.4 Evaluating the developed inventory method

The quality of the WKHs in this so high that the new method, despite higher criteria, did not affect the classification of already existing WKHs. The new method is well structured and, to some extent, aligns with

the objectives of transparency, objectivity and predictability. However, certain aspects of the method remain inevitably subjective and dependent on the surveyor. Identifying species of interest, for instance, requires prior knowledge of recognizing them, which may vary among surveyors. For example, some of the species live underneath logs of dead wood or are small and difficult to locate. This places a lot of responsibility on the surveyor and introduces subjectivity to the method. To address this subjectivity, an independent observer could conduct the survey. The new method was in part developed to (better) consider regional differences, however, it does not consider one crucial regional difference: snow. An unfortunate oversight, as the current survey method is impossible to conduct properly without clear visibility of the forest floor, typical effect of witer conditions across northern Sweden. The forests in the inland of Västerbotten, for example, might be covered by snow five months of the year, which stands in contrast to the current rule of a six weeks prior notice before clear cutting. If this period of notice would be extended, regional weather aspects could be better considered, and the forest agency would have more time to survey and identify these valuable forests. The new method includes guidelines that favor large WKHs and those situated near other WKHs. This approach has the potential to enhance the creation of a less fragmented forest landscape, promoting larger areas of old-growth forests that are less vulnerable to species extinction.

4.5 The significance of WKH and the future of WKH inventory

The concept of WKH was not created as a protection form but a classification of the quality of the forest (Skogsstyrelsen 2016). The concept was turned into an indirect protection form by sustainable forest companies who use the WKH concept in their criteria. In the standard for both the Forest Stewardship Council (FSC) and the Program for the Endorsement of Forest Certification (PEFC), there are regulations for certified companies to avoid cutting and selling wood from WKHs. Forest companies certified by FSC are not allowed to cut down any WKH, while companies certified by PEFC must save at least 5% of their woodland where WKHs have the highest priority (FSC 2010; PEFC 2017). In this way the WKH concept indirectly implies a

protection status for all certified companies, as a market-driven commitment to save them. The challenge with employing WKH as a protection form lies in the fact that the WKH concept was not originally designed to function as a protected area. Consequently, the WKH concept lacks the inclusion of buffer zones. Another issue arises from the concept's primary focus on identifying small hotspots in the landscape. To ensure a sustainable future for the forest landscape and the species that are depending on it, it is necessary to protect a larger continuous green infrastructure, also incorporating these hotspots (Ericsson et al. 2005, Rybicki and Hanski 2013). To ensure that these valuable areas of old-growth forest won't be lost to production forest we need a common terminology and open communication. Without open access to complete information we cannot see the full picture and are unable to follow the ongoing actions within these forests and unfortunately we see a shift towards even less open access to information about WHK. A complete map of WKH in Sweden is no longer available since many companies have had them removed from the SFA maps and show them only in their own system, making it harder to evaluate ongoing changes. The long term system of finding and registering valuable woodland key habitats occurred between 1990 – 2019. During 2021 it was decided that SFA no longer should identify and register WKHs, resulting in already registered WKH being deregistered since the SFA no longer have the authority to register these as from June 2019.

Formatted: Highlight

Commented [UE4]: Be more specific here

Commented [UE5]: This is another kind of deregistration than before this date.

5. CONCLUSION

Formatted: Font: 18 pt

The quality of the studied spruce forest woodland key habitats (WKHs) in Västerbotten County is high, with a low level of human disturbance. Despite this, many WKHs in the area have been deregistered. The aim of introducing a new inventory method was to provide an objective, predictable and transparent method, with the goal of enhancing the accuracy and reliability of the method. However, whether the new developed method for the northwestern part of Sweden, featuring higher criteria for WKH (compared to other parts of Sweden) aligns with national and international goals for biodiversity and nature protection, is questionable. A recommended course of action is to grant the Swedish Forest Agency the authority to conduct surveys of

Formatted: Highlight

all old growth forests before any cutting or thinning activities take place. In addition to regional adaptations in the new method we propose implementing a notice period of at least six snow-free weeks before harvesting a forest. There is also a need of a standardized terminology and a standardized classification system among forest companies, along with open access to information regarding classification of old growth forest areas, ensuring a transparent and secure approach to harvesting old growth forest in Sweden. Deregistered key habitats, with high conservation values, can still be turned into production forests after deregistration, which means that it is important to conduct more assessments of these forests to verify the legitimacy of deregistration and prevent the loss of these valuable ecosystems. As emphasized by the SFA, there are most likely many still unidentified WKHs. These areas must be identified and protected to ensure that Woodland key habitats can persist as refuges for threatened species in the future. The green belt of old growth forests in northwestern Sweden still has the potential to support high biodiversity and are of great importance for adaptation to climate changes, as well as for mitigating the ongoing mass extinction.

Formatted: Highlight

Acknowledgments.

Formatted: Font: 18 pt

We thank Gudrun Norstedt, Frank Götmark and Tero Härkönen for assisting with design of the field study as well as useful comments on the manuscript. Jonas Nordenström is thanked for helping out with species identification and support in the field. Also thanks to Swedish society for nature conservation and the local forest group of Kronoberg for making the work behind the map of deregistered key habitats.

Formatted: English (United Kingdom)

ReferencesEFERENCES

1. Potapov P., Yaroshenko A., Turubanova S., Dubinin M., Laestadius L., Thies C., Aksenov D., Egorov A., Yesipova Y., Glushkov I., Karpachevskiy M., Kostikova A., Manisha A., Tsybikova E., Zhuravleva I. 2008. Mapping the World's Intact Forest Landscapes by Remote Sensing. Ecology and Society, 13 (2) Map of Intact forest landscapes.

Heino, M.; Kummu, M.; Makkonen, M.; Mulligan, M.; Verburg, P.H.; Jalava, M.; Räsänen, T.A. 2015, Forest loss in protected areas and intact forest landscapes: A global analysis. PLoS ONE 2015,10: e0138918.http://www.intactforests.org/data.ifl.html Retrieved 2020-05-01.

2.

Ahlström, A., Canadell, J. G., & Metcalfe, D. B. (2022). Widespread unquantified conversion of old boreal forests to plantations. Earth's Future, 10, e2022EF003221.

3. https://doi.org/10.1029/2022EF003221

4. Jonsson, B., Svensson, J. Mikusiński, Grzegorz, Manton, Michael, Angelstam, P.. (2019). European Union's Last Intact Forest Landscapes are at A Value Chain Crossroad between Multiple Use and Intensified Wood Production. Forests. 10. 564. 10.3390/f10070564.

Formatted: Font: 18 pt

Formatted: Font: 18 pt

Formatted: Font color: Gray-80%, English (United States)

Formatted: Font color: Auto, English (United States)

Formatted: Indent: Left: 0.5", No bullets or

Formatted: English (United States)

Formatted: Font: Arial Narrow

Field Code Changed

Formatted: Font color: Gray-80%

Formatted: Font: Arial Narrow

Formatted: Indent: Left: 0.5", No bullets or

Formatted: Font: Not Bold

Formatted: List Paragraph, Numbered + Level: 1 + Numbering Style: 1, 2, 3, … + Start at: 1 + Alignment: Left + Aligned at: 0.25" + Indent at: 0.5"

Formatted: Font: Arial Narrow, Font color: Gray-80%

Formatted: List Paragraph, None, Numbered + Level: 1 + Numbering Style: 1, 2, 3, … + Start at: 1 + Alignment: Left + Aligned at: 0.25" + Indent at: 0.5"

Formatted: Font: Arial Narrow, Font color: Gray-80%

Field Code Changed

Formatted: Hyperlink, Font: Arial Narrow, Afar (Ethiopia)

Formatted: Font color: Auto

Formatted: Indent: Left: 0.5", No bullets or

Formatted: Font: Arial Narrow, Font color: Gray-80%

Formatted: Indent: Left: 0.5", No bullets or

5. Sabatini, F. et al. (2018). Where are Europe's last primary forests?. Diversity and Distributions. 10.1111/ddi.12778.

6. Svensson, J. et al. (2018). Landscape trajectory of natural boreal forest loss as an impediment to green infrastructure. Conservation Biology. 33. 10.1111/cobi.13148.

7. ArtDatabanken (2015). "Rödlistade arter i Sverige 2015."

8. Skogsstyrelsen (2023) Skogsstyrelsens signalarter – en komplett förteckning, Version 2023-1, https://www.skogsstyrelsen.se/globalassets/miljo-och-klimat/nyckelbitoper/skogsstyrelsens-signalarter---version2023-1.pdf retrieved 2024-11-19

9. Larsson, A. (red) 2011 Tillståndet i skogen – rödlistade arter i ett nordiskt perspektiv. ArtDatabanken Rapporterar 9. ArtDatabanken SLU, Uppsala

10. Nitare, J. (2019). Skyddsvärd skog Naturvårdsarter och andra kriterier för naturvärdesbedömning. Stibo Graphics, Skogsstyrelsen Jönköping.

11. Skogsstyrelsen (2019,a). "Handbok för nyckelbiotopsinventering." https://www.skogsstyrelsen.se/globalassets/miljo-och-klimat/nyckelbitoper/handbok-nyckelbiotopsinventering.pdf

12. Gustafsson, L., Hannerz, M. 2018. 20 års forskning om nyckelbiotoper – Här är resultaten. Institutionen för ekologi, Sveriges lantbruksuniversitet (SLU), Uppsala. 134 pp.

13. Djupström, L. B., Weslien, J., Schroeder, L. M. (2008). Dead wood and saproxylic beetles in set-aside and non set-aside forests in a boreal region. Forest Ecology and Management, 255: 3340-3350.

14. Skogsstyrelsen. (2019,b, 2019-07-02). "vårt arbete med nyckelbiotoper."

15. Skogsstyrelsen (2019,d), utveckling av metod för nyckelbiotopsinventering i nordvästra Sverige. https://www.skogsstyrelsen.se/globalassets/om-oss/rapporter/rapporter-20222021202020192018/rapport-2019-12-utveckling-av-metod-for-nyckelbiotopsinventering-i-nordvastra-sverige.pdf

16. FSC 2010 Swedish FSC Standard for Forest Certification including SLIMF indicators FSC-STD-SWE-02-02-2010 Sweden Natural, Plantations and SLIMF EN
FSC. 2019. https://se.fsc.org/se-se/klagoml/offentliga-certifierarrapporter retrieved 2019-12-11

Formatted: Indent: Left: 0.5", No bullets or
Formatted: Font: Arial Narrow, Font color: Gray-80%
Formatted: Indent: Left: 0.5", No bullets or
Formatted: Indent: Left: 0.5", No bullets or
Formatted: Font: Arial Narrow, Font color: Gray-80%
Formatted: Indent: Left: 0.5", No bullets or
Formatted: Indent: Left: 0.5", No bullets or
Formatted: Font: Arial Narrow, Font color: Gray-80%
Formatted: Indent: Left: 0.5", No bullets or
Formatted: Indent: Left: 0.5", No bullets or
Formatted: Afar (Ethiopia)
Formatted: Indent: Left: 0.5", No bullets or
Formatted: Swedish (Sweden)
Formatted: Indent: Left: 0.5", No bullets or
Formatted: Font: Arial Narrow, Font color: Gray-80%
Formatted: Indent: Left: 0.5", No bullets or
Formatted: Font: Arial Narrow, Font color: Gray-80%
Formatted: Indent: Left: 0.5", No bullets or
Formatted: Indent: Left: 0.5", No bullets or
Formatted: Indent: Left: 0.5", No bullets or

17. Skogsstyrelsen (2016). "Nulägesbeskrivning om nyckelbiotoper." rapport 7 2016. https://cdn.abicart.com/shop/9098/art33/84164433-105ca2-Nyckelbiotoper_webb.pdf

18. Skogsdata (2018), Aktuella uppgifter om de svenska skogarna från Riksskogstaxeringen. SLU, Sveriges officiella Statistik, Umeå.

19. Claesson, S. (2018). Nulägesbeskrivning av nordvästra Sverige. kunskapsunderlag. Skogsstyrelsen.

20. Skogsindustrierna, (2019). https://www.skogsindustrierna.se/skogsindustrin/hallbarhet/hallbartskogsbruk/frivilligt-avsatt-skog/karta/ retrieved 2019-11-15

21. Naturvårdsverket. 2015. .Skyddsvärda statliga skogar - Kategorisering av områden på Sveaskogs markinnehav i Västerbottens län. publikation 5497.

22. Naturvårdsverket 2023. Fjällskog - överenskommelse om långsiktigt skydd https://www.naturvardsverket.se/om-oss/aktuellt/nyheter-och-pressmeddelanden/2023/februari/vardefulla-fjallskogar-skyddas-i-ny-overenskommelse/fjallskogar--overenskommelse-langsiktigt-skydd/ 2023-05-20

23. Skogsgruppen 2019 https://www.skogsgrupp.se/bolagens-borttagna-nb/ retrieved 2019-06-20

24. Skogsstyrelsen (2020) Map of woodland key habitats from Swedish Forest Agency https://www.skogsstyrelsen.se/sjalvservice/karttjanster/ retrieved 2020-01-20.

25. Skogsstyrelsen (2023), https://www.skogsstyrelsen.se/sjalvservice/karttjanster/geodatatjanster/ Retrieved: 2023-03-02

26. Nygren P., Eilertssen L. 2015, Naturskyddsföreningen Västerbottenslän - Abmoberget https://docplayer.se/156084859-Text-patrik-nygren-forutom-avsnitt-6-6-och-6-7-som-skrivits-av-lilleilertsen. html retrieved 2019-12-01

27. Länsstyrelsen Västerbotten 2017, Skogar med höga naturvärden i Västerbottens län - Länsstyrelsens skogsinventeringar nedan gränsen för fjällnära skog 2003–2015 https://www.lansstyrelsen.se/download/18.304d30c1612954524550dee/1526067883061/2017-09-15_Skogar%20med%20h%C3%B6ga%20naturv%C3%A4rden%20i%20V%C3%A4sterbottens%20l%C 3%A4n.pdf

28. Skogsstyrelsen (2022), Skogsvårdslagstiftningen gällande regler september 2022, https://www.skogsstyrelsen.se/globalassets/lag-och-tillsyn/skogsvardslagen/skogsvardslagstiftningen-2022.pdf

Formatted: Indent: Left: 0.5", No bullets or

Formatted: Font: (Default) Arial

Formatted: Font: Arial Narrow, Font color: Gray-80%

Formatted: Normal, No bullets or numbering

Formatted: Font: Arial Narrow, Font color: Gray-80%

Formatted: Normal, No bullets or numbering

Formatted: Swedish (Sweden)

Formatted: Indent: Left: 0.5", No bullets or

Formatted: Indent: Left: 0.5", No bullets or

Formatted: Font: Arial Narrow

Formatted: Font: Arial Narrow, Kern at 18 pt

Formatted: Font: Arial Narrow, 12 pt

Formatted: Font color: Gray-80%, Swedish (Sweden)

Formatted: List Paragraph

Formatted: Indent: Left: 0.5", No bullets or

Formatted: Indent: Left: 0.5", No bullets or

Formatted: Font: Arial Narrow

Formatted: Font: Arial Narrow, Font color: Gray-80%

Formatted: Indent: Left: 0.5", No bullets or

Formatted: English (United States)

Formatted: Font: Arial Narrow, Font color: Gray-80%

Formatted: Normal, No bullets or numbering

Field Code Changed

Formatted: Indent: Left: 0.5", No bullets or

Formatted: Indent: Left: 0.5", No bullets or

Formatted: Indent: Left: 0.5", No bullets or

29. Pfeifer, M., Lefebvre, V., Peres, C. et al. Creation of forest edges has a global impact on forest vertebrates. Nature 551, 187–191 (2017). https://doi.org/10.1038/nature24457

30. Undin, M., Atrena, A., Carlsson, F. et al. To what extent does surrounding landscape explain stand-level occurrence of conservation-relevant species in fragmented boreal and hemi-boreal forest? – a systematic review. Environ Evid 13, 19 (2024). https://doi.org/10.1186/s13750-024-00346-1

31. PEFC, 2024 https://pefc.se/vara-standarder/svenska-pefc-standarden/pefc-om-nyckelbiotoper

32. Ericsson, Tysk & Berglund, Håkan & Östlund, Lars. (2005). History and biodiversity of woodland key habitats in south boreal Sweden. Biological Conservation. 122. 289-303. 10.1016/j.biocon.2004.07.019.

33. Rybicki, Joel & Hanski, Ilkka. (2013). Species-area relationships and extinctions caused by habitat loss and fragmentation. Ecology letters. 16. 10.1111/ele.12065.

34. European Parliament, Council of the European Union (2024), Regulation (EU) 2024/1991 of the European Parliament and of the Council of 24 June 2024 on nature restoration and amending Regulation (EU) 2022/869 (Text with EEA relevance), ELI: http://data.europa.eu/eli/reg/2024/1991/oj

35. OpenStreetMap, (2021), Basemap from OpenStreetMap https://www.openstreetmap.org/

36. Wade, C. M., Austin, K. G., Cajka, J., Lapidus, D., Everett, K. H., Galperin, D., Maynard, R., & Sobel, A. (2020). What Is Threatening Forests in Protected Areas? A Global Assessment of Deforestation in Protected Areas, 2001–2018. Forests, 11(5), 539. https://doi.org/10.3390/f11050539

37. FAO and UNEP. 2020. The State of the World's Forests 2020. Forests, biodiversity and people. Rome. https://doi.org/10.4060/ca8642en

ArtDatabanken (2015). "Rödlistade arter i Sverige 2015."

Claesson, S. (2018). Nulägesbeskrivning av nordvästra Sverige. kunskapsunderlag. Skogsstyrelsen.

Djupström, L. B., Weslien, J., Schroeder, L. M. (2008). Dead wood and saproxylic beetles in set-aside and non set-aside forests in a boreal region. Forest Ecology and Management, 255: 3340-3350.

Ericsson, Tysk & Berglund, Håkan & Östlund, Lars. (2005). History and biodiversity of woodland key habitats in south boreal Sweden. Biological Conservation. 122. 289-303. 10.1016/j.biocon.2004.07.019.

Esseen Per-Anders and Renhorn Karl-Erik (1998). " Edge Effects on an Epiphytic Lichen in Fragmented Forests." Conservation Biology Vol. 12, No. 6 (Dec., 1998), pp. 1307-1317

FSC 2010 Swedish FSC Standard for Forest Certification including SLIMF indicators FSC-STD-SWE-02-02-2010 Sweden Natural, Plantations and SLIMF EN
FSC. 2019. https://se.fsc.org/se-se/klagoml/offentliga-certifierarrapporter retrieved 2019-12-11

Gustafsson, L., Hannerz, M. 2018. 20 års forskning om nyckelbiotoper – Här är resultaten. Institutionen för ekologi, Sveriges lantbruksuniversitet (SLU), Uppsala. 134 pp.

Hansen, M.C., Potapov, P.V., Moore, R., Hancher, M., Turubanova, S.A.,Tyukavina, A., Thau, D., Stehman, S.V., Goetz, S.J., Loveland, T.R., Kommareddy, A., Egorov, A., Chini., L., Justice, C.O., Townshend, J.R.G. (2013). High-resolution global maps of 21st-century forest cover change. Science 342: 850-853.

Heino, M.; Kummu, M.; Makkonen, M.; Mulligan, M.; Verburg, P.H.; Jalava, M.; Räsänen, T.A. 2015, Forest loss in protected areas and intact forest landscapes: A global analysis. PLoS ONE 2015,10: e0138918.

Jonsson, B., Svensson, J. Mikusiński, Grzegorz, Manton, Michael, Angelstam, P.. (2019). European Union's Last Intact Forest Landscapes are at A Value Chain Crossroad between Multiple Use and Intensified Wood Production. Forests. 10. 564. 10.3390/f10070564.

Lantmäteriet (2019) Background map for mapping in GIS Retrieved 2019-10-01

Larsson, A. (red) 2011 Tillståndet i skogen – rödlistade arter i ett nordiskt perspektiv. ArtDatabanken Rapporterar 9. ArtDatabanken SLU, Uppsala

Länsstyrelsen Västerbotten 2017, Skogar med höga naturvärden i Västerbottens län – Länsstyrelsens skogsinventeringar nedan gränsen för fjällnära skog 2003–2015
https://www.lansstyrelsen.se/download/18.304d30c1612954524550dee/1526067883061/2017-09-15_Skogar%20med%20h%C3%B6ga%20naturv%C3%A4rden%20i%20V%C3%A4sterbottens%20l%C3%A4n.pdf

Naturvårdsverket. 2015. .Skyddsvärda statliga skogar - Kategorisering av områden på Sveaskogs markinnehav i Västerbottens län. publikation 5497.

Naturvårdsverket 2023. Fjällskog – överenskommelse om långsiktigt skydd
https://www.naturvardsverket.se/om-oss/aktuellt/nyheter-och-pressmeddelanden/2023/februari/vardefulla-fjallskogar-skyddas-i-ny-overenskommelse/fjallskogar--overenskommelse-langsiktigt-skydd/ 2023-05-20

Formatted: Swedish (Sweden), Highlight
Formatted: Highlight
Formatted: Afar (Ethiopia), Highlight
Formatted: Highlight
Formatted: Font: Arial Narrow, Font color: Gray-80%, Highlight
Formatted: List Paragraph, Numbered + Level: 1 + Numbering Style: 1, 2, 3, … + Start at: 1 + Alignment: Left + Aligned at: 0.25" + Indent at: 0.5"
Formatted: Font: Arial Narrow, Font color: Gray-80%, English (United States), Highlight
Formatted: English (United States), Highlight
Formatted: Highlight
Formatted: English (United States), Highlight
Formatted: Highlight
Formatted: English (United States), Highlight
Formatted: Highlight
Formatted: Highlight
Formatted: Highlight

Nitare, J. (2019). Skyddsvärd skog Naturvårdsarter och andra kriterier för naturvärdesbedömning. Stibo Graphics, Skogsstyrelsen Jönköping.

Nygren P., Eilertssen L. 2015, Naturskyddsföreningen Västerbottenslän - Abmoberget

Potapov, P., Yaroshenko, A., Turubanova, S., et al. (2008). Mapping the world's intact forest landscapes by remote sensing. Ecol. Soc., 13, 51, available from: http://www.ecologyandsociety.org/vol13/iss2/art51/. retrieved April 8, 2014.

Potapov P., Yaroshenko A., Turubanova S., Dubinin M., Laestadius L., Thies C., Aksenov D., Egorov A., Yesipova Y., Glushkov I., Karpachevskiy M., Kostikova A., Manisha A., Tsybikova E., Zhuravleva I. 2008. Mapping the World's Intact Forest Landscapes by Remote Sensing. Ecology and Society, 13 (2) Map of Intact forest landscapes. http://www.intactforests.org/data.ifl.html Retrieved 2020-05-01.

Renhorn K.-E. , Esseen P.-A. , Palmqvist K. , Sundberg B. Growth and vitality of epiphytic lichens I. Responses to microclimate along a forest edge-interior gradient Oecologia, 109 (1997), pp. 1-9

Rybicki, Joel & Hanski, Ilkka. (2013). Species-area relationships and extinctions caused by habitat loss and fragmentation. Ecology letters. 16. 10.1111/ele.12065.

Sabatini, F. et al. (2018). Where are Europe's last primary forests?. Diversity and Distributions. 10.1111/ddi.12778.

Skogsindustrierna, (2019). https://www.skogsindustrierna.se/skogsindustrin/hallbarhet/hallbartskogsbruk/frivilligt-avsatt-skog/karta/ retrieved 2019-11-15

Skogsgruppen 2019 https://www.skogsgrupp.se/bolagens-borttagna-nb/ retrieved 2019-06-20

Skogsstyrelsen (2016). "Nulägesbeskrivning om nyckelbiotoper." rapport 7 2016.

Skogsstyrelsen (2019,a). "Handbok för nyckelbiotopsinventering."

Skogsstyrelsen. (2019,b, 2019-07-02). "vårt arbete med nyckelbiotoper."

Skogsstyrelsen (2019,c), https://kartor.skogsstyrelsen.se/kartor/ retrieved 2019-10-01

Skogsstyrelsen (2019,d), utveckling av metod för nyckelbiotopsinventering i nordvästra Sverige.

Skogsstyrelsen (2020) Map of woodland key habitats from Swedish Forest Agency https://www.skogsstyrelsen.se/sjalvservice/karttjanster/ retrieved 2020-01-20.

SLU & Sveriges officiella statistik (2018). Forest statistics 2018, Official Statistics of Sweden Swedish University of Agricultural Sciences. Umeå.

Formatted: English (United States), Highlight

Formatted: Highlight

Formatted: English (United States), Highlight

Formatted: Highlight

Formatted: Font: Arial Narrow, Font color: Gray-80%, Highlight

Formatted: List Paragraph, Numbered + Level: 1 + Numbering Style: 1, 2, 3, … + Start at: 1 + Alignment: Left + Aligned at: 0.25" + Indent at: 0.5"

Formatted: Font: Arial Narrow, Highlight

Formatted: Highlight

Formatted: Font: Arial Narrow, Font color: Gray-80%, Highlight

Formatted: Highlight

Formatted: English (United States)

Field Code Changed

Formatted: English (United States)

Formatted: English (United States)

Formatted: English (United States)

Formatted: Line spacing: Multiple 1.15 li

Formatted: Font: 11 pt

Svensson, J. et al. (2018). Landscape trajectory of natural boreal forest loss as an impediment to green infrastructure. Conservation Biology. 33. 10.1111/cobi.13148.

Supporting information

1. S2. Table. Coordinates of plots . table S1 Show the coordinates and number of plots per for all surveyed areas.
2. S3 Table. Results from field survey. Table S2 show the full result from the field survey.

Formatted: Font: 18 pt

Formatted: List Paragraph, Numbered + Level: 1 + Numbering Style: 1, 2, 3, … + Start at: 1 + Alignment: Left + Aligned at: 0.25" + Indent at: 0.5"

Formatted: Font: Times New Roman, Not Bold

SUPPORTING INFORMATION

S1 Appendix – Table with inventory information

Appendix

ID	Size	Border (m)	Criteria track 1	Criteria track 2	deregistered (year)	Class- At time of survey	Survey result	Red-listed and signal species (classification from red list)
R1	small	1538	6/6	18/22		WKH	WKH	phlebia mellea(VU), Dryocopus martius(NT), Lobaria pulmonaria(NT), Lobaria scrobiculata(NT), Alectoria sarmentosa(NT), phellinus chrysoloma(NT), Nephroma bellum(LC).
R2	small	10456	6/6	15/22		WKH	WKH	phellinus chrysoloma (NT), Alectoria sarmentosa (NT), fomitopsis rosea(NT)
R3	large*	11222	5/6	16/22		WKH	WKH	Haploporus odorus (VU), Diplomitoporus lenis (VU), Antrodia albobrunnea (VU), Alectoria sarmentosa (NT), Lobaria pulmonaria (NT), Dryocopus martius (NT), phellinus chrysoloma (NT), Lobaria scrobiculatus (NT), Nephroma bellum(LC), Nephroma resupinatum (LC), Icmadophila ericetorum(LC).
R4	large	14802	6/6	15/22		WKH	WKH	Alectoria sarmentosa (NT), phellinus chrysoloma (NT), Picoides tridactylus(NT), pseudographis pinicola (NT)
R5	small	5402	6/6	12/22		WKH	WKH	Alectoria sarmentosa (NT), phellinus chrysoloma (NT), Lobaria scrobiculata(NT), Icmadophila ericetorum(LC).
R6	large	4714	5/6	14/22		WKH	WKH	Alectoria sarmentosa (NT), Dryocopus martius (NT), Cystostereum murrayi(NT), Phellinus chrysoloma (NT), Lobaria scrobiculatus (NT), Nephroma resupinatum (LC).
R7	large	6925	6/6	16/22		WKH	WKH	Phellinus chrysoloma (NT), Alectoria sarmentosa (NT), Perisoreus infaustus(LC), Matteuccia struthiopteris(LC).
R8	large*	6386	6/6	18/22		WKH	WKH	Fomitopsis rosea (NT), Leptoporus mollis(NT), Picoides tridactylus(NT), Alectoria sarmentosa (NT), Pseudographis pinicola (NT), phellinus chrysoloma (NT), Perisoreus infaustus (LC), Nephroma bellum(LC).
R9	large	9431	6/6	16/22		WKH	WKH	Cystostereum murrayi(NT), Alectoria sarmentosa (NT), phellinus chrysoloma (NT), Onnia leporina(NT), Perisoreus infaustus(LC), Icmadophila ericetorum (LC), Aconitum lycoctonum (LC), Icmadophila ericetorum (LC), Nephroma resupinatum (LC).
D1	large*	4259	6/6	16/22	2015	promise	WKH	Alectoria sarmentosa (NT), phellinus chrysoloma (NT), Icmadophila ericetorum (LC).
D2	large	5470	2/6	12/22	2017	production	WKH	Haploporus odorus (VU), skeletocutis odora (VU), Phellinidium ferrugineofuscum (NT), Carbonicola anthracophila (NT) , Dryocopus martius (NT), pseudographis pinicola (NT), Alectoria sarmentosa (NT), Phellopilus nigrolimitatus (NT), Onnia leporina (NT), Nephroma resupinatum (LC).
D3	large*	2871	6/6	18/22	2015	promise	WKH	Poecile cinctus (VU), Alectoria sarmentosa (NT), phellinus chrysoloma (NT), Onnia leporina(NT),Picoides tridactylus (NT) Nephroma bellum (LC), Nephroma resupinatum (LC), Meruliopsis taxicola (LC).
D4	large	5981	6/6	18/22	2015	promise	WKH	Skeletocutis odora(VU), fomitopsis rosea (NT), phellinus chrysoloma (NT), Alectoria sarmentosa (NT), Phellinidium ferrugineofuscus (NT), Leptoporus mollis (NT), Perisoreus infaustus(LC), Icmadophila ericetorum (LC).
D5	large	10436	6/6	15/22	2015	promise	WKH	Alectoria sarmentosa (NT), Phellinidium ferrugineofuscus,(NT) fomitopsis rosea (NT), Onnia leporina(NT), phellinus chrysoloma (NT), Phellopilus nigrolimitatus (NT), Icmadophila ericetorum (LC).
D6	large	18606	5/6	18/22	2015	promise	WKH	Antrodia albobrunnea (VU), Diplomitoporus lenis (VU), Skeletocutis odora(VU), Phlebia mellea(VU), Laurilia sulcata(VU), Onnia leporina (NT), Alectoria sarmentosa (NT), phellinus chrysoloma (NT), pseudographis pinicola (NT), Cystostereum murrayi (NT), Leptoporus mollis (NT), Phellinidium ferrugineofuscum (NT), Lobaria pulmonaria (NT), fomitopsis rosea (NT), Phellopilus nigrolimitatus (NT), Picoides tridactylus (NT), Hypogymnia bitteri (NT), Icmadophila ericetorum (LC), Meruliopsis taxicola(LC), Perisoreus infaustus (LC), Nephroma bellum(LC).
D7	small	1616	6/6	13/22	2015	promise	WKH	Phellinus ferrugineofuscus (NT), Alectoria sarmentosa (NT), Onnia leporina (NT), fomitopsis rosea (NT), Nephroma bellum(LC).
D8	large	17585	6/6	16/22	2015	promise	WKH	Laurilia sulcata (VU), Phellinidium ferrugineofuscum (NT), fomitopsis rosea (NT), Cystostereum murrayi (NT), phellinus chrysoloma (NT), Phellopilus nigrolimitatus (NT), Alectoria sarmentosa (NT), Picoides tridactylus (NT), Icmadophila ericetorum(LC), Perisoreus infaustus (LC),
D9	large*	1445	6/6	15/22	2015	promise	WKH	Alectoria sarmentosa (NT), phellinus chrysoloma (NT), pseudographis pinicola (NT), Nephroma bellum(LC)

Table S1: All 20 forests randomly selected from the study area. Size is a category of importance when performing the developed method. A spruce forest >20 hectares are considered large and a spruce forest < 20 hectares are considered small. The exception is if the forest is closer than 100m to another key habitat the size of that key habitat is added and if the sum together becomes larger than 20 hectares the forest is considered large*. Table also shows results from the WKH survey with the developed method. Criteria track 1 - forest likely a WKH if more than 5 criteria are fulfilled. Criteria track 2 - forest likely a WKH if more than eight or nine criteria are fulfilled (depending on the size and surrounding). All findings of red-listed and signal species from field survey.

Dear Editor and Reviewers,

Here follows a document of answers to all comments concerning journal requirements and Reviewers. All answers and comments from authors are added in bold below.

We are grateful to both reviewers for all usful feedback and look forward to hear back from you about our revised manuscript.

Best regards

Ulrika Ervander on behalf of all authors

When you're ready to submit your revision, log on to https://www.editorialmanager.com/pone/ and select the 'Submissions Needing Revision' folder to locate your manuscript file.

Please include the following items when submitting your revised manuscript:

- A rebuttal letter that responds to each point raised by the academic editor and reviewer(s). You should upload this letter as a separate file labeled 'Response to Reviewers'.
- A marked-up copy of your manuscript that highlights changes made to the original version. You should upload this as a separate file labeled 'Revised Manuscript with Track Changes'.
- An unmarked version of your revised paper without tracked changes. You should upload this as a separate file labeled 'Manuscript'.

Answers to Journal requirements:

When submitting your revision, we need you to address these additional requirements.

1. Please ensure that your manuscript meets PLOS ONE's style requirements, including those for file naming. The PLOS ONE style templates can be found at

https://journals.plos.org/plosone/s/file?id=wjVg/PLOSOne_formatting_sample_main_body.pdf and

https://journals.plos.org/plosone/s/file?id=ba62/PLOSOne_formatting_sample_title_authors_affiliations.pdf

2. Please note that PLOS ONE has specific guidelines on code sharing for submissions in which author-generated code underpins the findings in the manuscript. In these cases, all author-generated code must be made available without restrictions upon publication of the work. Please review our guidelines at https://journals.plos.org/plosone/s/materials-and-software-sharing#loc-sharing-code and ensure that your code is shared in a way that follows best practice and facilitates reproducibility and reuse.

A: The code used is ready to be shared and will be made avaliable upon publication of the work

3. We note that Figure 1 in your submission contain copyrighted images. All PLOS content is published under the Creative Commons Attribution License (CC BY 4.0), which means that the manuscript, images, and Supporting Information files will be freely available online, and any third party is permitted to access, download, copy, distribute, and use these materials in any way, even commercially, with proper attribution. For more information, see our copyright guidelines: http://journals.plos.org/plosone/s/licenses-and-copyright.

A: The correct copyrite information has been added to the map and ledgend for Fig 1.

4. We notice that your supplementary table is included in the manuscript file. Please remove them and upload them with the file type 'Supporting Information'. Please ensure that each Supporting Information file has a legend listed in the manuscript after the references list.

A: The supplementary table is now removed and added to the manuscript as Table 1 since there were a lot of comments on both the method and the results which we hope can be more clear when showing the summary from the field survey in the the text.

Answers to reviewers comments

[Note: HTML markup is below. Please do not edit.]

Reviewers' comments:

Reviewer's Responses to Questions

Comments to the Author

1. Is the manuscript technically sound, and do the data support the conclusions?

The manuscript must describe a technically sound piece of scientific research with data that supports the conclusions. Experiments must have been conducted rigorously, with appropriate controls, replication, and sample sizes. The conclusions must be drawn appropriately based on the data presented.

Reviewer #1: No

Reviewer #2: Yes

---

2. Has the statistical analysis been performed appropriately and rigorously?

Reviewer #1: No

Reviewer #2: Yes

---

3. Have the authors made all data underlying the findings in their manuscript fully available?

The PLOS Data policy requires authors to make all data underlying the findings described in their manuscript fully available without restriction, with rare exception (please refer to the Data Availability Statement in the manuscript PDF file). The data should be provided as part of the manuscript or its supporting information, or deposited to a public repository. For example, in addition to summary statistics, the data points behind means, medians and variance measures should be available. If there are restrictions on publicly sharing data—e.g. participant privacy or use of data from a third party—those must be specified.

Reviewer #1: No

Reviewer #2: Yes

---

4. Is the manuscript presented in an intelligible fashion and written in standard English?

PLOS ONE does not copyedit accepted manuscripts, so the language in submitted articles must be clear, correct, and unambiguous. Any typographical or grammatical errors should be corrected at revision, so please note any specific errors here.

Reviewer #1: No

Reviewer #2: Yes

---

5. Review Comments to the Author

Please use the space provided to explain your answers to the questions above. You may also include additional comments for the author, including concerns about dual publication, research ethics, or publication ethics. (Please upload your review as an attachment if it exceeds 20,000 characters)

Reviewer #1: Review statement
PlosONE-D24-09128
Status and future prospect of deregistered woodland key habitats in northwestern Sweden

The manuscript describes the important problem of Sweden de-registering the woodland key habitats and delves into the differences between WKHs and deregitered WKHs.
The project aimed to "investigate the performance of the developed survey methods" and "compare the conservation value of registered WKHs and recently deregistered WKHs". The introduction of study is promising and I have clearly least comments on the introduction, but unfortunately falls apart in the methods and results.
Methods section needs to be completely rewritten, it does not give the information required for a scientific article. The results section is very discussive and must be rewritten in a scientific manner, providing results of the tests, references to tables and figures that support the results. The figures are not relevant in all cases and tests results are not shown. The discussion does not set the study in a larger context. Below I give some detailed comments for improvement of the manuscript.

A: We are greatful for all these valuable comments. Appart from all changes listed below the Method section has been adjusted to give more clear information, the result section has been changed and discussive parts has been removed. A lot of changes has been done in the discussion to give this part better structure and to set the study in a larger context.

Detailed comments

L.33. Do you mean that primary boreal forests are located in northwestern Sweden and northwestern Finland? Later in the Introduction L. 67 you say northwestern Russia and Fennoscandia. This is unclear, please specify.

A: Thank you for the input, I can see that it looks unclear and have now changed it. The sentence at L33 was refering to the European Union and the sentence at L67 was refering to Europe. To make it clear I have changed the sentence at L.33 to descripe the conditions in Europe as well.

Keywords: Do not use the same words in title and keywords. The purpose of keywords is to improve findability of the article. The keywords should then be different from the title. I don't think "deregistered woodland key habitats" is a good keyword.

A: Thank you for the input, I have now changed the keywords to improve the findability and not repeat the title. L.49

L. 87 WKHs may serve… and may host.

A: Good comment and this has been changed in L.86

L. 91. Define SFA the first time you use the abbreviation. Also, change Skogsstyrelsen to SFA or Swedish Forest Agency thorough the manuscript.

A: Thank you for this comment, I have changed this in L.82

L. 92. Not all WKHs are old-growth forests. This part of the text gives an impression that they are. Be careful with wording to not give a wrong impression that WKH is a synonym of old-growth forest.

A: This is a great imput and I agree that it was missleading. This has been changed in L.91

L.122. "identified" has a typo.

A: Thank you for pointing out this, the typo is now adjusted L.123

L.123. Change the "Sveriges officiella statistik" to correct, also correct the references in the reference list to be actually found.

A: Thanks for the correction and this has been adjusted in L.122 and in the references.

L. 127. Please, check and correct the sentence.

A: I have adjusted this scentence and hope that this has made it more clear. L.125 -128

L. 129. Key habitat is supposedly same as WKH? Don't change the terminology, the reader starts to wonder if this is the same or different.

A: Thanks for this comment, the intention was to write Wood land key habitat here as well so the misstake has been adjusted and the it will hopefullt be less confusing now with the terminology beeing percistent thtoughout the manuscript. Adjustment done at L.130

L. 132. In scientific, formal writing, "it is" is the right form, not "it's". This happens also elsewhere.

A: This has been adjusted at L.33 and a search for the incorrect form in the manuscript has been done without any results.

L. 134. You should develop more clear study questions. What is meant by performance? What is the "Conservation value"? Number of species? "We investigate" is not good enough, you need to specify what you want to get out of the research, and make sure the reader understands why this is important. Finally, the last sentence in the introduction, stating that "The study highlights the potential consequences that a switch in inventory method... may have on biodiversity". I am not sure if I agree, all these areas were classified as WKHs before and after the inventory methods switch, right? The fact that they were deregistered had not to do with the fact that they were less good, or did it? The forest owners were given a possibility to deregister their WKHs.

A: Thank you for good input, the aim of the study has be more clearly defined at L.134-141. These deregistrations where made before there was an halt in registation and before the SFA stopped register WKH. These are all deregistered before 2018 which made me wonder whats the purpose was behind these deregistrations. The deregistrations of WKH after 2019 are more understandable since they where given a possibility to deregister their WHKs.

L. 152-156. Unclear what is the purpose of this information here?

A: the purpose here is to describe in detail which areas where included in the study and why. Also to give information on how many sample sites should be visited per area according to the guidelines in the new method.

L. 159-167. This section does not give enough information on how the survey was conducted. How large areas were surveyed? How were they surveyed? How many plots per area were surveyed. The latitudes, longitudes, vegetationtypes, general infromation on sites should be given as well. What is old tree, what is a tree with

conservation value? It is not clear enough, please give a reference that these trees have a significant importance for biodiversity if you say they have it. While writing the methods, remember that the reader must be able to repeat the study after reading what you did. CHeck your peers before submission, if you are not sure.

A: Thank you for this imput, the exact coordinates are now added as additional information, in this table it is also included the number of plots per area. The terms "old tree" and tree with conservation value is defined according to the survey method and described breifly at L.168. We have added more detailed information about the criterias in the method at L. 165-168 and more detailed information about the method and definitions can be found in the method described by the SFA.

L. 169-177. I would need to see the protocol that is used. Alternatively, list the criteria in a table.

A: Thank you for this comment we have tried to be more clear but did not want to go through the hole protocol but instead refer to the protocol used. The protocol that was used is published in the report from the SFA – Skogsstyrelsen 2019d, as described in the text and listed among the referenses. Hope that this makes the description clear enough.

L. 180-189. Suddenly you say that you are using conservation trees as your response model. Why them? Why do you think they are important? You do not mention anything in your study questions about conservation trees, but the differences between WKHs and deregistered forests. In line 183 you say habitat is used as independent variable, what is habitat? Why Chi-square test?
What is the Geographical analysis? Why was it done? I see no purpose in study questions. Give the versions and references to R, state the packages used and give the references to them and QGIS. Did you check if the data fulfilled the requirements for the glms? How did you do it?

A: Looking at the criterias in the method used, the data colleted that is quantified are number of conservation trees, number of dead trees (standing and lying), number of really old trees and number of signal and red listed species this is why these are the focus of the result section. To not only get an answer to whether a surveyed area reach the criteria to be classified as a WKH yes or no, but to get quantified data to compare between these areas. I see that we have written habitat in this section but it should have been WKH so this is corrected now. The Geographical analysis was done to be able to study the fequency of harvest actions within the deregistered WKH in Sweden since deregistration. This section has been clarified at L. 191-195

Results
The results are not written correctly. I need to see the results, not to read your statement that the results show something that I don't see. I was wondering if this is

a Results + discussion in one section, but that was not the case. The whole results section must be re-written, below I give examples of what should be corrected.

A: Thank you for the comment and The result section has been adjusted a lot. We hope that the revised result section presents the results more direct and clear and removed diskussions from this section.

Where are the references to tables and figs showing the results of GLM and chi-square? What does 50/50 rule has to do with your results? Where is Table S3? Table S2 doesn't exist at all in the text.

A: Thank you for the input on tables, it was indeed confusing. There was only one table in the appendix, table S1 and this has now been adjusted and the table has been moved to supporting information as required from plosone. Adjustments at L. 206 Since 50/50 rule stated that an entire area should be classified as a WKH if 50% of the area reaches the criteria for WKH standard, the entire area should be designated as a WKH. There by it is part of the result of an WKH inventory to consider this rule. This is further described at L. 210-217

"Non-replacable promise" is an odd term, I suggest you check with the forest company how they would translate it. I don't know what this has to do with results? Doesn't it belong to methods? You knew it already before the study, it was included in your design.

A: We have been trying to get a translation from the forest company but with no result, After consideration we have decided to relable it as "non exchangeable promise".

The information about how the deregistered areas where classified now where not avaliable online at the time so the current classification of the areas where not known at the time of the inventory. The information on classification of the areas was gathered through contact with sveaskog and added to the result therefore. The classification of the areas was later altered and therefore is the even more recent classification of the areas added to the table in supporting information S1. In one way this is a result of the development of these areas after they became deregistered as WKH and that's why it was added as a part of the result section.

L.224. Where did the human disturbance come from? Never mentioned before. Where is the test result for the redlisted species? You say that it was a subsampling, but now what is a subsampling? Can one trust the data? (True zeros?)

A: The result for the redlisted species in number are showed in figure 3. For a more detailed list of speceis at each area see additional information S1 where we have attached full species lists for each study site. One part of the checklist for the inventory is just about taking notes of the area. Here you can

add comments of the area which can be of importance to the validation of the area, things like visible human disturbance is something that is common to take notes on. Signs of thinning or harvesting is important information to classify a forest. Thank you also a good comment about the inventory method of species. This has been better explained and added to the method section at L171-174.

L 244. You should not repeat the methods in the results. The long text 244-253 gives one result: The number of conservation value tree in WKHs and deregistered WKHs did not differ from each other (X2=??, p<0.08).

A: Good input and this has been removed at L. 257-259. The section below has also been adjusted and shortened to make it more clear without to much information. The resultsection has been rewritten a lot and more info on statistics has been added in both result section and method section.

L 276-277. You should say how high was the number of deadwood and if it differs between WKHs and deregistered WKHs. I don't understand where the whiskers come from in fig 4? What is N in this? Not clear in methods how deadwood is counted.

A: This has been adjusted at L. 288 where the number of dead wood in the areas are compared to the criterias within the method. The whiskes is the variation between plots within the area. On the y axis is the number of deadwood per hectare and on the x axis is the different areas. Dead wood is counted according to the method from SFA refered to in the method.

L. 298: This is not mentioned in the study aims, questions or methods. If this was an aim, it must be stated before the result.

A: Thank you for this input, this has been adjusted at L.140 as well as in methods at L.198

Fig 6. I don't understand the difference in left and right. Is this worth a figure?

A: The differences between the figures are as mentioned in the ledgend that one is for the harvested forest within deregistered WKH and one is for cutting reports within same area. This shows two different aspect of the actions going on within these areas and we find it interesting to show both of them.

Fig 7. Isn't this just 2 numbers? Not worth a figure.

A: Thank you for the input and we agree and have removed the figure 7 and instead described this in the text at L. 226-227.

Discussion

L. 318-327. You give an illusion that WKHs are strictly protected, when you specifically say that non-replaceble promises" are not strictly protected. They are as good as WKHs, in general (classified most probably as NO, so no forestry operations allowed). Does the protection has to do with you study? I don't think so. You should discuss your test results, now what happened to your sites after the study.

I didn't read the rest of the discussion, because it did not have any references to other studies, meaning that it is not a real discussion in terms of setting your results into a larger context. You should rewrite the discussion from the beginning.

A: Thank you for this input, the WKH is offcourse not strictly protected but as described indirectrly protected through certification. The non replaceable promises has been tricky to get clear information about but the most recent contact we had with Sveaskog also states that this classification has a high priotity. The decription of this classification has been changed at L.219.

Also greatful for the input on putting the study in a larger context and have added more of this to the discussion alwell as more references to other studies.

Reviewer #2: This study presents interesting results concerning the evaluation of the process of assessment of Woodland Key Habitats (WKH) in the boreal Sweden. This field work helps to evaluate the effectiveness of the criteria currently applied to evaluate primary / old-growth forests which are likely to be object of certification in Sweden and should ideally guide conservation efforts and more generally forest management planning. Specifically, the authors contrast a new set of stringent criteria/thresholds for WKH classification currently applied in the Swedish Northwest with the original set of criteria/thresholds originally widely applied to classify WKHs in Sweden. As the new criteria have caused the deregistration of some WKHs, which enables ordinary timber harvesting, the authors evaluate the possible consequences for biodiversity loss.

While the topic and the field work are certainly of interest for PLOS readers, the manuscript needs major revision to be acceptable for publication. Even if the authors focus mostly on the field sampling they did in the area, they miss to report on a more extensive vision over the process of deregistration in northwestern Sweden. Furthermore, a better description and comparison of the biological value (in terms of species or taxa) of the registered and deregistered WKHs is lacking and must be better discussed, for example commenting on the level of similarity between deregistered and registered WKHs concerning signal and red-listed species or on the IUCN red-list status.

The paper is difficult to read and often it reports concepts more than once without

going deep in their explanation. Specifications on the details of the results lack generality and do not easily deliver messages that can be exported.

The statistical methods must be better explained and their application in the results must be clearly specified.

Finally, even though WKHs are part of a classifying scheme of the forest and do not have legal value for forest protection, in my opinion, the authors must do an effort to envisage their potential role to meet the 2030 targets of the EU Nature Restoration law by adding raw calculations in this sense. This would much increase the value of the paper.

Thanks for valuable comments, the potential role to meet the 2030 targets has been added to the manuscript and its indeed an important potential for these valuable areas. More celar info on similarities and dissimilarities of quantifiable characteristics like old trees, conservation trees and species has been described more clear and species lists has been added to the result section. Statistical methods has been netter explained in the method section and the results has been more clear described in the result section.

Detailed comments are exposed in the attached pdf. – **Now added below**

Comments from Reviewer 2 added and commented below:

L.38 What kind of field inventory was conducted? For example, which taxa or forest structures were evaluated?

A: Thank you for this comment, we have changed the sentence to clarify that it is the novel method for WKH inventory from Swedish forest agency that has been used at L.38

L.39 It must be better specified what "more stringent" means.

A: we feel that more detailed information on what the stringent criteria means can be further developed in the introduction and did not add anything more in the abstract. The meaning of these criteria is better described ad L.130 -135

L.41 It is not clear what "higher criteria" means.

A: To give a better understanding of what the higher criteria means we have changed the sentence at L.42 and more information about this is also added at L130-135 in the introduction.

L.63 What does "expansive" mean in this context?

A: We decided to remove this word L.63

L.63 Do you mean "old-growth" forrests? Be consistent with the terms used in the manuscript.

A: In this sentence we mean intact forest landscape as described in the refered reference Potavo et al 2008, and further in the paper we use the term old-growth forest to describe old natural forest areas.

L.74 As this term is employed locally only in Scandinavia a reference must be added.

A: Thank you for this input, a referens from SFA has been added at line 76.

L.79 Some non-Swedish references would be appreciated here as this is an international journal.

A: A non- Swedish reference has been added here.

L.86 4.6

A: Thanks for noticing and this has been adjusted.

L.91 Swedish Forest Agency?

A: Yes this has been described and added above at L. 83

L.101 visual

A: Good suggestion, this has been changed a t L.101

L Which criterion is used to identify the likely WKH on the map? For example, the identification of suitable forest structures possibly supporting red-listed species via remote sensing or ALS?

A: The information available is different for different areas, there are usually old inventories with information on age or structure or a visual inspection of maps over the area looking for forests that do not look like plantations. This is not really part of the survey done in this method and thereby we do not go into too much details in the paper.

L.112 better specify that the difference is due to the fact that these forests are in mountain area

A: This has now been specified at L.111

L.120 the start of the WKH identification survey in 1990

A: This was a good suggestion and has been added at L.120

L.136 If logging occurs even when forests hosting WKHs are certified, irrespectively of which scheme is applied for WKHs inventory, are there real differences for biodiversity? Does the presence of more or less WKHs qualify the forest area differently in terms of rights of logging?

A: The fact that the many of the forests are well above the higher criteria in the new method is an indication of that higher criteria has already been the standard when identifying WKH in this area. But to show this we need to make a more extensive study. That logging already occurs in WKHs is a different question where is seems that we need to have a better controlling system for logging in vulnerable areas. Some changes to this section have been done at line 136-143.

L.147 More insights about the criteria used for deregistration are needed.

A: The reason why these areas are deregistered is not based on specific criteria's. The study was based on the map of deregistered WKH described in the paper and the criteria to end up on the map is that it used to be a WKH but had been deregistered as WKH. The reasons behind the deregistration of the WKHs included in the field survey is discussed in the discussion.

L.156 This table must be represented as a matrix to allow identification of the fulfilled criteria.

A: The full result matrix will be added as additional information for deeper insight to the fulfilled criteria's for specific areas.

L.162 it is

A: Adjusted at L. 166

L. 179 Are all the reported statistical tests in the results related only with GLMs? If not, please report in this section all tests you applied specifying why you applied them.

A: Thank you for this comment, more details on statistical tests and results from these has been added in the method section and in the result section.

L.182 As the interest is on the effect of the registration status on the response variable, I would have considered habitat as random factor.

A: good input and the statistical method has been changed as described in the method section and habitat has been added as a random factor.

L.187 What would be the scope of this intersection? This must be better specified in the methods.

A: This analysis was done To get an overview of how much harvest actions there has been since the time of deregistration within the deregistered WKH. It

has been further explained in the method section of statistical and geographical analysis at L. 197-201.

L.193. This is due to what? Can you show e.g. a plot with comparison of number of conservation trees and signal species with the old and the new method? It is not clear at all. And if all meet the criteria, why they were deregistered?

A: To better show the differences a table of summery from the field survey has been added in the result section – Table 1. The quantitative comparison of number of conservation trees and signal species is done between the two different classes; deregistered and registered while surveyed with the same method, the new method. No survey has been done with the old method in this study.

L.204 What does this mean and how you come to this conclusion?

A: All areas concluded in this study has been surveyed by following the new method for WKH classification for north western Sweden. The results from that survey is showed in Table 1. The survey is based on a set of criteria as explained in the method section and if an area reaches these criteria the area is likely a WKH and should be classified as this. This is why we state at L.204 that all areas should be classified as WKH according to the new method.

L.218 Are all red dots in Fig. 1A deregistered WKHs? It would be important to provide some summary statistics on the entity of the WKH deregistration over the total n. WKHs?

A: Yes, all red dots are deregistered WKH, this has also been described in the figure legend of figure 1. L 232. This is a good point and some more information on the area of these deregistered WKH has been added at L.228.

L. 239 This is misleading as total quantity is expected as volume per hectare. Instead here you talk about number of deadwood items, not about volume. Please rephrase this sentence.

A: Thank you for this comment, we agree that it was misleading and have rephrased this sentence at L. 251

L.274 This is the first time in the manuscript that you mention this county. Perhaps this should be introduced already in the methods.

A: This is a good input and information about Västerbotten county has now been added to the method section at L.150

L.267 This statement is very general. In the results we report summary numbers, not just descriptive statements. I suppose you are making a comparison here. Then, make this comparison.

A: This was a really good input, this sentence have been adjusted at L. 289-293. More details about the numbers have been presented instead of just a general statement.

L. 294 What is this field manual? Not mentioned in the methods.

A: Thank you for pointing out this, The method includes a field manual and information about this and references has now been added to the method section at L.165-166

L. 322 The legends related with the colours must be clearly reported also in the figures. Hectares is a unit that expresses area of something, which must be reported in the axis.

A: The figure has been adjusted and more information has been added. Since Hectare is the most frequently used unit in this paper we find it more concise to use hectares instead of 10 000 m2.

L.342 there is no figure 8

A: We are very thankful for this comment and have changed to the correct figure number at L.342

L.367 indirectly

A: this has been adjusted at L.367

L.376 hectares

A: this has been adjusted at L.376

L.381 So do you indirectly suggest that there is no need for the new assessment scheme as the old one suffice to identify correctly WKHs?

A: No, we propose that all WKH should be registered as WKH since this is a classification that states that the conservation values of the forests reaches up to a certain level. In this study for example the forests with the classification "promise to the county board" still have the values of a WKH and to make it easy and transparent to follow what happens to these forests it is important to keep the registration. It can be misleading to deregister an area as WKH if the area is still considered to have the values of a WKH.

L.404 delimiting

A: adjusted at L.404

L.407 this county

A: this sentence has been adjusted at 407

L.439 be more specific here.

A: This sentence has been changed and better specified. L.430

L. 437 change method to tool

A: adjustments of this sentence has been done at L.437

---

6. PLOS authors have the option to publish the peer review history of their article (what does this mean?). If published, this will include your full peer review and any attached files.

If you choose “no”, your identity will remain anonymous but your review may still be made public.

Do you want your identity to be public for this peer review? For information about this choice, including consent withdrawal, please see our Privacy Policy.

Reviewer #1: No

Reviewer #2: No
