# Supplementary material for: Status and future prospect of deregistered woodland key habitats in Northwestern Sweden
Source: PLoS One. 2025 Oct 8;20(10):e0331191. doi: 10.1371/journal.pone.0331191 (PMC12507246; doi:10.1371/journal.pone.0331191)
Supplement: Attachment — Submitted filename: Response to Reviewers.docx [file pone.0331191.s004.docx]

**Dear Editor and Reviewers,**

**Here follows a document of answers to all comments concerning journal requirements and Reviewers. All answers and comments from authors are added in bold below.**

**We are grateful to both reviewers for all usful feedback and look forward to hear back from you about our revised manuscript.**

**Best regards**

**Ulrika Ervander on behalf of all authors**

When you're ready to submit your revision, log on to <https://www.editorialmanager.com/pone/> and select the 'Submissions Needing Revision' folder to locate your manuscript file.

Please include the following items when submitting your revised manuscript:

- A rebuttal letter that responds to each point raised by the academic editor and reviewer(s). You should upload this letter as a separate file labeled 'Response to Reviewers'.
- A marked-up copy of your manuscript that highlights changes made to the original version. You should upload this as a separate file labeled 'Revised Manuscript with Track Changes'.
- An unmarked version of your revised paper without tracked changes. You should upload this as a separate file labeled 'Manuscript'.

**Answers to Journal requirements:**

When submitting your revision, we need you to address these additional requirements.

1. Please ensure that your manuscript meets PLOS ONE's style requirements, including those for file naming. The PLOS ONE style templates can be found at

<https://journals.plos.org/plosone/s/file?id=wjVg/PLOSOne_formatting_sample_main_body.pdf> and

<https://journals.plos.org/plosone/s/file?id=ba62/PLOSOne_formatting_sample_title_authors_affiliations.pdf>

2. Please note that PLOS ONE has specific guidelines on code sharing for submissions in which author-generated code underpins the findings in the manuscript. In these cases, all author-generated code must be made available without restrictions upon publication of the work. Please review our guidelines at [https://journals.plos.org/plosone/s/materials-and-software-sharing#loc-sharing-code](https://journals.plos.org/plosone/s/materials-and-software-sharing#loc-sharing-code) and ensure that your code is shared in a way that follows best practice and facilitates reproducibility and reuse.

**A: The code used is ready to be shared and will be made avaliable upon publication of the work**

3. We note that Figure 1 in your submission contain copyrighted images. All PLOS content is published under the Creative Commons Attribution License (CC BY 4.0), which means that the manuscript, images, and Supporting Information files will be freely available online, and any third party is permitted to access, download, copy, distribute, and use these materials in any way, even commercially, with proper attribution. For more information, see our copyright guidelines: <http://journals.plos.org/plosone/s/licenses-and-copyright>.

**A: The correct copyrite information has been added to the map and ledgend for Fig 1.**

4. We notice that your supplementary table is included in the manuscript file. Please remove them and upload them with the file type 'Supporting Information'. Please ensure that each Supporting Information file has a legend listed in the manuscript after the references list.

**A: The supplementary table is now removed and added to the manuscript as Table 1 since there were a lot of comments on both the method and the results which we hope can be more clear when showing the summary from the field survey in the the text.**

**Answers to reviewers comments**

[Note: HTML markup is below. Please do not edit.]

Reviewers' comments:

Reviewer's Responses to Questions

**Comments to the Author**

1. Is the manuscript technically sound, and do the data support the conclusions?

The manuscript must describe a technically sound piece of scientific research with data that supports the conclusions. Experiments must have been conducted rigorously, with appropriate controls, replication, and sample sizes. The conclusions must be drawn appropriately based on the data presented.

Reviewer #1: No

Reviewer #2: Yes

2. Has the statistical analysis been performed appropriately and rigorously?

Reviewer #1: No

Reviewer #2: Yes

3. Have the authors made all data underlying the findings in their manuscript fully available?

The [PLOS Data policy](http://c05y1x9s.r.us-east-2.awstrack.me/L0/http:%2F%2Fwww.plosone.org%2Fstatic%2Fpolicies.action%23sharing/1/010f019132c9c921-ff23a3ca-e391-44a8-9986-cbc479b42048-000000/FBeY6_p8ru_Vq-0SxSrT_igeZsE=171) requires authors to make all data underlying the findings described in their manuscript fully available without restriction, with rare exception (please refer to the Data Availability Statement in the manuscript PDF file). The data should be provided as part of the manuscript or its supporting information, or deposited to a public repository. For example, in addition to summary statistics, the data points behind means, medians and variance measures should be available. If there are restrictions on publicly sharing data—e.g. participant privacy or use of data from a third party—those must be specified.

Reviewer #1: No

Reviewer #2: Yes

4. Is the manuscript presented in an intelligible fashion and written in standard English?

PLOS ONE does not copyedit accepted manuscripts, so the language in submitted articles must be clear, correct, and unambiguous. Any typographical or grammatical errors should be corrected at revision, so please note any specific errors here.

Reviewer #1: No

Reviewer #2: Yes

5. Review Comments to the Author

Please use the space provided to explain your answers to the questions above. You may also include additional comments for the author, including concerns about dual publication, research ethics, or publication ethics. (Please upload your review as an attachment if it exceeds 20,000 characters)

Reviewer #1: Review statement
PlosONE-D24-09128
Status and future prospect of deregistered woodland key habitats in northwestern Sweden

The manuscript describes the important problem of Sweden de-registering the woodland key habitats and delves into the differences between WKHs and deregitered WKHs.
The project aimed to “investigate the performance of the developed survey methods” and “compare the conservation value of registered WKHs and recently deregistered WKHs”. The introduction of study is promising and I have clearly least comments on the introduction, but unfortunately falls apart in the methods and results.
Methods section needs to be completely rewritten, it does not give the information required for a scientific article. The results section is very discussive and must be rewritten in a scientific manner, providing results of the tests, references to tables and figures that support the results. The figures are not relevant in all cases and tests results are not shown. The discussion does not set the study in a larger context. Below I give some detailed comments for improvement of the manuscript.

**A: We are greatful for all these valuable comments. Appart from all changes listed below the Method section has been adjusted to give more clear information, the result section has been changed and discussive parts has been removed. A lot of changes has been done in the discussion to give this part better structure and to set the study in a larger context.**

Detailed comments

L.33. Do you mean that primary boreal forests are located in northwestern Sweden and northwestern Finland? Later in the Introduction L. 67 you say northwestern Russia and Fennoscandia. This is unclear, please specify.

**A: Thank you for the input, I can see that it looks unclear and have now changed it. The sentence at L33 was refering to the European Union and the sentence at L67 was refering to Europe. To make it clear I have changed the sentence at L.33 to descripe the conditions in Europe as well.**

Keywords: Do not use the same words in title and keywords. The purpose of keywords is to improve findability of the article. The keywords should then be different from the title. I don’t think “deregistered woodland key habitats” is a good keyword.

**A: Thank you for the input, I have now changed the keywords to improve the findability and not repeat the title. L.49**

L. 87 WKHs may serve… and may host.

**A: Good comment and this has been changed in L.86**

L. 91. Define SFA the first time you use the abbreviation. Also, change Skogsstyrelsen to SFA or Swedish Forest Agency thorough the manuscript.

**A: Thank you for this comment, I have changed this in L.82**

L. 92. Not all WKHs are old-growth forests. This part of the text gives an impression that they are. Be careful with wording to not give a wrong impression that WKH is a synonym of old-growth forest.

**A: This is a great imput and I agree that it was missleading. This has been changed in L.91**

L.122. “identified” has a typo.

**A: Thank you for pointing out this, the typo is now adjusted L.123**

L.123. Change the “Sveriges officiella statistik" to correct, also correct the references in the reference list to be actually found.

**A: Thanks for the correction and this has been adjusted in L.122 and in the references.**

L. 127. Please, check and correct the sentence.

**A: I have adjusted this scentence and hope that this has made it more clear. L.125 -128**

L. 129. Key habitat is supposedly same as WKH? Don’t change the terminology, the reader starts to wonder if this is the same or different.

**A: Thanks for this comment, the intention was to write Wood land key habitat here as well so the misstake has been adjusted and the it will hopefullt be less confusing now with the terminology beeing percistent thtoughout the manuscript. Adjustment done at L.130**

L. 132. In scientific, formal writing, “it is” is the right form, not “it’s”. This happens also elsewhere.

**A: This has been adjusted at L.33 and a search for the incorrect form in the manuscript has been done without any results.**

L. 134. You should develop more clear study questions. What is meant by performance? What is the “Conservation value”? Number of species? “We investigate” is not good enough, you need to specify what you want to get out of the research, and make sure the reader understands why this is important. Finally, the last sentence in the introduction, stating that “The study highlights the potential consequences that a switch in inventory method... may have on biodiversity”. I am not sure if I agree, all these areas were classified as WKHs before and after the inventory methods switch, right? The fact that they were deregistered had not to do with the fact that they were less good, or did it? The forest owners were given a possibility to deregister their WKHs.

**A: Thank you for good input, the aim of the study has be more clearly defined at L.134-141. These deregistrations where made before there was an halt in registation and before the SFA stopped register WKH. These are all deregistered before 2018 which made me wonder whats the purpose was behind these deregistrations. The deregistrations of WKH after 2019 are more understandable since they where given a possibility to deregister their WHKs.**

L. 152-156. Unclear what is the purpose of this information here?

**A: the purpose here is to describe in detail which areas where included in the study and why. Also to give information on how many sample sites should be visited per area according to the guidelines in the new method.**

L. 159-167. This section does not give enough information on how the survey was conducted. How large areas were surveyed? How were they surveyed? How many plots per area were surveyed. The latitudes, longitudes, vegetationtypes, general infromation on sites should be given as well. What is old tree, what is a tree with conservation value? It is not clear enough, please give a reference that these trees have a significant importance for biodiversity if you say they have it. While writing the methods, remember that the reader must be able to repeat the study after reading what you did. CHeck your peers before submission, if you are not sure.

**A: Thank you for this imput, the exact coordinates are now added as additional information, in this table it is also included the number of plots per area. The terms “old tree” and tree with conservation value is defined according to the survey method and described breifly at L.168. We have added more detailed information about the criterias in the method at L. 165-168 and more detailed information about the method and definitions can be found in the method described by the SFA.**

L. 169-177. I would need to see the protocol that is used. Alternatively, list the criteria in a table.

**A: Thank you for this comment we have tried to be more clear but did not want to go through the hole protocol but instead refer to the protocol used. The protocol that was used is published in the report from the SFA – Skogsstyrelsen 2019d, as described in the text and listed among the referenses. Hope that this makes the description clear enough.**

L. 180-189. Suddenly you say that you are using conservation trees as your response model. Why them? Why do you think they are important? You do not mention anything in your study questions about conservation trees, but the differences between WKHs and deregistered forests. In line 183 you say habitat is used as independent variable, what is habitat? Why Chi-square test?
What is the Geographical analysis? Why was it done? I see no purpose in study questions. Give the versions and references to R, state the packages used and give the references to them and QGIS. Did you check if the data fulfilled the requirements for the glms? How did you do it?

**A: Looking at the criterias in the method used, the data colleted that is quantified are number of conservation trees, number of dead trees (standing and lying), number of really old trees and number of signal and red listed species this is why these are the focus of the result section. To not only get an answer to whether a surveyed area reach the criteria to be classified as a WKH yes or no, but to get quantified data to compare between these areas. I see that we have written habitat in this section but it should have been WKH so this is corrected now. The Geographical analysis was done to be able to study the fequency of harvest actions within the deregistered WKH in Sweden since deregistration. This section has been clarified at L. 191-195**

Results
The results are not written correctly. I need to see the results, not to read your statement that the results show something that I don’t see. I was wondering if this is a Results + discussion in one section, but that was not the case. The whole results section must be re-written, below I give examples of what should be corrected.

**A: Thank you for the comment and The result section has been adjusted a lot. We hope that the revised result section presents the results more direct and clear and removed diskussions from this section.**

Where are the references to tables and figs showing the results of GLM and chi-square? What does 50/50 rule has to do with your results? Where is Table S3? Table S2 doesn’t exist at all in the text.

**A: Thank you for the input on tables, it was indeed confusing. There was only one table in the appendix, table S1 and this has now been adjusted and the table has been moved to supporting information as required from plosone. Adjustments at L. 206 Since 50/50 rule stated that an entire area should be classified as a WKH if 50% of the area reaches the criteria for WKH standard, the entire area should be designated as a WKH. There by it is part of the result of an WKH inventory to consider this rule. This is further described at L. 210-217**

“Non-replacable promise” is an odd term, I suggest you check with the forest company how they would translate it. I don’t know what this has to do with results? Doesn’t it belong to methods? You knew it already before the study, it was included in your design.

**A: We have been trying to get a translation from the forest company but with no result, After consideration we have decided to relable it as “non exchangeable promise”.**

**The information about how the deregistered areas where classified now where not avaliable online at the time so the current classification of the areas where not known at the time of the inventory. The information on classification of the areas was gathered through contact with sveaskog and added to the result therefore. The classification of the areas was later altered and therefore is the even more recent classification of the areas added to the table in supporting information S1. In one way this is a result of the development of these areas after they became deregistered as WKH and that’s why it was added as a part of the result section.**

L.224. Where did the human disturbance come from? Never mentioned before. Where is the test result for the redlisted species? You say that it was a subsampling, but now what is a subsampling? Can one trust the data? (True zeros?)

**A: The result for the redlisted species in number are showed in figure 3. For a more detailed list of speceis at each area see additional information S1 where we have attached full species lists for each study site. One part of the checklist for the inventory is just about taking notes of the area. Here you can add comments of the area which can be of importance to the validation of the area, things like visible human disturbance is something that is common to take notes on. Signs of thinning or harvesting is important information to classify a forest. Thank you also a good comment about the inventory method of species. This has been better explained and added to the method section at L171-174.**

L 244. You should not repeat the methods in the results. The long text 244-253 gives one result: The number of conservation value tree in WKHs and deregistered WKHs did not differ from each other (X2=??, p<0.08).

**A: Good input and this has been removed at L. 257-259. The section below has also been adjusted and shortened to make it more clear without to much information. The resultsection has been rewritten a lot and more info on statistics has been added in both result section and method section.**

L 276-277. You should say how high was the number of deadwood and if it differs between WKHs and deregistered WKHs. I don’t understand where the whiskers come from in fig 4? What is N in this? Not clear in methods how deadwood is counted.

**A: This has been adjusted at L. 288 where the number of dead wood in the areas are compared to the criterias within the method. The whiskes is the variation between plots within the area. On the y axis is the number of deadwood per hectare and on the x axis is the different areas. Dead wood is counted according to the method from SFA refered to in the method.**

L. 298: This is not mentioned in the study aims, questions or methods. If this was an aim, it must be stated before the result.

**A: Thank you for this input, this has been adjusted at L.140 as well as in methods at L.198**

Fig 6. I don’t understand the difference in left and right. Is this worth a figure?

**A: The differences between the figures are as mentioned in the ledgend that one is for the harvested forest within deregistered WKH and one is for cutting reports within same area. This shows two different aspect of the actions going on within these areas and we find it interesting to show both of them.**

Fig 7. Isn’t this just 2 numbers? Not worth a figure.

**A: Thank you for the input and we agree and have removed the figure 7 and instead described this in the text at L. 226-227.**

Discussion

L. 318-327. You give an illusion that WKHs are strictly protected, when you specifically say that non-replaceble promises” are not strictly protected. They are as good as WKHs, in general (classified most probably as NO, so no forestry operations allowed). Does the protection has to do with you study? I don’t think so. You should discuss your test results, now what happened to your sites after the study.

I didn’t read the rest of the discussion, because it did not have any references to other studies, meaning that it is not a real discussion in terms of setting your results into a larger context. You should rewrite the discussion from the beginning.

**A: Thank you for this input, the WKH is offcourse not strictly protected but as described indirectrly protected through certification. The non replaceable promises has been tricky to get clear information about but the most recent contact we had with Sveaskog also states that this classification has a high priotity. The decription of this classification has been changed at L.219.**

**Also greatful for the input on putting the study in a larger context and have added more of this to the discussion alwell as more references to other studies.**

Reviewer #2: This study presents interesting results concerning the evaluation of the process of assessment of Woodland Key Habitats (WKH) in the boreal Sweden. This field work helps to evaluate the effectiveness of the criteria currently applied to evaluate primary / old-growth forests which are likely to be object of certification in Sweden and should ideally guide conservation efforts and more generally forest management planning. Specifically, the authors contrast a new set of stringent criteria/thresholds for WKH classification currently applied in the Swedish Northwest with the original set of criteria/thresholds originally widely applied to classify WKHs in Sweden. As the new criteria have caused the deregistration of some WKHs, which enables ordinary timber harvesting, the authors evaluate the possible consequences for biodiversity loss.

While the topic and the field work are certainly of interest for PLOS readers, the manuscript needs major revision to be acceptable for publication. Even if the authors focus mostly on the field sampling they did in the area, they miss to report on a more extensive vision over the process of deregistration in northwestern Sweden. Furthermore, a better description and comparison of the biological value (in terms of species or taxa) of the registered and deregistered WKHs is lacking and must be better discussed, for example commenting on the level of similarity between deregistered and registered WKHs concerning signal and red-listed species or on the IUCN red-list status.

The paper is difficult to read and often it reports concepts more than once without going deep in their explanation. Specifications on the details of the results lack generality and do not easily deliver messages that can be exported.

The statistical methods must be better explained and their application in the results must be clearly specified.

Finally, even though WKHs are part of a classifying scheme of the forest and do not have legal value for forest protection, in my opinion, the authors must do an effort to envisage their potential role to meet the 2030 targets of the EU Nature Restoration law by adding raw calculations in this sense. This would much increase the value of the paper.

**Thanks for valuable comments, the potential role to meet the 2030 targets has been added to the manuscript and its indeed an important potential for these valuable areas. More celar info on similarities and dissimilarities of quantifiable characteristics like old trees, conservation trees and species has been described more clear and species lists has been added to the result section. Statistical methods has been netter explained in the method section and the results has been more clear described in the result section.**

Detailed comments are exposed in the attached pdf. – **Now added below**

**Comments from Reviewer 2 added and commented below:**

L.38 What kind of field inventory was conducted? For example, which taxa or forest structures were evaluated?

**A: Thank you for this comment, we have changed the sentence to clarify that it is the novel method for WKH inventory from Swedish forest agency that has been used at L.38**

L.39 It must be better specified what "more stringent" means.

**A: we feel that more detailed information on what the stringent criteria means can be further developed in the introduction and did not add anything more in the abstract. The meaning of these criteria is better described ad L.130 -135**

L.41 It is not clear what "higher criteria" means.

**A: To give a better understanding of what the higher criteria means we have changed the sentence at L.42 and more information about this is also added at L130-135 in the introduction.**

L.63 What does "expansive" mean in this context?

**A: We decided to remove this word L.63**

L.63 Do you mean "old-growth" forrests? Be consistent with the terms used in the manuscript.

**A: In this sentence we mean intact forest landscape as described in the refered reference Potavo et al 2008, and further in the paper we use the term old-growth forest to describe old natural forest areas.**

L.74 As this term is employed locally only in Scandinavia a reference must be added.

**A: Thank you for this input, a referens from SFA has been added at line 76.**

L.79 Some non-Swedish references would be appreciated here as this is an international journal.

A: A non- Swedish reference has been added here.

L.86 4.6

**A: Thanks for noticing and this has been adjusted.**

L.91 Swedish Forest Agency?

**A: Yes this has been described and added above at L. 83**

L.101 visual

**A: Good suggestion, this has been changed a t L.101**

L Which criterion is used to identify the likely WKH on the map? For example, the identification of suitable forest structures possibly supporting red-listed species via remote sensing or ALS?

**A: The information available is different for different areas, there are usually old inventories with information on age or structure or a visual inspection of maps over the area looking for forests that do not look like plantations. This is not really part of the survey done in this method and thereby we do not go into too much details in the paper.**

L.112 better specify that the difference is due to the fact that these forests are in mountain area

**A: This has now been specified at L.111**

L.120 the start of the WKH identification survey in 1990

**A: This was a good suggestion and has been added at L.120**

L.136 If logging occurs even when forests hosting WKHs are certified, irrespectively of which scheme is applied for WKHs inventory, are there real differences for biodiversity? Does the presence of more or less WKHs qualify the forest area differently in terms of rights of logging?

**A: The fact that the many of the forests are well above the higher criteria in the new method is an indication of that higher criteria has already been the standard when identifying WKH in this area. But to show this we need to make a more extensive study. That logging already occurs in WKHs is a different question where is seems that we need to have a better controlling system for logging in vulnerable areas. Some changes to this section have been done at line 136-143.**

L.147 More insights about the criteria used for deregistration are needed.

**A: The reason why these areas are deregistered is not based on specific criteria’s. The study was based on the map of deregistered WKH described in the paper and the criteria to end up on the map is that it used to be a WKH but had been deregistered as WKH. The reasons behind the deregistration of the WKHs included in the field survey is discussed in the discussion.**

L.156 This table must be represented as a matrix to allow identification of the fulfilled criteria.

**A: The full result matrix will be added as additional information for deeper insight to the fulfilled criteria’s for specific areas.**

L.162 it is

**A: Adjusted at L. 166**

L. 179 Are all the reported statistical tests in the results related only with GLMs? If not, please report in this section all tests you applied specifying why you applied them.

**A: Thank you for this comment, more details on statistical tests and results from these has been added in the method section and in the result section.**

L.182 As the interest is on the effect of the registration status on the response variable, I would have considered habitat as random factor.

**A: good input and the statistical method has been changed as described in the method section and habitat has been added as a random factor.**

L.187 What would be the scope of this intersection? This must be better specified in the methods.

**A: This analysis was done To get an overview of how much harvest actions there has been since the time of deregistration within the deregistered WKH. It has been further explained in the method section of statistical and geographical analysis at L. 197-201.**

L.193. This is due to what? Can you show e.g. a plot with comparison of number of conservation trees and signal species with the old and the new method? It is not clear at all. And if all meet the criteria, why they were deregistered?

**A: To better show the differences a table of summery from the field survey has been added in the result section – Table 1. The quantitative comparison of number of conservation trees and signal species is done between the two different classes; deregistered and registered while surveyed with the same method, the new method. No survey has been done with the old method in this study.**

L.204 What does this mean and how you come to this conclusion?

**A: All areas concluded in this study has been surveyed by following the new method for WKH classification for north western Sweden. The results from that survey is showed in Table 1. The survey is based on a set of criteria as explained in the method section and if an area reaches these criteria the area is likely a WKH and should be classified as this. This is why we state at L.204 that all areas should be classified as WKH according to the new method.**

L.218 Are all red dots in Fig. 1A deregistered WKHs? It would be important to provide some summary statistics on the entity of the WKH deregistration over the total n. WKHs?

**A: Yes, all red dots are deregistered WKH, this has also been described in the figure legend of figure 1. L 232. This is a good point and some more information on the area of these deregistered WKH has been added at L.228.**

L. 239 This is misleading as total quantity is expected as volume per hectare. Instead here you talk about number of deadwood items, not about volume. Please rephrase this sentence.

**A: Thank you for this comment, we agree that it was misleading and have rephrased this sentence at L. 251**

L.274 This is the first time in the manuscript that you mention this county. Perhaps this should be introduced already in the methods.

**A: This is a good input and information about Västerbotten county has now been added to the method section at L.150**

L.267 This statement is very general. In the results we report summary numbers, not just descriptive statements. I suppose you are making a comparison here. Then, make this comparison.

**A: This was a really good input, this sentence have been adjusted at L. 289-293. More details about the numbers have been presented instead of just a general statement.**

L. 294 What is this field manual? Not mentioned in the methods.

**A: Thank you for pointing out this, The method includes a field manual and information about this and references has now been added to the method section at L.165-166**

L. 322 The legends related with the colours must be clearly reported also in the figures. Hectares is a unit that expresses area of something, which must be reported in the axis.

**A: The figure has been adjusted and more information has been added. Since Hectare is the most frequently used unit in this paper we find it more concise to use hectares instead of 10 000 m2.**

L.342 there is no figure 8

**A: We are very thankful for this comment and have changed to the correct figure number at L.342**

L.367 indirectly

**A: this has been adjusted at L.367**

L.376 hectares

**A: this has been adjusted at L.376**

L.381 So do you indirectly suggest that there is no need for the new assessment scheme as the old one suffice to identify correctly WKHs?

**A: No, we propose that all WKH should be registered as WKH since this is a classification that states that the conservation values of the forests reaches up to a certain level. In this study for example the forests with the classification “promise to the county board” still have the values of a WKH and to make it easy and transparent to follow what happens to these forests it is important to keep the registration. It can be misleading to deregister an area as WKH if the area is still considered to have the values of a WKH.**

L.404 delimiting

**A: adjusted at L.404**

L.407 this county

**A: this sentence has been adjusted at 407**

L.439 be more specific here.

**A: This sentence has been changed and better specified. L.430**

L. 437 change method to tool

**A: adjustments of this sentence has been done at L.437**

6. PLOS authors have the option to publish the peer review history of their article ([what does this mean?](https://c05y1x9s.r.us-east-2.awstrack.me/L0/https:%2F%2Fjournals.plos.org%2Fplosone%2Fs%2Feditorial-and-peer-review-process%23loc-peer-review-history/1/010f019132c9c921-ff23a3ca-e391-44a8-9986-cbc479b42048-000000/em-c_zcEGMgliW4siDqeP2_JREk=171)). If published, this will include your full peer review and any attached files.


If you choose “no”, your identity will remain anonymous but your review may still be made public.


**Do you want your identity to be public for this peer review?** For information about this choice, including consent withdrawal, please see our [Privacy Policy](https://c05y1x9s.r.us-east-2.awstrack.me/L0/https:%2F%2Fwww.plos.org%2Fprivacy-policy/1/010f019132c9c921-ff23a3ca-e391-44a8-9986-cbc479b42048-000000/tNWdR5vJRz0VQPMbsKTejP_bdWg=171).

Reviewer #1: No

Reviewer #2: No
