# Supplementary material for: Status and future prospect of deregistered woodland key habitats in Northwestern Sweden
Source: PLoS One. 2025 Oct 8;20(10):e0331191. doi: 10.1371/journal.pone.0331191 (PMC12507246; doi:10.1371/journal.pone.0331191)
Supplement: Attachment — Submitted filename: Response to reviewers -250629.docx [file pone.0331191.s006.docx]

**Response to Reviewers**

Date: 2025-05-13

Manuscript: *PONE-D-24-09128R1 - Status and future prospect of deregistered woodland key habitats in northwestern Sweden*

**Journal Requirements:**

Please review your reference list to ensure that it is complete and correct. If you have cited papers that have been retracted, please include the rationale for doing so in the manuscript text, or remove these references and replace them with relevant current references. Any changes to the reference list should be mentioned in the rebuttal letter that accompanies your revised manuscript. If you need to cite a retracted article, indicate the article’s retracted status in the References list and also include a citation and full reference for the retraction notice.

**Response:**

The reference list has been thoroughly reviewed, and the following changes (in the former version of the manscript) have been made to ensure accuracy and completeness:

- **Reference no. 7:** Updated with correct and complete citation information.
- **Reference no. 8:** Updated with correct and complete citation information.
- **Reference no. 15:** Updated with correct and complete citation information.
- **Reference no. 18:** Updated with correct and complete citation information.
- **Reference no. 19:** Updated with correct and complete citation information.
- **Previous Reference no. 23:** This reference has been removed, as the original web page is no longer accessible. The data previously cited from this source are now included as supplementary information, with permission from the authors Johan Marand and Erland Lindblad. The supplementary file is named skogsgruppen_data_2019.shp.

In addition to these changes, we have ensured that all references are formatted according to the **Vancouver** style, in alignment with **PLOS ONE** reference guidelines.

[Note: HTML markup is below. Please do not edit.]

Reviewers' comments:

Reviewer's Responses to Questions

**Comments to the Author**

1. If the authors have adequately addressed your comments raised in a previous round of review and you feel that this manuscript is now acceptable for publication, you may indicate that here to bypass the “Comments to the Author” section, enter your conflict of interest statement in the “Confidential to Editor” section, and submit your "Accept" recommendation.

Reviewer #1: (No Response)

Reviewer #2: All comments have been addressed

2. Is the manuscript technically sound, and do the data support the conclusions?

The manuscript must describe a technically sound piece of scientific research with data that supports the conclusions. Experiments must have been conducted rigorously, with appropriate controls, replication, and sample sizes. The conclusions must be drawn appropriately based on the data presented.

Reviewer #1: Partly

Reviewer #2: Partly

3. Has the statistical analysis been performed appropriately and rigorously?

Reviewer #1: I Don't Know

Reviewer #2: Yes

4. Have the authors made all data underlying the findings in their manuscript fully available?

The [PLOS Data policy](http://track.editorialmanager.com/CL0/http:%2F%2Fwww.plosone.org%2Fstatic%2Fpolicies.action%23sharing/1/010f0196635ba12e-7dce4a2f-fab2-4d9e-b7d5-3bb3627c5b9e-000000/UiipNjkjC6tY8fl_1gQfFzZHiXt-yJDgxf_RJV7MsUM=208) requires authors to make all data underlying the findings described in their manuscript fully available without restriction, with rare exception (please refer to the Data Availability Statement in the manuscript PDF file). The data should be provided as part of the manuscript or its supporting information, or deposited to a public repository. For example, in addition to summary statistics, the data points behind means, medians and variance measures should be available. If there are restrictions on publicly sharing data—e.g. participant privacy or use of data from a third party—those must be specified.

Reviewer #1: Yes

Reviewer #2: Yes

5. Is the manuscript presented in an intelligible fashion and written in standard English?

PLOS ONE does not copyedit accepted manuscripts, so the language in submitted articles must be clear, correct, and unambiguous. Any typographical or grammatical errors should be corrected at revision, so please note any specific errors here.

Reviewer #1: No

Reviewer #2: No

6. Review Comments to the Author

**Reviewer #1:**

**Comment1** :

The manuscript has been improved a little, but many of my previous issues are not solved. The study question is still very vague “Investigate the performance of a survey methods and compare the conservation value of registered and deregistered WHKs.” I commented this already during the first revision, stating “You should develop more clear study questions. What is meant by performance? What is the “Conservation value”? Number of species? “We investigate” is not good enough, you need to specify what you want to get out of the research, and make sure the reader understands why this is important.” In the revised manuscript, instead of developing more clear questions, you have added “In this study…” (L. 143 in the tracked-changes-document). A clear question would be for example: ”We asked whether de-registered WKHs have lower conservation value in terms of the 1) number of conservation trees, 2) redlisted species and xxx”. I don’t really understand how “performance” is investigated, as you have nothing to compare in reagrds to performance.

**Response 1:**

Thank you for this valuable comment. We agree that the initial phrasing of the study question lacked clarity, and we have addressed this issue by refining and specifying our research aims more clearly in the revised manuscript.

At lines 174–183 in the tracked-changes version, we have added the following paragraph to clarify the research objectives and ensure that the reader understands the focus and relevance of our study:

*“ In this study, we evaluate the performance of the newly developed survey method for WKH in northwestern Sweden and compare the conservation value of currently registered WKH with that of recently deregistered ones. Our three main aims are ;*

1. *Compare registered and deregistered WKH through field inventories based on the following criteria in the new method for WKH; number of conservation trees, number of old trees, number of deadwood and number of signal and red-listed species.*
2. *Quantify the potential consequences of changes in inventory methodology and the ongoing deregistration of these valuable forest areas by analyzing harvest records and pre-harvest assessments within deregistered WKH.*
3. *Illustrate the potential long-term impact of deregistration of WKH on forest conservation values and biodiversity. “*

**Comment 2**:
The methods section has been improved slightly, but still I don’t know how the inventory was done. How large are the sample plots?

**Response 2**:

Thank you for pointing this out. We have now added additional information to clarify how the inventory was conducted.

At lines 223–227, we added the following description to specify the size and structure of the sample plots:

*“ For each forest area there is a number of sample sites, at each sample site an inventory is done following the checklist. The sample sites are circular plots with a radius of 25 meters, corresponding to an area of approximately 0.2 hectares. All recorded objects within each plot were multiplied by 5 to estimate the number of objects per hectare. The checklist has a list of criteria and a forest is defined as a likely WKH if a certain number of criteria are met.”*

We also expanded on the method at lines 241–247 to clarify how the classification is applied:

*“Within the checklist there are two different routes to reach the WKH classification status for a forest area (Track 1 and Track 2): in Track 1, a few high-value criteria suffice, while, in Track 2, a longer list of lower-value criteria is required. Track 1 and Track 2 are closely related but can be assessed independently. Track 1 includes key criteria that define forests with high conservation value; if enough are met, the area is likely classified as a WKH. Each criterion in Track 1 generally carries more weight than those in Track 2. Track 2 includes supporting factors that strengthen the likelihood of WKH classification.”*

And further explained some important details from the method at L.255-259

*“The new method favors large areas and areas in proximity to other WKH to create a green infrastructure. According to the new method, a so-called “50/50 rule” can be applied in a continuous forest landscape characterized by high conservation values, especially when borders are hard to delimit [11]. This rule dictates that if 50% of the area reaches the criteria for WKH standard, the entire area should be designated as a WKH.”*

We hope these additions sufficiently address the reviewer’s concerns about the inventory method.

**Comment 3**:

I checked the supplementary files, and found them to be rather bad, partly in Swedish and partly English, and not understandable (Table S1: NB1, NB2…, Table S2: KH1, KH2,…, in the figure 2. R1,R2, …,D9. The addition in Line 185-187 does not help at all.
You mention in your reply that you have corrected “habitat” to “WKH” in the methods section 2.3., but it still says habitat and I was again puzzled. “Habitat” is your individual stand, right? Then it would not help to change it to “WKH”, I would be as puzzled. WKH ID or Forest stand ID could work instead.

**Response 3:** Thank you for this valuable feedback, which has led to improvements in the supplementary files. We have made the supplementary files more consistent and easier to understand. To guide the reader, we have added a clearer description at lines 913–918:

*“S1. Table. Coordinates of plots . Table S1 Shows a list of all forest areas with number of sample sites and coordinates for all sample sites. Forest areas are named R1- R9 for the registered WKH and D1 – D9 for the unregistered WKH. Sample plots within each area are labeled plot1 – plot x, depending on the number of plots.*

*S2. Table. Results from field survey. Table S2 shows the full result from the field survey. Including all parameters found at each sample site within all forest areas.“*

We have corrected the language issues and clarified the labeling of forest areas in the supplementary files. All registered WKH are labeled **R1–R9**, while all deregistered WKH are labeled **D1–D9**. All sample plots within each forest area are labeled **plot1–plotX**, depending on the number of plots in that forest area.

We are also grateful for the comment regarding the use of "habitat" and forest IDs. To improve clarity, we have replaced "habitat" with **forest area** throughout the manuscript. Each examined WKH is now referred to as a forest area, and the plots within them as sample plots. These changes have been implemented throughout the Results section.

**Comment 4:**
Later you say (in the tracked changes version L 210—216) that you fitted glm with registration status and habitat as independent variables. But you had only one observation of redlisted species per habitat/stand, right? And then you used chi-square test? Why? I really don’t follow, and you did not provide answers in your response letter, even though this question was asked already in the first round. You have also deleted important information from the text, the Poisson error distribution. Or didn't you finally use Poisson? Also, it would be important to check the model fit in all cases. You had changed from glmm to linear mixed model, why? Number of trees, number of species or number of deadwood pieces as a response variable requires Poisson error distribution, most probably, which makes lmm an incorrect method. At this point, I don’t know what kind of analyses you have finally conducted, it is so unclear that I totally lost it.

**Response 4:** Thank you for your insightful comments regarding our modeling approach, we apologize for the lack of clarity in the previous version. We have now revised the methods section to clearly describe the final statistical models used.. Initially, we employed linear mixed-effects models (LMMs) to analyze parameter 1-3. However, diagnostic checks revealed violations of assumptions including non-normality of residuals and heteroscedasticity, undermining model validity. Subsequently, we tested Poisson GLMMs to better suit count data, but these models exhibited significant overdispersion, as indicated by formal statistical tests. To address this, we adopted negative binomial GLMMs, which explicitly model overdispersion and retain the hierarchical structure through random effects.The negative binomial GLMMs provided a more appropriate fit, with residual diagnostics indicating improved adherence to model assumptions.

Thank you also or pointing out the confusion regarding the analysis the difference in redlisted and signal species We have now clarified that for species counts there is only one observation per forest. Our earlier mention of habitat in this context has been removed. We first fitted a Poisson GLM to test for overdispersion (dispersion ratio = 2.54), which led us to adopt a negative binomial GLM. To look at model fit we did residual diagnostics (Q–Q plots and histograms).

All changes are explained in the method section at L. 261-278

“To assess differences between registered and deregistered WKHs, we focused on four key inventory parameters present in all checklists: (1) number of conservation trees, (2) number of old trees, (3) number of deadwood objects, and (4) number of signal and red-listed species.

For parameters 1–3, which were measured across multiple plots within each forest area, we fitted generalized linear mixed models (GLMMs) with a negative binomial error distribution using the glmmPQL function from the MASS package in R [29]. Registration status (registered vs. deregistered) was included as a fixed effect, and forest area as a random effect to account for the nested sampling structure (plots within forest areas). Initially, we fitted Poisson GLMMs using the glmer function in the lme4 package [30]. However, overdispersion (where variance exceeds the mean, violating Poisson assumptions) was detected based on Pearson residuals [31] (dispersion ratio > 2), prompting the use of a negative binomial distribution. For parameter 4 (number of red-listed signal species), only one number per forest was collected, so we fitted a negative binomial generalized linear model (GLM) with registration status as the predictor. A Poisson GLM was initially tested, but again overdispersion was evident (dispersion ratio > 2), warranting the negative binomial model.

Model diagnostics, including Q–Q plots and residual histograms were used to assess model fit for all parameters. All statistical analyses were conducted using the software R [32] and R-studio Version 12.0+353 [33]. To study the frequency of harvesting in these areas, QGIS was used to analyze maps of conducted harvesting from the Swedish forest agency together with maps of deregistered WKHs. Geographical analysis was performed using QGIS (3.16.15 – Hannover) and the geoprocessing tool intersection. Geographical data of harvesting operations carried out within deregistered key habitats after the deregistration date were combined, and the intersecting area was calculated. The data of harvesting operations from SFA span from 2010-01-01 to 2023-03-02, but only data of harvesting operations from dates after deregistration of key habitats included in this study were analyzed [34].”

**Comment 5:** I said earlier that your R and packages must be cited. Version is not enough. This remains an issue.

**Response 5:** Thank you for this comment. We are sorry that this was not adjusted in response to the previous comment. The issue has now been corrected, as shown above at lines 261–278.

**Comment 6:** The first part of the results is still very discussive, not much has changed. You mention that you have added a lot of text of statistical analyses in the results, but I see barely any, some p-values and Variance values are added. No real results are shown for the models, stating just p-vales is not sufficient in reporting the results.

**Response 6:** Thank you for your valuable feedback. We appreciate the need for more reporting of the statistical results in the Results section. To address this, we have now expanded the description to include full model outputs, such as estimates, standard errors, and relevant model diagnostics, beyond just p-values and variance components. This provides a clearer picture of effect sizes and model fit. We also ensured that the statistical results are presented objectively without interpretation, reserving discussion for the Discussion section. We hope these additions improve the clarity of the Results.

**Comment 7:** Boxplots are not a correct visualization method when n<5 (which I see is true in many of the forest stands (Supplementary tables 1 and 2)). I did not realize this low n earlier.

**Response 7:** Regarding visualization, we agree that boxplots are unsuitable for groups with fewer than five observations. We have replaced this boxplot with dot plots overlaid on model‐predicted means and SD, which more accurately represent the data distribution regardless of sample size (Figure 3). L.352

**Comment 8:** The sentence in the results: “These findings suggest that registration status significantly influences deadwood levels, with habitat-specific variation also playing a role.”, is not true. How would the registration status affect the levels of deadwood? If the status is changed from registered to de-registered, would the deadwood amount suddenly decrease? This is a common wording failure, be careful! Further, the sentence would belong to the discussion, not results.

**Response 8:** Thank you for this helpful observation. We fully agree that the original phrasing could be misinterpreted as implying a causal relationship between registration status and deadwood abundance, which was not our intention. We have revised the sentence in the Results section to ensure it remains descriptive and avoids causal language. L.404-408

*“The average number of deadwood objects per site exceeded the threshold in all areas except D2, where the mean density was 62 objects per hectare—just above the required criterion. The negative binomial GLMM revealed a statistically significant positive effect of registration status on the total number of deadwood objects per hectare (p < 0.001), indicating that registered WKHs contained significantly more deadwood than deregistered sites. Model diagnostics, including Q–Q plots and Pearson residual histograms, supported the adequacy of model fit and confirmed that overdispersion was appropriately handled by the negative binomial structure. These results suggest that registration is associated with the retention of higher deadwood quantities.”*

**Comment 9:**
Discussion is still vague. There are many methods to evaluate conservation values of forests in Europe, these are not mentioned and the results are not discussed with the light of those findings. Most of the articles cited in the manuscript are Swedish reports by SFA, which shows that the manuscript may not reach wider, international audience.

**Response 9:** Thank you for this valuable comment. We agree that broader context is essential for an international audience. In response, we have added a few more relevant international scientific references. For example one that analyzes the variation in WKH methods and criteria across Europe. This is now included in the reference list and has been integrated into the discussion at L. 486

L. 486, we now state:
“The revised inventory method demonstrates improved structure and aims to enhance transparency, objectivity, and predictability. It builds on three key categories used in WKH inventories across Europe: stand-level structural features, presence of individual habitat elements, and presence of indicator species [44].

L. 467:
“The result may be increased fragmentation, diminished ecological connectivity, and reduced resilience in boreal forest ecosystems—trends that are already recognized as key drivers of biodiversity decline and ecosystem degradation globally [40,41].”

These are examples of additions to help position our research within the broader European framework and support a more internationally relevant discussion.

**Comment 10:**

The discussion is not fully supported by the results, for example the issue of snow cover comes very surprisingly up in the discussion, as the timing of the inventories is not addressed anywhere in the results or elsewhere. For the reader that knows Sweden and is familiar with the system, this may make sense, but not to anyone else.

**Response 10:**

Thank you for highlighting this important point. We agree that the discussion of snow cover and inventory timing was not sufficiently supported in the previous version of the manuscript. To clarify this connection for all readers, we have now added a sentence in the Methods section (L. 200) that specifies the timing of our field inventories and explains how snow cover can impact data collection. This addition provides a clearer foundation for the discussion regarding regional applicability of the method in snowy areas of northern Sweden. We also find it relevant to discuss this issue since the method is developed to take regional differences into account.

L.200 “Our field survey was conducted during snow-free conditions to ensure full visibility of the forest floor.”

L491 “Although tailored for northwestern Sweden, the method does not explicitly address practical challenges such as snow cover. In our study, inventories were conducted during snow-free periods, which is essential for reliably identifying ground-level features and species. However, under current regulations, the six-week notification period for clear-cutting may result in inventories being conducted while snow still covers key forest floor indicators required by the inventory method. Extending the notification period to ensure at least six snow-free weeks —or explicitly stating that inventories requires snow free conditions could — significantly improve accuracy in northern regions.”

**Comment 11**:
Finally, the manuscript was earlier given a “major revision” and you were given lots of comments how to improve the manuscript. Unfortunately, I don’t see much improvement, many of the issues remain and the sloppiness in writing and referring to tables and figures continues.

**Response 11:**

We are grateful for the opportunity to revise the manuscript once more and have made substantial efforts to further improve it. In this version, we have focused on enhancing the language and overall structure to ensure greater clarity and readability. We also aimed to make our research objectives more clearly defined. We sincerely appreciate the thoughtful and constructive comments from the reviewers, which have been very helpful in strengthening the manuscript.

**Comment 12:**

Please, next time you submit this manuscript, pay attention to actually do the changes and not just comment that you have done them. Make sure to using spelling check to correct all the typos, there are lots of typos in the text which gives a sloppy impression of the author(s). In the discussion, there are sections where nearly every sentence has a typo.

**Response 12:**

Thank you for pointing this out. We sincerely apologize for the oversight and fully understand the importance of presenting a polished and carefully proofread manuscript. In this revised version, we have thoroughly reviewed the entire text, with special attention to spelling, grammar, and clarity. We have corrected all identified typos and used a combination of spelling and grammar tools along with manual proofreading to ensure a higher standard of language throughout the manuscript. We appreciate your patience and the opportunity to improve the quality of our submission.

**Comment 13:**
-Introduction claims that Sweden would be 4000 km long, which is not true.

**Response 13:**

Thanks for this comment, this error has been adjusted at L.136-139

*“ Applying the same method for WKH inventories across the whole country can be problematic, as the forest landscapes, which span 1,570 km from north to south, show substantial regional variations. “*

**Comment 14:**
-Make sure to write scientific names with capitalized Genus, and using italics (Table 1).

**Response 14:**

Thanks for pointing this out, this has been adjusted in Table 1 at L. 304

| **ID** | **Criteria track 1** | **Criteria track 2** | **deregistered (year)** | **Class-**  **At time of survey** | **Survey result** | **Red-listed and signal species (classification from red list)** |
| --- | --- | --- | --- | --- | --- | --- |
| R1 | 6/6 | 18/22 |  | WKH | WKH | *Phlebia mellea* (VU), *Dryocopus martius* (NT), *Lobaria pulmonaria* (NT), *Lobaria scrobiculata* (NT), *Alectoria sarmentosa* (NT), *Phellinus chrysoloma* (NT), *Nephroma bellum* (LC) |
| R2 | 6/6 | 15/22 |  | WKH | WKH | *Phellinus chrysoloma* (NT), *Alectoria sarmentosa* (NT), *Fomitopsis rosea* (NT) |
| R3 | 5/6 | 16/22 |  | WKH | WKH | *Haploporus odorus* (VU), *Diplomitoporus lenis* (VU), *Antrodia albobrunnea* (VU), *Alectoria sarmentosa* (NT), *Lobaria pulmonaria* (NT), *Dryocopus martius* (NT), *Phellinus chrysoloma* (NT), *Lobaria scrobiculatus* (NT), *Nephroma bellum* (LC), *Nephroma resupinatum* (LC), *Icmadophila ericetorum* (LC) |
| R4 | 6/6 | 15/22 |  | WKH | WKH | *Alectoria sarmentosa* (NT), *Phellinus chrysoloma* (NT), *Picoides tridactylus* (NT), *Pseudographis pinicola* (NT) |
| R5 | 6/6 | 12/22 |  | WKH | WKH | *Alectoria sarmentosa* (NT), *Phellinus chrysoloma* (NT), *Lobaria scrobiculata* (NT), *Icmadophila ericetorum* (LC) |
| R6 | 5/6 | 14/22 |  | WKH | WKH | *Alectoria sarmentosa* (NT), *Dryocopus martius* (NT), *Cystostereum murrayi* (NT), *Phellinus chrysoloma (NT), Lobaria scrobiculatus* (NT), *Nephroma resupinatum* (LC) |
| R7 | 6/6 | 16/22 |  | WKH | WKH | *Phellinus chrysoloma* (NT), *Alectoria sarmentosa* (NT), *Perisoreus infaustus* (LC), *Matteuccia struthiopteris* (LC) |
| R8 | 6/6 | 18/22 |  | WKH | WKH | *Fomitopsis rosea* (NT), *Leptoporus mollis* (NT), *Picoides tridactylus* (NT), *Alectoria sarmentosa* (NT), *Pseudographis pinicola* (NT), *Phellinus chrysoloma* (NT), *Perisoreus infaustus* (LC), *Nephroma bellum* (LC) |
| R9 | 6/6 | 16/22 |  | WKH | WKH | *Cystostereum murrayi* (NT), *Alectoria sarmentosa* (NT), *Phellinus chrysoloma* (NT), *Onnia leporina* (NT), *Perisoreus infaustus* (LC), *Icmadophila ericetorum* (LC), *Aconitum lycoctonum* (LC), *Icmadophila ericetorum* (LC), *Nephroma resupinatum* (LC) |
| D1 | 6/6 | 16/22 | 2015 | promise | WKH | *Alectoria sarmentosa* (NT), *Phellinus chrysoloma* (NT), *Icmadophila ericetorum* (LC) |
| D2 | 2/6 | 12/22 | 2017 | production | WKH | *Haploporus odorus* (VU), *Skeletocutis odora* (VU), *Phellinidium ferrugineofuscum* (NT), *Carbonicola anthracophila (*NT), *Dryocopus martius* (NT), *Pseudographis pinicola* (NT), *Alectoria sarmentosa* (NT), *Phellopilus nigrolimitatus* (NT), *Onnia leporina* (NT), *Nephroma resupinatum* (LC) |
| D3 | 6/6 | 18/22 | 2015 | promise | WKH | *Poecile cinctus* (VU), *Alectoria sarmentosa* (NT), *Phellinus chrysoloma* (NT), *Onnia leporina* (NT), *Picoides tridactylus* (NT), *Nephroma bellum* (LC), *Nephroma resupinatum* (LC), *Meruliopsis taxicola* (LC) |
| D4 | 6/6 | 18/22 | 2015 | promise | WKH | *Skeletocutis odora* (VU), *Fomitopsis rosea* (NT), *Phellinus chrysoloma* (NT), *Alectoria sarmentosa* (NT), *Phellinidium ferrugineofuscus* (NT), *Leptoporus mollis* (NT), *Perisoreus infaustus* (LC), *Icmadophila ericetorum* (LC) |
| D5 | 6/6 | 15/22 | 2015 | promise | WKH | *Alectoria sarmentosa* (NT), *Phellinidium ferrugineofuscus* (NT), *Fomitopsis rosea* (NT), *Onnia leporina* (NT), *Phellinus chrysoloma* (NT), *Phellopilus nigrolimitatus* (NT), *Icmadophila ericetorum* (LC) |
| D6 | 5/6 | 18/22 | 2015 | promise | WKH | *Antrodia albobrunnea* (VU), *Diplomitoporus lenis* (VU), *Skeletocutis odora* (VU), *Phlebia mellea* (VU), *Laurilia sulcata* (VU), *Onnia leporina* (NT), *Alectoria sarmentosa* (NT), *Phellinus chrysoloma* (NT), *Pseudographis pinicola* (NT), *Cystostereum murrayi* (NT), *Leptoporus mollis* (NT), *Phellinidium ferrugineofuscum* (NT), *Lobaria pulmonaria* (NT), *Fomitopsis rosea* (NT), *Phellopilus nigrolimitatus* (NT), *Picoides tridactylus* (NT), *Hypogymnia bitteri* (NT), *Icmadophila ericetorum* (LC), *Meruliopsis taxicola* (LC), *Perisoreus infaustus* (LC), *Nephroma bellum* (LC) |
| D7 | 6/6 | 13/22 | 2015 | promise | WKH | *Phellinus ferrugineofuscus* (NT), *Alectoria sarmentosa* (NT), *Onnia leporina* (NT), *Fomitopsis rosea* (NT), *Nephroma bellum* (LC) |
| D8 | 6/6 | 16/22 | 2015 | promise | WKH | *Laurilia sulcata* (VU), *Phellinidium ferrugineofuscum* (NT), *Fomitopsis rosea* (NT), *Cystostereum murrayi* (NT), *Phellinus chrysoloma* (NT), *Phellopilus nigrolimitatus* (NT), *Alectoria sarmentosa* (NT), *Picoides tridactylus* (NT), *Icmadophila ericetorum* (LC), *Perisoreus infaustus* (LC) |
| D9 | 6/6 | 15/22 | 2015 | promise | WKH | *Alectoria sarmentosa* (NT), *Phellinus chrysoloma* (NT), *Pseudographis pinicola* (NT), *Nephroma bellum* (LC) |

**Comment 15**:
-You still have “human disturbance” as your result without ever mentioning it in the introduction or methods or showing any results regarding this. I was as puzzled this time as well. If you present a result of visible signs of human disturbance, it must be stated in the methods.

**Response 15:**

Thank you for pointing this out. Since the official method description from the Swedish Forest Agency spans 82 pages, it has been challenging to condense it without omitting important aspects. Based on your helpful input, we have now added a clarification in the methods section (L. 254–257):

*“ The classification of a WKH involves a comprehensive assessment of both the forest stand and the surrounding landscape. In addition to quantifying specific parameters, the survey should also document signs of human disturbance and other features that may influence the forest's conservation value.”*

This addition ensures that the inclusion of human disturbance in the results is grounded in the described methodology.

**Comment 16:**
-In the text, after Figure 1 comes Figure 4, then 2. Please make sure all the tables and figures are correctly numbered and referred. They are not.

**Response 16:**

Thank you for pointing this out, this has now been adjusted and the Figures are referred to in the correct order in the result section.

“Additionally, the number of spruce conservation trees was above the criteria in all forests, with at least 30 conservation trees per hectare (Figure 2). The negative binomial GLMM indicated a positive but marginally non-significant effect of registration status on the number of conservation trees per hectare (p = 0.086), suggesting that registered WKHs tend to have more conservation trees than deregistered ones, although the difference was not statistically significant. The model included forest area as a random effect to account for variability among sites. Model diagnostics, including Q–Q plots and Pearson residual histograms, supported the adequacy of model fit and confirmed that overdispersion was appropriately handled by the negative binomial structure. No significant difference was found in the number of distinct signal and red-listed species between registered and deregistered forests (p = 0.372; Figure 3). The total number of deadwood objects exceeded the set criteria of a minimum of 60 dead trees per hectare in Track 1 for all areas, except for WKH D2, which had a relatively low number of deadwood objects per hectare (Figure 4).”

**Comments from the track changes manuscript from Reviewer #1**

Below, we respond to the comments from Reviewer #1 line by line. Line numbers refer to the tracked changes version of the manuscript. Comments on grammar and spelling has been adjusted without any added comment below.

***Comment 17:*** *L.45*  *Extensive primary boreal forests within Europe are primarily located in northwestern Sweden, Finland and Russia.* What about Norway?

**Response 17:**

*Good comment, we included Norway in the statement as a part of Fennoscandia.*

*“Extensive primary boreal forests within Europe are primarily located in Fennoscandia and northwestern Russia.”*

**Comment 18:** at least provide a short argument for deregistration.L.51

**Response 18**: The argument for deregistration vary from case to case and we feel that the explanation for this is too complex to be part of the abstract. However, we have tried to clarify this sentence within the limits of an abstract.

*“Recently, many WKH were deregistered and thereby lost their potential protection against clear-cutting, jeopardizing biodiversity values in these forests.”*

**Comment 19**: inventory of what? L.52

**Response 19:** Thank you for the comment. We have clarified that the field inventory specifically concerns Woodland Key Habitats (WKHs). The revised sentence now reads:
“To assess the leniency and effects of deregistration, we conducted a field inventory of Woodland Key Habitats (WKHs) using a novel method proposed by the Swedish Forest Agency, which features more stringent criteria for WKH classification in northwestern Sweden.”

**Comment 20:** which criteria?L.55

**Response 20:** Spelling misstake fixed and clarified wich criteria we mean.

*“Our inventory results show that all 18 areas reach the criteria for WKH with the new method, despite the higher thresholds for conservation values. “*

**Comment 21:** If you don´t say in which temporal interval it is difficult to assess the entity of this cutting. Is this a lot? Which % of the total? L.59

**Response 21:** More information about the time intervall has been added to adress this comment. *“ Analysis of recent harvest actions within deregistered WKH in Sweden indicated that over 800 hectares of clear-cutting occurred within these areas 1-7 years post deregistration.”*

**Comment 22:** Is this recommendation connected with deregistered WKH? What´s the difference? L. 61

**Response 22:** Thank you for pointing this out. We agree that the sentence needed clarification. We have revised the sentence to point out the importance of keeping areas of high value as registered WKH as the registration can stop these areas from beeing cut.

“It is crucial to exclude areas with WKH qualities from forestry practices, in order to preserve biodiversity in boreal forests since they harbor redlisted species.”

**Comment 23 :L82**.

**Response 23:** Great input. It’s not necessary with this word. This has been removed.

**Comment 24 : L130.**

**Response 24:** We are thankful for the comment here and have corrected the distance.

**Comment 25 L191.**

**Response 25:** Good input, this has been changed to study area instead of map.

**Comment 26: L203.**

**Response 26:** Thanks for the input on cavities for birds. This is a more precise description and has been changed.

“The term “conservation tree” is defined in the method by the SFA as a tree with significant importance for biodiversity, such as trees older than 250 years, trees with hanging lichens, or those with suitable cavities for cavity-nesting birds.”

**Comment 27 :L.211.**

**Response 27:** the sentence has been changed to better explain the concept of how to register signal species.

“Species do not need to be found within the sample site to be included, but is also added to the invetory list if seen inbetween sample sites within the forest area. All signal and red-listed species are included in the overall assessment of the key biotope [15]. “

**Comment 28 : L.216**. This is not clear and must be better explained. *For example, in track 1, having 30 conservation spruce trees per hectare is adequate to reach one of the six*

*criteria, whereas in track 2, 20 conservation spruce trees per hectare is required to reach one of the 22*

*criteria.*

***Response 28:*** Thank you for the comment. The issue regarding the lack of clarity in the methods section was addressed in a previous comment, but we have further clarified it here as follows:

“Within the checklis there are two different routes to reach the WKH classifiaction staus for a forest area (Track 1 and Track 2): in Track 1, a few high-value criteria suffice, while, in Track 2, a longer list of lower-value criteria is required. Track 1 and Track 2 are closely related but can be assessed independently. Track 1 includes key criteria that define forests with high conservation value; if enough are met, the area is likely classified as a WKH. Each criterion in Track 1 generally carries more weight than those in Track 2. Track 2 includes supporting factors that strengthen the likelihood of WKH classification.”

**Comment 29 :L231.**

**Response 29:** References to R and R studio has been adjusted and corrected.

**Comment 30 : L258.**

**Response 30:** Latin names has been correctly adjusted.

**Comment 31: L297** what is the interior here? *A majority of the interior of the surveyed forests displayed no clear indications of human disturbance and had clearly never undergone large-scale exploitation.*

**Response 31:** Thank you for the comment. We have revised the sentence to improve clarity as follows: ”A majority of the surveyed forests displayed no clear indications of human disturbance and appeared never to have undergone large-scale exploitation.”

**Comment 32:** **L314.** p-value is not enough. TO which test do you refer to? Are theese results from GLMs? Or what?

**Response 32:** Thank you for this valuable comment, we have done a lot of changes within the statistical method part at described above and consequently changed the result section according to this as well. We hope that these changes makes it clear what we did and why in both method section and result section. Here is an example from L. 330 describing the differences we made regarding the comment above.

“All surveyed forests exceeded the set criteria of a minimum of 20 conservation trees per hectare for Track 1. Additionally, the number of spruce conservation trees was above the criteria in all forests, with at least 30 conservation trees per hectare (Figure 2). The negative binomial GLMM indicated a positive but marginally non-significant effect of registration status on the number of conservation trees per hectare (p = 0.086), suggesting that registered WKHs tend to have more conservation trees than deregistered ones, although the difference was not statistically significant. The model included forest area as a random effect to account for variability among sites. Model diagnostics, including Q–Q plots and Pearson residual histograms, supported the adequacy of model fit and confirmed that overdispersion was appropriately handled by the negative binomial structure. “

**Comment 32:** **L342.** m3 or what? *The total average amount of dead wood (both standing and lying) in all areas was 192 per hectare and is well above the citeria in the method, 50/ hectare in track one and 60/ hectare in track two (See Figure 4)*

***Response 32****:* Thank you for the comment. We have clarified that the value refers to the number of dead-wood objects (not m³), and we have also corrected the spelling errors in the sentence. The revised sentence now reads:

“ The average number of dead-wood objects (both standing and lying) across all areas was 192 trees per hectare, which is well above the criteria of 50/ hectare in Track 1 and 60/ hectare in Track 2 (Figure 4).”

**Comment 33: L391.** This is not a Swedish journal.

**Response 33:** Thank you for pointing this out. This line was included by mistake and has now been removed from the manuscript. We apologize for the oversight.

**Reviewer #2:**

**Comment 1**:

The paper has improved from the previous version but still needs major revision and strong review of the way results are expressed and summarized. Statistical tests must be better reported. English language must be improved. The topic of the paper is important and deserves more efforts from tehe authors to reach a publication level.

**Response 1:**

Thank you for recognizing the importance of the topic and for your constructive feedback. We appreciate your belief in the potential of this paper. In response to your comments, we have made substantial improvements to both the language and grammar throughout the manuscript. Additionally, we have revised the reporting of statistical methods and results to ensure greater clarity and transparency.

All changes are explained in the method section at L. 263-288

“To assess differences between registered and deregistered WKHs, we focused on four key inventory parameters present in all checklists: (1) number of conservation trees, (2) number of old trees, (3) number of deadwood objects, and (4) number of signal and red-listed species.

For parameters 1–3, which were measured across multiple plots within each forest area, we fitted generalized linear mixed models (GLMMs) with a negative binomial error distribution using the glmmPQL function from the MASS package in R [29]. Registration status (registered vs. deregistered) was included as a fixed effect, and forest area as a random effect to account for the nested sampling structure (plots within forest areas). Initially, we fitted Poisson GLMMs using the glmer function in the lme4 package [30]. However, overdispersion (where variance exceeds the mean, violating Poisson assumptions) was detected based on Pearson residuals [31] (dispersion ratio > 2), prompting the use of a negative binomial distribution. For parameter 4 (number of red-listed signal species), only one number per forest was collected, so we fitted a negative binomial generalized linear model (GLM) with registration status as the predictor. A Poisson GLM was initially tested, but again overdispersion was evident (dispersion ratio > 2), warranting the negative binomial model.

Model diagnostics, including Q–Q plots and residual histograms were used to assess model fit for all parameters. All statistical analyses were conducted using the software R [32] and R-studio Version12.0+353 [33]. To study the frequency of harvesting in these areas, QGIS was used to analyze maps of conducted harvesting from the Swedish forest agency together with maps of deregistered WKHs. Geographical analysis was performed using QGIS (3.16.15 – Hannover) and the geoprocessing tool intersection. Geographical data of harvesting operations carried out within deregistered key habitats after the deregistration date were combined, and the intersecting area was calculated. The data of harvesting operations from SFA span from 2010-01-01 to 2023-03-02, but only data of harvesting operations from dates after deregistration of key habitats included in this study were analyzed [34].

”

7. PLOS authors have the option to publish the peer review history of their article ([what does this mean?](https://track.editorialmanager.com/CL0/https:%2F%2Fjournals.plos.org%2Fplosone%2Fs%2Feditorial-and-peer-review-process%23loc-peer-review-history/1/010f0196635ba12e-7dce4a2f-fab2-4d9e-b7d5-3bb3627c5b9e-000000/0ulgMJ-0jMQjJUvk2ocl5ztlosNXwFDubpU2pIFmeqA=208)). If published, this will include your full peer review and any attached files.


If you choose “no”, your identity will remain anonymous but your review may still be made public.


**Do you want your identity to be public for this peer review?**For information about this choice, including consent withdrawal, please see our [Privacy Policy](https://track.editorialmanager.com/CL0/https:%2F%2Fwww.plos.org%2Fprivacy-policy/1/010f0196635ba12e-7dce4a2f-fab2-4d9e-b7d5-3bb3627c5b9e-000000/mEKtDQvCP2SbKAoj270sCOlAhT0ZmSzRt9N1qQ_gns4=208).

Reviewer #1: No

Reviewer #2: No

While revising your submission, please upload your figure files to the Preflight Analysis and Conversion Engine (PACE) digital diagnostic tool, [https://pacev2.apexcovantage.com/](https://track.editorialmanager.com/CL0/https:%2F%2Fpacev2.apexcovantage.com%2F/1/010f0196635ba12e-7dce4a2f-fab2-4d9e-b7d5-3bb3627c5b9e-000000/9zyOpoA88VLKSxI-iZpc-22Ns3kt33TTRxukBBwy3QE=208). PACE helps ensure that figures meet PLOS requirements. To use PACE, you must first register as a user. Registration is free. Then, login and navigate to the UPLOAD tab, where you will find detailed instructions on how to use the tool. If you encounter any issues or have any questions when using PACE, please email PLOS at [figures@plos.org](mailto:figures@plos.org). Please note that Supporting Information files do not need this step.
